# In vitro and in vivo burn healing study of standardized propolis: Unveiling its antibacterial, antioxidant and anti-inflammatory actions in relation to its phytochemical profiling

**DOI:** 10.1371/journal.pone.0302795

**Published:** 2024-05-14

**Authors:** Dina M. El-Kersh, Rania F. Abou El-Ezz, Eman Ramadan, Reham F. El-kased

**Affiliations:** 1 Faculty of Pharmacy, Pharmacognosy Department, The British University in Egypt, Cairo, Egypt; 2 Faculty of Pharmacy, Pharmacognosy Department, Misr International University, Cairo, Egypt; 3 Faculty of Pharmacy, Department of Pharmacology and Toxicology, The British University in Egypt, Cairo, Egypt; 4 Center for Drug Research and Development (CDRD), The British University in Egypt, Cairo, Egypt; 5 Faculty of Pharmacy, Department of Microbiology and Immunology, The British University in Egypt, Cairo, Egypt; Cairo University, Faculty of Science, EGYPT

## Abstract

**Background:**

Natural propolis has been used since decades owing to its broad-spectrum activities. Burn injuries are a global health problem with negative impacts on communities. Bacterial infections usually accompany burns, which demand implementation of antibiotics. Antibiotics abuse led to emergence of microbial drug resistance resulting in poor treatment outcomes. In such instances, the promising alternative would be natural antimicrobials such as propolis.

**Objective:**

Full chemical profiling of propolis and evaluation of in vitro antibacterial, antioxidant and anti-inflammatory activities as well as in vivo burn healing properties.

**Methods:**

Chemical profiling of propolis was performed using Liquid chromatography (UHPLC/MS-PDA and HPLC-PDA). In vitro assessment was done using Disc Diffusion susceptibility test against *Staphylococcus aureus* and infected burn wound mice model was used for in vivo assessment. In vitro antioxidant properties of propolis were assessed using DPPH, ABTS and FRAP techniques. The anti-inflammatory effect of propolis was assessed against lipopolysaccharide/interferon-gamma mediated inflammation.

**Results:**

UHPLC/MS-PDA results revealed identification of 71 phytochemicals, mainly flavonoids. Upon flavonoids quantification (HPLC–PDA), Pinocembrin, chrysin and galangin recorded high content 21.58±0.84, 22.73±0.68 and 14.26±0.70 mg/g hydroalcoholic propolis extract, respectively. Propolis showed concentration dependent antibacterial activity in vitro and in vivo burn healing via wound diameter reduction and histopathological analysis without signs of skin irritation in rabbits nor sensitization in guinea pigs. Propolis showed promising antioxidant IC_50_ values 46.52±1.25 and 11.74±0.26 μg/mL whereas FRAP result was 445.29±29.9 μM TE/mg. Anti-inflammatory experiment results showed significant increase of Toll-like receptor 4 (TLR4), interleukin-6 (IL-6) and tumor necrosis factor-alpha (TNF-α) mRNA levels. Nitric oxide and iNOS were markedly increased in Griess assay and western blot respectively. However, upon testing propolis against LPS/IFN-γ-mediated inflammation, TLR4, IL-6 and TNF-α expression were downregulated at transcriptional and post-transcriptional levels.

**Conclusion:**

Propolis proved to be a promising natural burn healing agent through its antibacterial, antioxidant and anti-inflammatory activities.

## Introduction

Propolis (honeybee glue) is a natural resinous mixture of botanical balsams and resin with digestive enzymes of bees collected by honeybees *Apis mellifera*, from various plant sources. Propolis enriched with a myriad of natural pharmacologically active constituents such as polyphenols, terpenoids, steroids, and amino acids [1]. Propolis displays a broad spectrum of pharmacological and biological features *viz*. antimicrobial, anti-inflammatory, immunomodulatory, antioxidant, anticancer, antiulcer, antitumor, cardioprotective, neuroprotective and hepatoprotective actions [2]. The beneficial properties and chemical constitution of propolis differ greatly depending on the geographical origin, seasonal collection time, and botanical source [3].

Propolis has been used since ancient times in traditional medicine to keep good health, owing to its broad-spectrum activities [4]. Nowadays, it is used as a therapeutic agent to avoid different diseases and improve health [5].

Burn injuries are a major global public health problem given their high incidence and potentially devastating physical, psychosocial, and financial impacts on individuals, households, and communities [6–8]. Burns from fire, heat, and hot substances are the fourth most common type of civilian trauma worldwide, following road traffic incidents, falls, and interpersonal violence [9]. It is estimated that there are between 7 and 12 million people (up to 33,000 each day) who sustain burn injuries that require medical care, leading to prolonged absence from work or school, or result in death each year [10].

Initial burn wounds are sterile, but within a few days burns destroy the barrier between internal sterile tissue and the external environment leading to microbial penetration. Microbes can easily colonize burnt tissue as it represents a protein-rich environment favorable for microbial growth [11]. Gram positive bacteria start to colonize the burn sites from deeper structures (hair follicles and glands) leading to infection [12], if left untreated, this may be followed by a second phase, where Gram-negative bacteria such as *Pseudomonas aeruginosa*, *Escherichia coli*, *Acinetobacter baumannii*, and *Klebsiella* spp. become the predominant bacteria [13].

Healing of burn wounds is a complex, multi-step process prone to internal and external factors and implementation of antimicrobial therapy is often demanded. Antibiotics abuse has led over years to the emergence of microbial drug resistance leading to poor treatment efficiency. In such instances, the promising alternative is the use of natural antimicrobial, anti-inflammatory and antioxidant compounds, including plant extracts and those of bee origin, such as propolis [14–16].

The aim of this study is to assess the antibacterial effect and burn healing activity of hydroalcoholic extract of propolis in vitro and in vivo using infected burn-induced mouse model. In addition to studying the effect of propolis on TLR4 pathway through gene expression studies of key inflammatory intermediaries such as TLR4, TNF-α, IL-6 and iNOS. As antioxidant activity; propolis has been assesed in vitro using different techniques *viz*. 2,2-Diphenyl-1-picrylhydrazyl (DPPH) and 2,2′-azinobis (3- ethylbenzothiazoline-6-sulfonic acid) diammonium salt (ABTS) and ferric reducing antioxidant potential (FRAP) techniques.

A full chemical profiling of the total hydroalcoholic propolis extract was investigated by ultra-high performance liquid chromatography/mass spectroscopy-photodiode array detector (UHPLC/MS-PDA) in addition to standardization of the hydroalcoholic propolis extract using phenolics *viz*. gallic acid, quercetin, chrysin and galangin *via* high performance liquid chromatography–PDA (HPLC–PDA).

## Materials and methods

Raw propolis botanical source was from South Asia and it was obtained as dried raw powder from Imtenan Health Co., Cairo, Egypt. The authentics used in the standardization of the hydroalcoholic extract of propolis *viz*. pinocembrin, chrysin and galangin have been obtained from Sigma-Aldrich (St. Louis, Mo., USA). All the solvents used for the extraction and analysis met the quality criteria and in accordance with the international standards.

LPS (*Escherichia coli* 0111: B4) was obtained from Sigma Chemical Co. (St. Louis, MO, USA) (Cat No. L2630). Murine interferon-γ was purchased from PeproTech (Rocky Hill, NJ, USA) (Cat No. 315–05). High-glucose Dulbecco’s modified Eagle’s medium (DMEM) (Cat No. 41965–039) and fetal bovine serum (FBS) (Cat No. 10270–106) were obtained from Gibco. Isopropanol (HPLC grade; Cat No. BP26324), chloroform (HPLC grade; Cat No. C607SK-1), Dimethyl sulfoxide (DMSO; Cat No. 67-68-5) and ethanol (HPLC grade; Cat No. 64-17-5) were obtained from Thermo Fisher Scientific (Waltham, MA, USA). Revert Aid cDNA kit (Cat No. K1621), Maxima SYBR Green qPCR (Cat No. K0251) were all obtained from Thermo Fisher Scientific (Waltham, MA, USA) as well. The Griess assay kit (Cat No. G7921) was bought from Invitrogen (Carlsbad, CA, USA). Phosphate-buffered saline (PBS; 10X; Cat No. 17-516Q) and penicillin–streptomycin mixture (Cat No. 09-757F), and DMEM with 4.5 g/L glucose, without L-glutamine and without phenol red (Cat No. 12-917F) were obtained from Lonza Bioscience (Basel, Switzerland). MTT reagent (3-(4,5-dimethylthiazol-2-yl)-2,5-diphenyl tetrazolium bromide) (CT01-5 Sigma-Aldrich). The murine macrophage-like cell lines RAW 264.7 (ATCC-TIB71, Rockville, MD, USA) were obtained by the National Research Center (NRC) (Cairo, Egypt). QiAzol lysis buffer (Cat No. 79306) was purchased from Qiagen (Hilden, Germany). *Staphylococcus aureus*; gram positive bacteria; an isolated clinical strain known to localize burn wound infections was used in mouse model experiments.

### Extraction procedure for chromatographic analysis and biological study

Dried raw propolis powder (75 g) was extracted with hydro methanol (80%). The obtained extract was further concentrated on rotary evaporator to yield 45 g and then it was kept in -20°C for further analysis.

### UHPLC/MS-PDA analysis of propolis hydroalcoholic extract

The UHPLC/MS-PDA method of identification of secondary metabolites has been adopted from previous literature [17]. The negative HR-ESI and CID (collision induced dissociation) obtained from the mass spectrometer adjusted at 300 and 250°C of capillary and source heart temperatures, respectively, while the resolution of Fourier Transform Mass Spectrometry (FTMS) was 30.000 using inert N_2_ gas. The CID spectra (He gas as buffer) was recorded using normalized collision energy (NCE) of 35%. The separation using UHPLC was performed at 40°C, the injection volume was 2 μL and the flow rate was 150 μL/min. The gradient elution was carried out using solvent (A) water and (B) acetonitrile with 0.1% formic acid as follows: 0–1 min isocratic 5% (B), 1–11 min linear from 5 to 100% (B), 11–19 min, isocratic 100% (B) and 19–30 min, isocratic 5% (B).

The mass data were identified by XCalibur 2.2 SP1 software. Chemical constituents were identified by their UV–VIS spectra, retention times relative to external standards, mass spectra were compared to phytochemical library of natural products database mass bank (CRC press) and reference literature.

### HPLC-PDA standardization of propolis hydroalcoholic extract

The major flavonoids *viz*. pinocembrin, chrysin and galangin have been identified and quantified in the hydroalcoholic extract of propolis using Waters 2690 Alliance High performance liquid chromatography (HPLC) system equipped with a Waters 996 photodiode array detector (PDA). A Kromasil Eternity reversed phase column (C_18_) was used with specifications (4.6 mm, 250 mm, 5μm particle size). Elution of major flavonoids in the propolis extract was achieved using 0.1% formic acid in water as solvent (A), and methanol 100% as solvent (B). The total run time was 65 min where gradient elution was adopted. The elution run started by 80% of solvent (A) 0–5 min, 40% (5–45 min), then 20% (45–60 min), then finally back to 80% (60–65 min). The flow rate was 1 mL/min with injection volume 10 μL. The UV detection was set at 290 nm for all flavonoids [18].

### Standard calibration curves of propolis extract

The three authentic flavonoids were prepared separately *viz*. pinocembrin, chrysin and galangin in concentration 1 mg/mL (100% methanol). Serial dilutions (1–200 μg/mL) were then prepared for calibration curves. Quantification of the previous mentioned flavonoids in propolis extract was assembled using peak area (Y) and concentration (X) calculated as μg/mL, mean value (n = 3) ± standard deviation. The propolis extract was prepared in 1 mg/mL methanol using the following equations for the previous mentioned flavonoids: Y = 1.3184x – 1.4266 (R^2^ 0.999), Y = 1.2417x-0.3396 (R^2^ 0.999), Y = 0.9425x-1.7331 (R^2^ 0.998), respectively.

### In vitro antimicrobial evaluation of propolis hydroalcoholic extract

Two propolis concentrations were used in all in vitro and in vivo experiments. The two concentrations prepared contained 100% and 50% of propolis extract; where the 100% concentration was pure propolis extract with no water added and the second concentration 50% propolis was diluted with 50% water w/w.

### Disc diffusion susceptibility test

To test the antibacterial activity of the 100% and 50% w/w propolis; Disc Diffusion antibiotic susceptibility test (Kirby Bauer) was performed [19] using Mueller Hinton agar (Oxoid, UK). Bacterial suspension was prepared corresponding to 0.5 McFarland standard solution, where 5–6 bacterial isolate colonies were emulsified in sterile distilled water and the turbidity was adjusted to 1.5 × 10^8^ CFU/mL (equivalent to 0.5 McFarland standard) [20]. Following this step, a sterile cotton swab was used to inoculate the standardized bacterial suspension on the Muller Hinton agar plates evenly, then plates were left 5 min to dry. Filter paper discs (6 mm diameter—prepared according to Cheesbrough method [21] were immersed in both concentrations of propolis until saturation, then placed on the agar plates with light pressure to ensure complete contact with agar. A space of 15 mm was kept from the plates’ external margin to prevent overlapping of inhibition zones, a positive control was used (oxacillin disk 15 g). Plates were left for 15 min then incubated at 37°C for 3–5 days. After incubation, examination of plates took place and the zones of inhibition diameter were recorded.

### Minimum Inhibitory concentration (MIC) and Minimum Bactericidal concentration (MBC)

Broth dilution method was used to estimate the MIC of the tested propolis, where serial dilutions of propolis stock solution (2, 1, 0.5, 0.25 and 0.125 mg/mL) were prepared in ethanol followed by adding a drop of bacterial standard inoculum prepared using Mueller Hinton Broth (Difco^TM^). Then the test tubes were incubated at 37°C for 24 h followed by checking for turbidity to determine the MIC.

0.1 mL of the MIC test tubes showing no visible growth were sub-cultured on Mueller Hinton agar (Difco^TM^) plates to estimate the MBC. After 24 h incubation, the plates were examined where the lowest concentration with no observable growth on subculture was regarded as the MBC.

### In vivo antibacterial assessment and burn healing evaluation

Burn wound animal model was used for assessment of burn healing effect of the two propolis concentrations. 10 albino mice (weight 30–35 g), 8 weeks old, were used. Mice were separately kept in clean polyethylene cages under standard experimental conditions at 23±2°C. Mice were anaesthetized using isoflurane inhalation anesthesia device then shaved on the back using an electric clipper followed by a shaving cream. Ethanol (70% v/v) was used to disinfect the shaved area. Burn wounds were induced using a 10 mm diameter cylindrical metal rod, pre-heated over an open flame for 20 seconds then pressed for 10 seconds on the dorsal mouse skin surface under anesthesia, leading to the formation of a burn wound of diameter 10 mm. Finally, a total of 4 burn sites were generated on the dorsal skin of each mouse. All animal work was performed in agreement with the guidelines stated in the Guide for the Care and Use of Laboratory Animals and was approved by the Ethics Committee for Experimental, Clinical and Chemical Studies at Faculty of Pharmacy, The British University in Egypt (Serial number: EX-2204). At the end of in vivo burn healing evaluation experiment mice were humanely euthanized with CO_2_ inhalation according to institutional guidelines then dorsal skin samples were collected then subjected to histopathological analysis. After skin autopsy euthanized mice were frozen till incineration.

#### Bacterial infection induction

Bacterial infection of the induced burn wounds was done by inoculation of 1.5 x 10^7^ colony forming unit (CFU) of bacteria. Using sterile cotton swab, 1 mL bacteria suspension was inoculated evenly by direct rubbing onto the fresh wound.

#### Treatments

Each mouse received four treatments: one treatment per burn wound position. The first burn position was treated with propolis 100%, the second position was treated with propolis 50%, the third position was treated with normal saline acting as a negative control (N), and finally the fourth position was treated with a commercial burn healing cream (silver sulfadiazine) as a positive control (P). All treatments were applied topically by rubbing on the respective mouse position once daily using a sterile cotton swab and continued daily until apparent skin healing in any of the positions. All mice were fed on commercial pellet and water *ad libitum* through the study.

#### Measurement of burn area

Burn healing evaluation was estimated by measuring the decrease in the induced original burn diameter. A digital caliper was used to measure the burn diameter (mm) daily before treatment application. Recorded measurements were saved for more analysis.

#### Assessment of bacterial load of the infected burns

Swabs from the surface of burn positions were collected daily before the application of treatments. Sterile cotton swabs were rubbed over the total burn area; especially where the degree of burn is maximum; for 10 seconds with slight pressure. For estimating the bacterial load in each position; serial dilutions of homogenized samples in 1 mL sterile saline were plated on sterile agar media. Plates were incubated aerobically at 37°C for 24 h. Subsequently, counting of bacterial colonies was done by visual inspection. Colony forming unit (CFU) was used to determine the total bacterial count on each plate.

### Skin irritation test

Skin irritation test was done using rabbits after removal of fur on a section of the back. Propolis 100% and 50% were administered on the shaved skin then kept in contact for 4h. Skin irritation grade was scored for erythema, edema formation, eschar and corrosive effect. Observations continued for 24 h. Rabbits were housed for use in further research after a washout period.

### Skin sensitization test

Skin sensitization test was done using guinea pigs after removal of fur on a section of the back. Propolis 100% and 50% were administered daily to the skin for 14 days. Two weeks after the final administration, the guinea pigs were challenged by application of propolis 100% and 50%, and the appearance of erythematous reaction was assessed. Guinea pigs were housed for use in further research after a washout period.

### Histopathological analysis

Autopsy dorsal skin samples were collected from mice after observing apparent skin healing. A total of 40 skin samples were collected: four from each mouse demonstrating the locations of treatments with a total of 10 samples for each treatment. The Collected skin samples were fixed in 10% formalin solution for 24 h. Samples were washed with tap water followed by dehydration using serial dilutions of alcohol (methyl alcohol, ethyl alcohol and absolute ethyl alcohol). Skin samples were cleared in Xylene then embedded in paraffin at 56°C in hot air oven for 24 h. Paraffin bees wax tissue blocks were prepared for sectioning at 4 microns thickness by rotary LEITZ microtome. The obtained tissue sections were collected on glass slides, de-paraffinized and stained by hematoxylin & eosin stain (H&E) for observation using the light electric microscope [22]. Digital photomicrographs were taken, and the burns were assessed for the degree of acanthosis, formation and regeneration of new blood capillaries.

### In vitro antioxidant activity of propolis hydroalcoholic extract

Different techniques have been assessed to evaluate the antioxidant power of the hydroalcoholic extract of propolis. The spectrophotometric techniques applied in this study adopted the principle of electron transfer as well as reduction of the colored oxidant as DPPH, ABTS and FRAP. In DPPH and ABTS assays, change in color is due to the reduction power of the antioxidant sample whereas in FRAP, the change in color is due to the change of ferric ion to ferrous by the influence of the antioxidant sample. Implementation of different antioxidant assays would be effective in confirmation of the results [23].

### Propolis hydroalcoholic extract and trolox standard preparations

Propolis extract was prepared in ethanol at serial dilution (25–100 μg/mL), (5–25 μg/mL) and 0.8 mg/mL concentration. Trolox standard (20 μg/mL in methanol) was prepared in methanol in dilutions ranged from 1.25–6.25 μg/mL, 2.5–8.75 μg/mL and 25–3000 μM in the antioxidant assays DPPH, ABTS and FRAP, respectively. The procedures have been applied as previously mentioned in detail in previous literature [24]. A microplate reader FluoStar Omega has been used for data analysis and IC_50_ was calculated *via* GraphPad Prism 6 for DPPH and ABTS assays. The antioxidant activity of FRAP assay has been expressed in trolox equivalents (TE). All the results expressed as mean ± standard deviation of triplet measurements.

### Cell culture for anti-inflammatory activity of propolis hydroalcoholic extract

Murine macrophage RAW 264.7 cell line was cultured at 37°C in incubator with 5% CO_2_. The cells were kept in high-glucose DMEM containing 1% Pen-Strep (100 units/mL penicillin and 100 μg/mL streptomycin) and 10% heat inactivated fetal bovine serum (FBS). For MTT and Griess assay, the cells were seeded at density of 2×10^5^ in 96-well plates or at a density of 2×10^6^ cells/well in 6-well plate for RNA extraction for 2 h. RAW 264.7 cells were stimulated by LPS (10 ng/mL) and murine interferon-γ (10 U/mL) for 24 h with or without propolis. DMSO (0.1% v/v) was used as vehicle control.

### Cell viability

The colorimetric MTT assay was done to determine the nontoxic concentration of propolis as previously described [25]. RAW 264.7 macrophages were exposed for 24 h to propolis at increasing concentrations of (3.125 to 400 μM) alone or with LPS/ IFN-γ (10 ng/mL/10 U/mL). After 24 h, a new serum-free medium with 1 mg/mL MTT was added. The formed formazan crystals were dissolved with isopropanol and the OD was measured at 540 nm using Nano SPECTROstar microplate reader (BMG LABTECH, Ortenberg, Germany). Cell viability was calculated as the percentage of viable macrophages relative to the control.

### Nitrite assay

The Griess method was used to determine nitrite concentration as an indicator of NO production as previously described [26]. Cells were seeded for 2 h, activated by LPS/ IFN-γ (10 ng/mL/10 U/mL), and incubated with different concentrations of propolis (3.125 to 400 μM). Supernatant medium (150 μL) was diluted with deionized water (130 μL) and Griess reagent (20 μL) using Nano SPECTROstar microplate reader, the OD was measured at 550 nm. Nitrite concentration was calculated with reference to the standard sodium nitrite curve.

### Quantitative RT-PCR

RAW 264.7 macrophages were seeded at a density of 2×10^6^ in a 6 well plate for 2 h. Then activated by 10 ng/mL LPS plus 10 U/mL IFN-γ and incubated with different concentrations of propolis (3.125 to 400 μM). Total RNA was isolated using QiAzol according to the manufacturer’s instructions. RevertAid First Strand cDNA Synthesis kit was used to synthesize cDNA from 1 μg of total RNA. Relative expressions of mRNA (TLR4, TNF-α and IL-6) were carried on ABI 7500 real-time PCR system (Applied Biosystems) with SYBR green PCR master mix. NCBI primer design tool (https://www.ncbi.nlm.nih.gov/tools/primer-blast/) was used for primer design and Thermo Fisher for synthesis **Table 1**. The relative expression of the target genes was compared to the untreated cells, normalized by GAPDH level, using the 2−ΔΔCT method [27]. The conditions used for RT-PCR mRNA expression: 10 min at 95°C and 40 cycles of 95°C for 15 s and 60°C for 1 min.

**Table 1:** Primers used for qPCR analyses.

Primer sequence	Target Gene
**5’-**CTTTGTCAAGCTCATTTCCTGG- 3**’**	GAPDH-F
**5’-**TCTTGCTCAGTGTCCTTGC- 3**’**	GAPDH-R
**5’-**GATGCTACCAAACTGGATATAATCAG-3**’**	IL6-F
**5’-**CTCTGAAGGACTCTGGCTTTG-3**’**	IL6-R
**5’-**TTCAGAACTTCAGTGGCTGG-3**’**	TLR4-F
**5’-**TGTTAGTCCAGAGAAACTTCCTG-3**’**	TLR4-R
**5’-**GAACTCCAGGCGGTGCCTAT-3**’**	TNFa-F
**5’-**TGAGAGGGAGGCCATTTGGG-3**’**	TNFa-R

### Western blot

RAW 264.7 cells were seeded into 6-well plates (2 × 10^6^ cells/well) for 2 h. Then activated by 10 ng/mL LPS plus 10 U/mL IFN-γ and incubated with two concentrations of propolis (50 and 100 μg/mL) for 24 h. Indomethacin (IM, 0.25 mM) was used as an anti-inflammatory positive control.

RAW 264.7 cells were washed using cold PBS and scrapped in cold lysis buffer [RIPA buffer containing protease inhibitor cocktail-100 μl/well]. Cell lysates were incubated on ice for 15 min with occasional vortex every 5 min, then centrifuged at 10000 ×g for 10 min (4°C). BCA kit (Thermofisher Scientific, USA) was used for protein quantification. Samples (20 μg total proteins) were resolved using polyacrylamide gel electrophoresis on a 10% acrylamide/bis acrylamide gel using OmniPAGE TETRAD system (Cleaver scientific, UK) at 150 Volts for 1.5 h. Resolved proteins were electro-transferred onto nitrocellulose membrane (100 Volts for 70 min). Membranes were blocked using skim milk (5% w/v in Tris buffer saline Tween 20, TBST) for 1 h at room temperature. Membranes were probed overnight (4°C) with a rabbit iNOS antibody (Biomatik, Canada) and β-actin (Thermofisher Scientific, USA) under gentle shaking. Probed membranes were washed with TBST 3 times, 5 min each with shaking. Horseradish peroxidase-conjugated secondary antibodies were added to the membranes then incubated with shaking for 1 h at room temperature. For washing TBST (3 × 5 min) was used, and the protein bands were visualized using enzyme chemiluminescence kit (Clarity, Bio-Rad, USA).

### Statistical analysis

Our results are presented as mean ± SE of the performed experiments. For western blot, the intensity of each band was measured by using ImageJ software. Comparisons are made with ANOVA followed by Dunnett’s multiple comparison test; *, P < 0.05, compared with control. GraphPad Prism Software (Inc. San Diego, CA, version 5.0) was used.

## Results and discussion

### Identification of secondary metabolites using UHPLC/MS-PDA

In a previous study by Arafa, et. al. 2018, UPLC-PDA-HRMS identifcation of the major flavonoids in the same propolis sample was depiceted. Only the major flavonoids *viz*. pinobanksin, chrysin, pinocembrin and galangin have been identified through their mass fragmentation pattern [28]. Hereby in this study, a full chemical profiling of the whole propolis sample was performed using UHPLC/MS-PDA to understand the influence of all phytochemicals on the antibacterial, antioxidant and anti-inflammatory activities of propolis.

**Fig 1:**
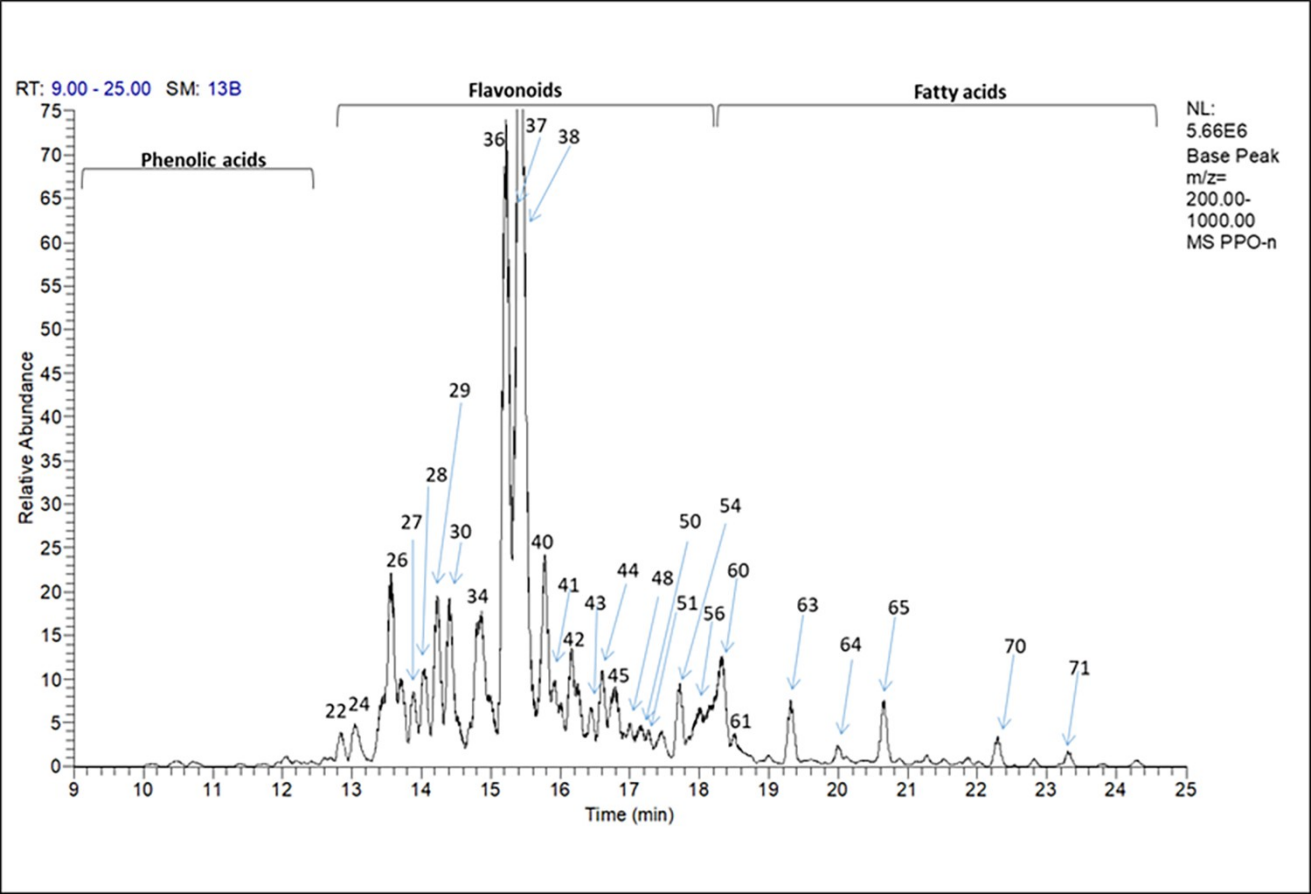
UHPLC/MS-PDA total ion chromatogram of propolis hydroalcoholic extract. The identities, retention time values (*R*_*t*_), UV and mass data are listed in **Table 2**. Numbers represent peak number of each metabolite.

The interpretation of the peaks was carried out on the negative mode since it is highly sensitive for the identification of the phenolic compounds [29]. **Table 2** summarizes the compounds identified in the propolis sample. The identification depends on the detection of the [M-H]^-^ and further analysis of the MS^2^ fragments. The identified compounds were compared to the MS/MS fragmentation of literature data. A total of 71 compounds were identified including sugars, phenolic acids and their derivatives, flavonoids “free, methylated and ester forms” and fatty acids. The total ion chromatogram is illustrated in **(Fig 1).**

**Table 2:** Identified propolis hydroalcoholic extract secondary metabolites by UHPLC/MS-PDA in the negative ionization mode.

Peak	Rt. (min)	UV	(M-H)	Formula-H	MS-MS	Error	ID	Class
**P-1**	1.09	254, 366	179.0558	C_6_H_11_O_6_	131, 161	-1.906	Hexose	Sugar
**P-2**	1.35	363, 370	221.0661	C_8_H_13_O_7_	161	-2.515	Di-O-methyl-hexuronic acid	Sugar acid
**P-3**	1.51	274	169.0141	C_7_H_5_O_5_	125	-1.045	Gallic acid	Phenolic acid
**P-4**	2.10	279	131.0348	C_5_H_7_O_4_	87	-1.084	Deoxy-arabinolactone	Sugar
**P-5**	5.12	372	181.0489	C_9_H_9_O_4_	137	-0.167	Unknown acid	Acid
**P-6**	9.06	323	200.0091	C_11_H_4_O_4_	110	-11.934	unknown	_
**P-7**	10.24	319	193.0503	C_10_H_9_O_4_	149	-1.772	Ferulic acid	Phenolic acid
**P-8**	10.34	319	287.0537	C_15_H_11_O_6_	177	-8.47	unknown	_
**P-9**	10.45	319	315.0863	C_17_H_15_O_6_	161, 179, 271	-1.853	Caffeic acid derivative	Phenolic acid
**P-10**	10.87	298, 323	609.1472	C_27_H_29_O_16_	301, 464	-4.035	Rutin	Flavonol glycoside
**P-11**	10.90	298, 323	267.0849	C_13_H_15_O_6_	193	-9.403	1-O-Feruloyl-glycerol	Phenolic acid
**P-12**	10.91	244	165.0538	C_9_H_9_O_3_	121, 150	-11.86	unknown acid	Acid
**P-13**	11.28	280	445.1486	C_23_H_25_O_9_	255, 265, 341, 385	-4.146	Pinocembrin derivative	Flavanone
**P-14**	11.39	270, 319	247.0969	C_14_H_15_O_4_	147, 187, 203	-2.802	Dihydroxy-prenylcinnamic acid	Prenylated Phenolic acid
**P-15**	11.67	279, 319	457.1711 (503.18)	C_21_H_29_O_11_	293, 457	-1.061	unknown	_
**P-16**	11.90	279, 364	283.0613	C_16_H_11_O_5_	240, 268	0.294	Galangin-5-methyl ether	Flavonol
**P-17**	11.75	279	405.1181 (451.12)	C_20_H_21_O_9_	341	-2.408	Trihydroxy-oxododecanoic acid.	Fatty acid
**P-18**	12.14	279, 364	313.0716	C_17_H_13_O_6_	_	-0.643	3′,7-Dihydroxy-5′,6-dimethoxyisoflavone	Isoflavone
**P-19**	12.24	297, 322	301.0706	C_16_H_13_O_6_	165	-3.791	3’, 4’,6-Trihydroxy-7-methoxyflavanone	Flavanone
**P-20**	12.74	279, 364	301.0347	C_15_H_9_O_7_	151, 179	-2.245	Quercetin	Flavonol
**P-21**	12.77	323	463.1018	C_25_ H_19_ O_9_	309, 353	-3.682	Phenolic acid derivative	Phenolic acid
**P-22**	12.85	296	285.076	C_16_H_13_O_5_	139, 241, 253, 267	-3.216	Methyl Pinobanksin	Dihydroflavonol
**P-23**	13.00	273	229.0863	C_14_H_13_O_3_	_	-3.176	3-(2,3-Dihydro-2-isopropenyl-5-benzofuranyl)-2-propenoic acid	Benzofuran
**P-24**	13.04	277, 363	315.0502	C_16_H_11_O_7_	300	-2.526	Quercetin methyl ether	Flavonol
**P-25**	13.31	279, 323	427.1387	C_23_H_23_O_8_	135, 179, 247, 255, 273, 341	-2.741	Caffeic acid isoprenyl ester derivative	Phenolic acid
**P-26**	13.56	292	271.0606	C_15_H_11_O_5_	253	-2.386	Pinobanksin	Dihydroflavonol
**P-27**	13.88	269, 362	299.0555	C_16_H_11_O_6_	284	-2.011	Kampferol methyl ether	Flavonol
**P-28**	14.05	269, 362	329.066	C_17_H_13_O_7_	314	-2.115	Quercetin di methyl ether	Flavonol
**P-29**	14.22	290	435.1072	C_24_H_19_O_8_	135, 179, 255, 281	-2.99	Caffeoyl pinocembrin	Flavanone-Phenolic acid
**P-30**	14.44	266, 362	283.0606	C_16_H_11_O_5_	239, 268	-2.179	Galangin -3-methyl ether	Flavonol
**P-31**	14.65	274	447.1074	C_25_H_19_O_8_	337, 403	-2.641	Flavonoid derivative	Flavonoid
**P-32**	14.65	274	407.1124	C_23_H_19_O_7_	297	-3.086	5-hydroxy, 3,7, dimethoxy flavone derivative	Flavone
**P-33**	14.73	273	419.1129	C_24_H_19_O_7_	255, 375	-2.782	*p*-coumaroyl trihydroxyflavone	Flavone-phenolic acid
**P-34**	14.83	269, 362	329.0658	C_17_ H_13_O_7_	299, 314	-2.662	Quercetin di methyl ether isomer	Flavonol
**P-35**	14.95	279, 289	449.1231	C_25_H_21_O_8_	339, 417, 434	-2.473	unknown	**_**
**P-36**	15.22	269, 312	253.0502	C_15_H_9_O_4_	209	-1.628	Chrysin	Flavone
**P-37**	15.32	290	255.0658	C_15_H_11_O_4_	151, 213	-1.773	Pinocembrin	Flavanone
**P-38**	15.42	267, 360	269.0452	C_15_H_9_O_5_	241, 227	-1.289	Galangin	Flavonol
**P-39**	15.68	271, 368	431.0761 (477.1179)	C_24_H_15_O_8_	162, 269, 367	-2.553	Galangin derivative	Flavonol
P-40	15.78	267, 290, 341	417.0973 (463.1393)	C_24_H_17_O_7_	162, 255, 353	-1.573	Pinocembrin derivative	Flavanone
P-41	15.88	272, 368	415.0818 (461.1231)	C_24_H_15_O_7_	269, 373, 387	-1.219	Galangin derivative	Flavonol
**P-42**	16.16	279, 319	389.1027	C_23_H_17_O_6_	295, 374	-1.032	unknown	_
**P-43**	16.38	277, 291	327.0866	C_18_H_15_O_6_	_	-2.45	5,7-Dihydroxy-3-propanoyloxyflavanone. (3-*O*-Propanoylpinobanksin)	Flavanone
**P-44**	16.59	276, 319	389.1026	C_23_H_17_O_6_	295, 374	-1.032	unknown	_
**P-45**	16.79	287	405.1335	C_24_H_21_O_6_	255, 281, 295, 390	-2.176	Pinocembrin derivative	Flavanone
**P-46**	16.94	277	403.1179	C_24_H_19_O_6_	255, 267, 388	-2.112	7-Hydroxy-5-methoxyflavone derivative	Flavone
**P-47**	17.04	287	433.1282	C_25_H_21_O_7_	403, 418	-2.554	8-Cinnamoyl-3,3′,5′,7-tetrahydroxy-5-methoxyflavan.	Flavan
**P-48**	17.06	289	341.1021	C_19_H_17_O_6_	253	-2.702	Pinobanksin butyrate [(3-O-(2-Methylpropanoyl) pinobanksin)]	Dihydroflavonol
**P-49**	17.16	288	451.1399	C_25_H_23_O_8_	_	0.042	Propolisbenzofuran A	Benzofuran
**P-50**	17.33	270, 296sh	387.0865	C_23_H_15_O_6_	255, 343, 369	-2.458	Pinocembrin derivative	Flavone
**P-51**	17.57	279	455.2429	C_27_H_35_O_6_	393, 411, 437	-2.245	Unknown	_
**P-52**	17.60	277, 360	399.0868 (445.1281)	C_24_H_15_O_6_	269	-1.557	Galangin derivative	Flavonol
**P-53**	17.69	291	355.1179	C_20_H_19_O_6_	271, 253	-2.314	Pinobanksin valerate [(3-O-(2-methylbutanoyl) pinobanksin)]	Dihydroflavonol
**P-54**	17.88	289	385.1074 (403.0811)	C_24_H_17_O_5_	255	-2.043	Pinocembrin derivative	Flavanone
**P-55**	17.96	286	323.1284	C_20_H_19_O_4_	164, 219, 268, 281	-1.431	unknown	_
**P-56**	18.01	_	471.3465	C_30_H_47_O_4_	_	-3.083	3,7-Dihydroxycycloart-24-en-28-oic acid	Cycloartan- terpenoids
**P-57**	18.06	270	337.1075	C_20_H_17_O_5_	253, 282	-1.889	Chrysin derivative	Flavone
**P-58**	18.11	269	369.1123	C_24_H_17_O_4_	_	-2.417	6-Cinnamyl chrysin	Flavone-Phenolic acid
**P-59**	18.32	272, 297sh	385.107	C_24_H_17_O_5_	_	-3.082	unknown	_
**P-60**	18.50	_	297.2427	C_18_H_33_O_3_	171	-2.618	Hydroxy linoleic acid	Fatty acid
**P-61**	18.82	_	325.183	C_21_H_25_O_3_	325	6.249	Unknown sterol	sterol
**P-62**	19.32	_	343.2843	C_20_H_39_O_4_	283, 325	-3.067	Dihydroxy eicosenoic acid	Fatty acid
**P-63**	20.01	_	357.3001	C_21_H_41_O_4_	297, 339	-2.499	unknown	**_**
**P-64**	20.66	_	371.3155	C_22_H_43_O_4_	311, 353	-3.132	Dihydroxy docosahexenoic acid	Fatty acid
**P-65**	21.68	_	353.3049	C_22_H_41_O_3_	225	-3.534	Hydroxy-docosenoic acid	Fatty acid
**P-66**	21.71	_	313.2735	C_19_H_37_O_3_	142, 180	-4.304	Hydroxynonadecanoic acid	Fatty acid
**P-67**	21.80	_	399.3474	C_24_H_47_O_4_	339, 381	-1.36	Dihydroxytetracosanoic acid	Fatty acid
**P-68**	22.09	_	413.3243	C_24_H_45_O_5_	353	-7.035	Dimethoxy hydroxy-docosenoic acid	Fatty acid
**P-69**	22.28	_	327.2896	C_20_H_39_O_3_	267, 281	-2.806	Hydroxyeicosanoic acid	Fatty acid
**P-70**	22.77	_	341.3049	C_21_H_41_O_3_	253, 264, 326	-3.57	Methoxyeicosanoic acid.	Fatty acid
**P-71**	23.18	_	355.3209	C_22_H_43_O_3_	198, 232, 287	-2.388	Hydroxydocosanoic acid	Fatty acid

#### Phenolic acids

Few phenolic acids have been detected as gallic acid **P-3** [m/z 169.0147 (C_7_H_5_O_5_)^-^] **S1 Fig**; ferulic acid **P-7** [m/z 193.0503 (C_10_H_9_O_4_)^-^] with their UV_max_ at 274 and 319, respectively and common fragmentation of COO^-^ moiety loss (-44 Da) resulting in peaks (125 and 149 Da, respectively) **S2 Fig**. A Phenolic glycerol *viz*. 1-*O*-feruloyl glycerol **P-11** m/z 267.0849 (C_13_H_15_O_6_)^-^], UV_max_ at 298, 323 with major MS^2^ peak of ferulic acid (m/z 193) and a glycerol moiety of m/z 74 **S5 Fig.** In previous literatures, isolation and identification of different phenolic glycerides have been observed in poplar type propolis [30] as well as Chinese one [31]. A dihydroxy-prenyl cinnamic acid **P-14** [m/z 247.0969 (C_14_H_15_O_4_)^-^], UV_max_ 270, 319 with MS^2^ peaks of m/z 187 owing to the loss of carboxylic and hydroxy groups (-60 Da), m/z 203 due to loss of carboxylic group (COO^-^, 44 Da) and 147 m/z of cinnamic acid **S8 Fig**. Presence of derivatives of phenolic acids as caffeic acid isoprenyl ester derivative **P-25** [m/z 427.1387 (C_23_H_23_O_8_)^-^], UV_max_ 279, 323 whereas appearance of fragments as caffeic acid isoprenyl ester [m/z 247 (C_14_H_15_O_4_)^-^] and caffeic acid [m/z 179 (C_9_H_7_O_4_)^-^] **S16 Fig.** Other peaks as **P-9** and **P-21,** their MS^2^ fragmentation revealed the loss of (-44 Da, COO^-^ group) which represents loss of carboxylic group as shown in **Table 2**. **P-9**, a caffeic acid derivative [m/z 315.0863 (C_17_H_15_O_6_)^-^] **S3 Fig** showed a fragment at m/z 179.03 which stands for molecular formula (C_9_H_7_O_4_)^-^ of caffeic acid and fragment [m/z 271.10 (C_16_H_15_O_4_)^-^ which represents loss of COO^-^ group (-44 Da)]. **P-21**, is a phenolic acid derivative [m/z 463.1018 (C_25_H_19_O_9_)^-^] **S13 Fig** where a fragment at m/z 309.08 due to loss of COO^-^ moiety from m/z 353.07 (C_19_H_13_O_7_)^-^. The identified phenolic acids are commonly present in propolis obtained from different origins [32,33] except for gallic acid where this is the first report of its identification in such sample. A peak **P-12** [m/z 165 (C_9_H_9_O_3)_] was assigned as unknown acid due to loss of COO^-^ group (-44 Da) **S6 Fig**. Another peak **P-51** [m/z 455.2429 (C_27_H_35_O_6_)] was assigned to have an acid moiety due to the loss of (-44 Da, COO^-^ group) and m/z 60 which represents loss of both carboxylic and hydroxyl groups **S34 Fig.**

#### Flavonoids

Flavonoids are considered a major class of this propolis sample [34]. Different common classes have been previously identified as flavones of UV_max_ 270 *viz*, chrysin [28]; flavonols of UV_max_ 360 *viz*, galangin [28] and quercetin; flavanones and dihydroflavonols (UV_max_ 292) as pinocembrin and pinobanksin [28] besides flavans UV_max_ 287 as in **Table 2.** Flavonoids represented in this propolis sample are aglycones, esters, methylated flavonoids and glycoside derivatives. Peaks of flavonoid aglycones were identified as 3’, 7 dihydroxy-5’, 6’ di methoxy isoflavone **P-18** [m/z 313.0716 (C_17_H_13_O_6_)^-^] and 3’, 4’, 6 trihydroxy-7-methoxy flavanone **P-19** [m/z 301.0706 (C_16_H_13_O_6_)^-^] which has been previously isolated from Nepalese propolis [35] **S10 and S11 Figs** respectively. Peak **P-47** [m/z 433.1282 (C_25_H_21_O_7_)^-^] **S31 Fig** was identified as 8-Cinnamoyl-3,3′,5′,7-tetrahydroxy-5-methyoxyflavan whereas it has been isolated previously from Chinese propolis [36]. Common flavonoid aglycones in propolis as quercetin **P-20** [m/z 301.0347 (C_15_H_9_O_7_)^-^] **S12 Fig** [33]. Pinobanksin **P-26** [271.0606 (C_15_H_11_O_5_)^-^], chrysin **P-36** [m/z 253.0502 (C_15_H_9_O_4_)^-^] pinocembrin **P-37** [m/z 255.0658 (C_15_H_11_O_4_)^-^] and galangin **P-38** [m/z 269.0452 (C_15_H_9_O_5_)^-^] in which those four aglycones were identified and reported for the same propolis sample in previous literature [28] as well as in Jordanian propolis [37]. Flavonoid esters as 5, 7-dihydroxy-3-propanoyloxyflavanone commonly named 3-*O*-propanoyl pinobanksin **P-43** [m/z 327.0866 (C_18_H_15_O_6_)^-^] and [(3-*O*-(2-Methylpropanoyl) pinobanksin)] known as pinobanksin butyrate **P-48** [m/z 341.1021 (C_19_H_17_O_6_)^-^]**. S28 and S32 Figs**, respectively, were also detected and previously identified in Chilean propolis [38]; (3-*O*-(2-Methylbutanoyl) pinobanksin) known as Pinobanksin valerate **P-53** [m/z 355.1179 (C_20_H_19_O_6_)^-^] **S36 Fig** [33]. Methylated flavonoids as methyl pinobanksin **P-22** [m/z 285.0759 (C_16_H_13_O_5_)^-^] **S14 Fig**, quercetin methyl ether **P-24** [m/z 315.0502 (C_16_H_11_O_7_)^-^] **S15 Fig**, Kaempferol methyl ether **P-27** [m/z 299.0555 (C_16_H_11_O_6_)^-^] **S17 Fig**, quercetin di-methyl ether **P-28 S18 Fig** and **P-34** [m/z 329.0660 (C_17_H_13_O_7_)^-^], galangin-5-methyl ether [m/z 283.0606 (C_16_H_11_O_5_)^-^] **P-16 S9 Fig** and galangin-3-methyl ether **P-30** [m/z 283.0606 (C_16_H_11_O_5_)^-^] **S20 Fig**, where the identification and fragmentation of these methylated flavonoids **Table 2** agreed with previous literature [33].

Identification of hydrophilic flavonoid glycosides is considered unique in such sample due to the lipophilic nature of propolis, still previous identification of glycosides of quercetin and kaempferol derivatives was reported previously in [39] which have been classified in such work as uncommon flavonoid glycosides in Portuguese propolis. In this propolis sample, identification of rutin **P-10** [m/z 609.1472 (C_27_H_29_O_16_)^-^] as detection of peak at m/z 301.03 which represents quercetin aglycone and loss of (-308 Da) which represents the sugar moiety, another peak at m/z 464.43 which stands for loss of deoxy-sugar rhamnose (-145 Da) **S4 Fig [37,40]**.

Derivatives of both galangin and pinocembrin flavonoids have been predicted in peaks **P-39** [m/z 431.0761 (C_24_H_15_O_8_)^-^], **P-41** [m/z 415.0818 (C_24_H_15_O_7_)^-^] and **P-52** [m/z 399.0868 (C_24_H_15_O_6_)^-^]. Galangin aglycon peak at m/z 269.04 (C_15_H_9_O_5_)^-^ was identified where loss of (-162 Da) in peak **P-39 S24 Fig** and (-146 Da) in **P-41 S26 Fig,** as well as loss of (-130 Da) in peak **P-52 S35 Fig**, respectively, have been observed. Pinocembrin flavonoid derivatives identified in peaks **P-40** [m/z 417.0973 (C_24_H_17_O_7_)^-^] **S25 Fig**, **P-50** [m/z 387.0865 (C_23_H_15_O_6_)^-^] **S33 Fig** and **P-54** [m/z 403.0811 (C_24_H_17_O_5_)^-^] **S37 Fig** with pinocembrin aglycone [m/z 255.07 (C_15_H_11_O_4_)] with loss of (-162 Da), (-132 Da) and (-130 Da) for **P-40**, **P-50** and **P-54**, respectively. Another pinocembrin derivatives could be detected for **P-13** [m/z 445.1486 (C_23_H_25_O_9_)^-^] **S7 Fig** and **P-45** [m/z 405.1335 (C_24_H_21_O_6_)^-^] where a peak at m/z 255.06 (C_15_H_11_O_4_)^-^ of pinocembrin was observed **S29 Fig.** A peak **P-46** [m/z 403.1179 (C_24_H_19_O_6_)^-^ could be a hydroxy-methoxy flavone derivative where a peak of pinocembrin m/z 255.07 (C_15_H_11_O_4_)^-^ was predicted in addition to another peak at m/z 388.10 (C_23_H_16_O_6_)^-^ due to loss of methyl group (-15 Da) **S30 Fig.** Flavonoid derivative **P-31** [m/z 447.1074 (C_25_H_19_O_8_)^-^] **S21 Fig** could be structurally related to **P-32** [m/z 407.1124 (C_23_H_19_O_7_)^-^] **S22 Fig** which could be a hydroxy dimethoxyflavone derivative as both showed the loss of C_6_H_6_O_2_ (-110 Da). It is worth mentioning that the isolation of hydroxy methoxy flavones have been carried out in a previous study from Argentinean propolis [41]. **P-57** [m/z 337.1075 (C_20_H_17_O_5_)^-^] **S39 Fig** could be chrysin derivative as a significant peak appeared at m/z 253.05 (C_15_H_9_O_4_)^-^ which stands for chrysin.

Another class of flavonoids with phenolic acids within their structures was identified in this propolis sample as caffeoyl pinocembrin **P-29** [m/z 435.1072 (C_24_H_19_O_8_)^-^] **S19 Fig** as detection of peaks at m/z 255.07 (C_15_H_11_O_4_)^-^ and 179.04 (C_9_H_7_O_4_)^-^ which present pinocembrin and caffeic acid moieties, respectively. *p*-coumaroyl trihydroxy-flavone **P-33** [m/z 419.1129 (C_24_H_19_O_7_)^-^] **S23 Fig** as a peak at m/z 255.07 (C_15_H_11_O_4_)^-^ was identified as trihydroxy-flavone [M-163]^-^ where loss of (-163 Da) occurred which presents *p*-coumaroyl moiety and m/z 375.12 which presents [M-44]^-^ due to loss of (-44 Da) of COO^-^ moiety of *p*-coumaroyl part. A 6-cinnamyl chrysin **P-58** [m/z 369.1123 (C_24_H_17_O_4_)^-^] **S40 Fig** may be predicted where it has been isolated before from a Chinese propolis sample [42]. A propolis Benzofuran compound **P-49** [m/z 451.1399 (C_25_H_23_O_8_)^-^] could be identified as it has been previously isolated from Brazilian propolis [43].

#### Fatty acids

Fatty acid class was identified at the end of the run. Identified fatty acids were **P-60** [m/z 297.2427 (C_18_H_33_O_3_)^-^], hydroxy linoleic acid; **P-62** [m/z 343.2843 (C_20_H_39_O_4_)^-^], dihydroxy eicosenoic acid; **P-64** [m/z 371.3155 (C_22_H_43_O_4_)^-^]; dihydroxy docosahexenoic acid; **P-65** [m/z 353.3049 (C_22_H_41_O_3_)^-^], hydroxy-docosenoic acid; **P-66** [m/z 313.2735 (C_19_H_37_O_3_)-], hydroxynonadecanoic acid; **P-67** [m/z 399.3474 (C_24_H_47_O_4_)^-^], dihydroxytetracosanoic acid; **P-68** [m/z 413.3243 (C_24_H_45_O_5_)^-^], dimethoxy hydroxy-docosenoic acid; **P-69** [m/z 327.2896 (C_20_H_39_O_3_)^-^], hydroxyeicosanoic acid; **P-70** [m/z 341.3049 (C_21_H_41_O_3_)^-^], Methoxyeicosanoic acid; **P-71** [m/z 355.3209 (C_22_H_43_O_3_)^-^], hydroxydocosanoic acid and an unknown sterol **P-61** [m/z 325.183 (C_21_H_25_O_3_)^-^]. Another peak could be detected **P-56** [m/z 471.3465 (C_30_H_47_O_4_)^-^] **S38 Fig** as 3,7-Dihydroxycycloart-24-en-28-oic acid where it has been isolated previously from Cretan propolis [44] also a group of cycloartane terpenes have been previously isolated from propolis obtained from Myanmar [45].

From the analysis of the propolis sample using UHPLC/MS-PDA, it could be predicted that it is a poplar type of propolis due to the presence of major specific flavonoids *viz*. chrysin, galangin, pinocembrin and pinobanksin with their derivatives [46,47]. The antibacterial activity of propolis could be related mainly to the enrichment of the sample with flavonoid class **Table 2** and **(Fig 1)**, in addition to the phenolic acids mainly caffeic acid and its derivatives [48]. In the next section, standardization of the hydroalcoholic extract of propolis would be crucial for quantification of its phenolic ingredients using HPLC-PDA.

### Standardization of phenolics in propolis extract using HPLC-PDA

**Fig 2:**
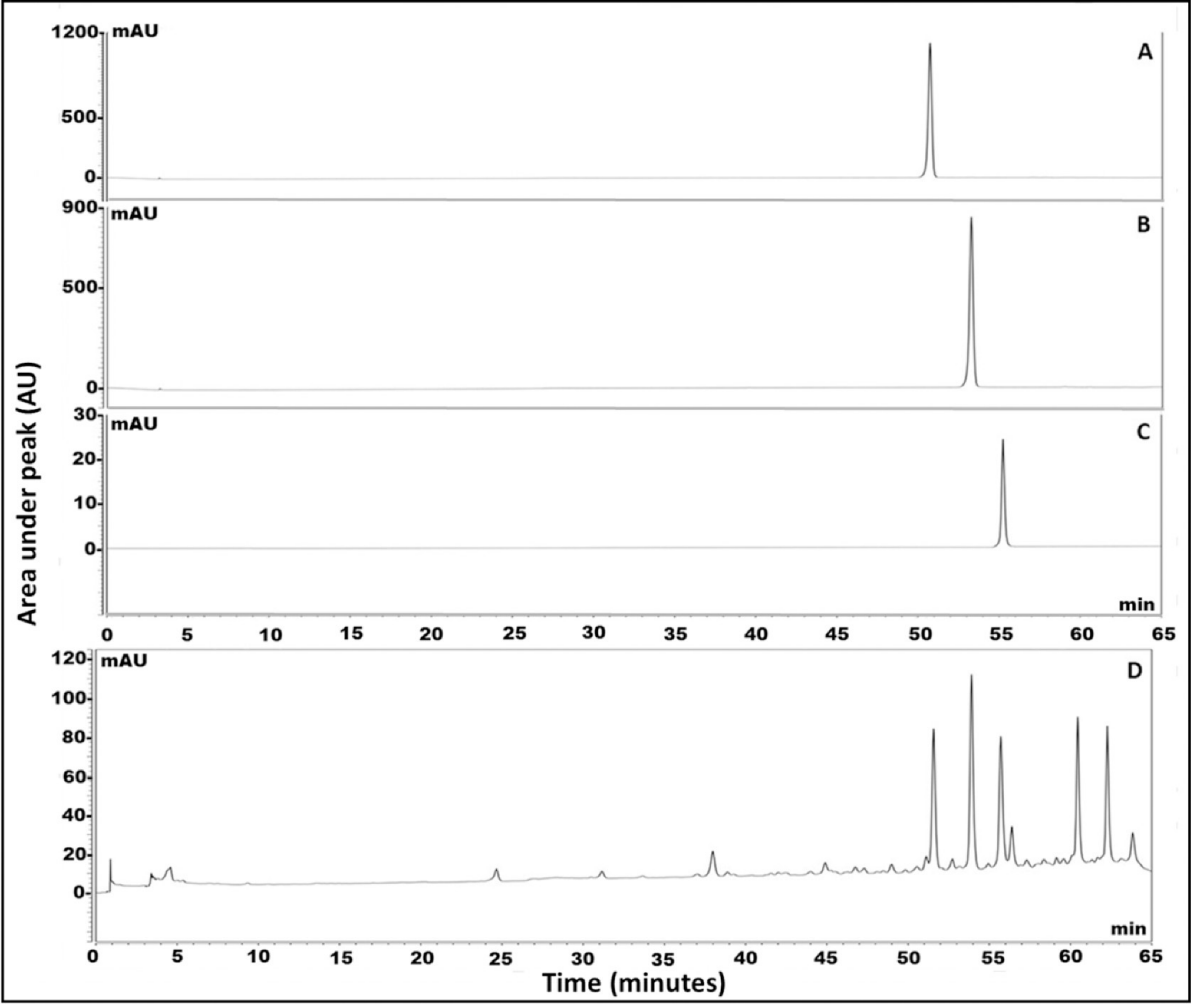
HPLC chromatogram of authentics. (A) pinocembrin, (B) chrysin, (C) galangin and (D) total propolis hydroalcoholic extract.

Standardization of the hydroalcoholic extract of propolis versus major flavonoids identified in the UHPLC/MS-PDA chromatographic run **Table 2** have been assessed using HPLC-PDA. Identification of the major flavonoids at retention time (*R*_*t*_.) *viz*. pinocembrin, chrysin and galangin were 51.00, 53.30, and 55.00 min, respectively (**Fig 2).** The results showed the enrichment of propolis sample by chrysin, followed by pinocembrin then galangin recording 22.73±0.68, 21.58±0.84 and 14.26±0.70 mg/g propolis extract, respectively. It is worth to mention the enrichment of propolis alcoholic extract of this current study with high content of flavonoids when compared to other propolis of different geographical sources [49]. In the previous work by Bozkuş et al. 2021, the content of both chrysin and galangin were recorded 641.33 and 534.11 μg/25 mg of Turkish propolis extract, respectively that would enhance its biological activities [49].

### In vitro antimicrobial evaluation of hydroalcoholic extract of propolis

#### Minimum Inhibitory concentration (MIC) and Minimum Bactericidal concentration (MBC)

The MIC of propolis could not be identified due to its dark color and turbidity where visualization would have been inaccurate in the prepared test tubes. This problem was resolved by going forward to identification of MBC through plating all dilutions on Mueller hinton agar to identify the bactericidal concentration of propolis. Plate number (5) showed that the MBC of propolis was 0.2 mg/mL.

#### Disc diffusion susceptibility test

**Fig 3:**
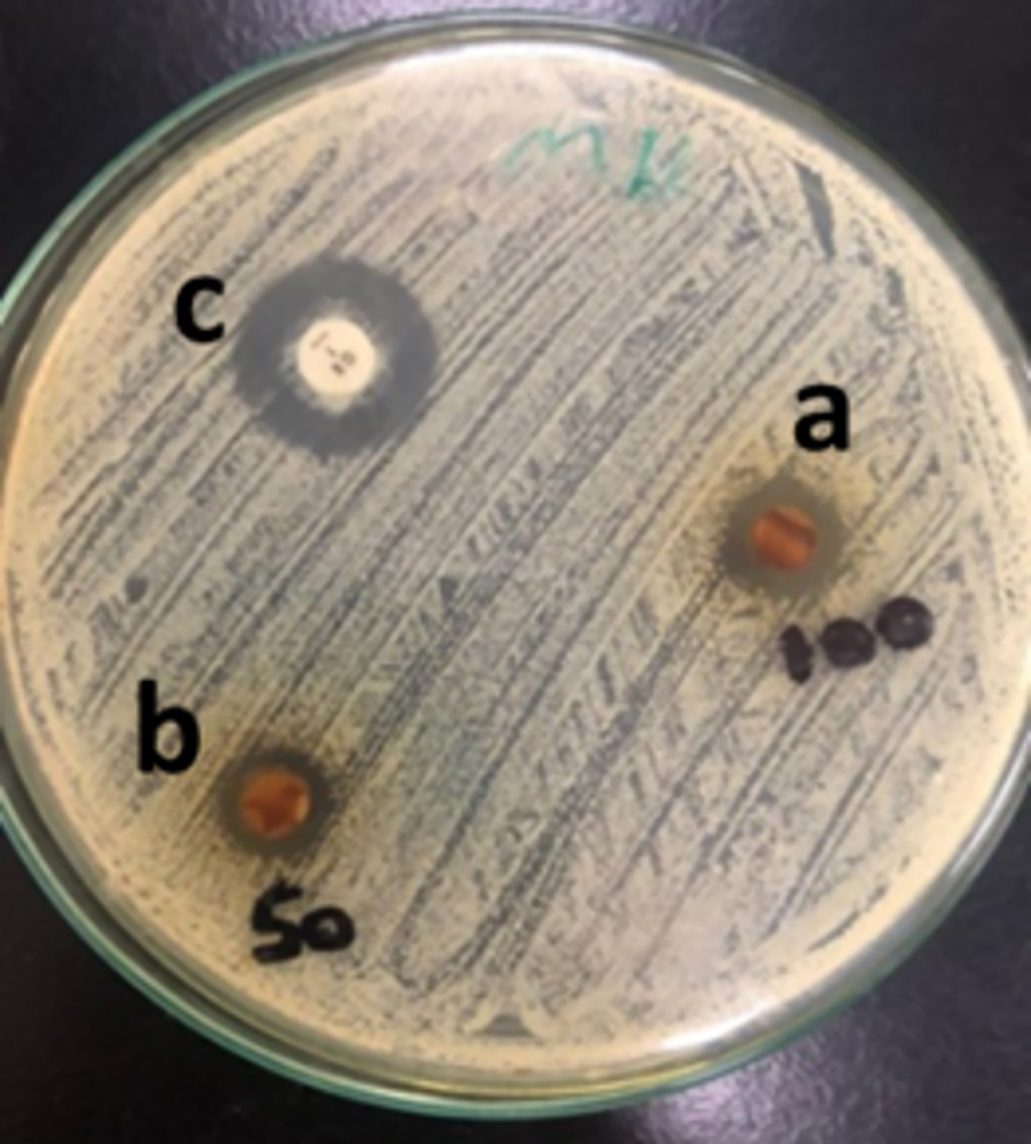
Disc Diffusion susceptibility test of the propolis against *Staphylococcus aureus*. a: 100% propolis; b: 50% propolis c: Control; Oxacillin antibiotic disc.

This test was to assess the antibacterial effect of the two propolis concentrations. Propolis 100%, 50% and oxacillin showed diameters of zones of inhibition measuring 14, 11 and 18 mm respectively (**Fig 3)** The test showed that *Staphylococcus aureus* was sensitive to 100% propolis and showed intermediate susceptibility to 50% propolis compared to the standard antibiotic. This shows the concentration dependent activity of propolis that is due to the higher concentration of constituents in the 100% concentration.

Generally, it appears that the antibacterial activity of propolis is greater against Gram-positive than Gram-negative bacteria. This could be attributed to the species-specific structure of the outer membrane of the Gram-negative bacteria and the production of hydrolytic enzymes which break down the active ingredients of propolis [50,51]. Other studies demonstrated that the broad spectrum biological properties of propolis, including antimicrobial activities, anti-inflammatory, immunomodulatory, antioxidant and radical scavenging actions are mostly due to phenolic compounds, terpenes, caffeic, ferulic and coumaric acids, esters, and flavonoids [52–54].

### In vivo antibacterial evaluation and burn healing assessment

Burn wound mouse model was used to assess the antibacterial and burn healing properties of the tested two propolis concentrations. Both concentrations were applied until complete healing was observed.

#### Burn healing assessment

**Fig 4:**
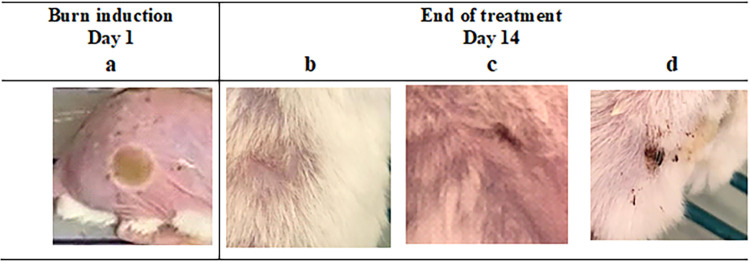
Burn wound on mouse dorsal skin before and after treatments application. a: Mouse burn (diameter 10 mm) before treatment application at day 1. b, c, d: Mouse burn after end of treatment at day 14, of 100% propolis, 50% propolis and silver sulfadiazine respectively.

The main criterion for indication of ongoing healing was measurement of burn wound diameter, where contraction of burn border expressed reduction of the original diameter of burn. The readings were documented daily through the duration of treatment. The positions treated on all mice with both concentrations of propolis; 100% and 50%, demonstrated on-going burn border contraction along the duration of treatment. The end of the treatment period was decided by visual observation of complete skin healing which was reached by 100% propolis by day 14 (**Fig 4) and Table 3**. It was found that the antibacterial activity of propolis was due to bacterial cell membrane damage and cell lysis [55]. Furthermore, it has the ability to inhibit cell division, protein synthesis and bacterial motility by affecting RNA-polymerase [56].

**Table 3:** Burn wound diameter percent reduction of the applied treatments at day 14.

**Mean percent reduction ± SD**	**Treatment**			
	Propolis 100%	Propolis 50%	Cream (positive control)	Saline (negative control)
	98.9 ± 1	89 ± 3.1	80.5 ± 4.8	65 ± 4.2

#### Assessment of bacterial load of the infected burns

Swabs were collected daily from the burn sites to assess the antibacterial activity of the tested propolis compared to the commercial product; silver sulfadiazine cream; as a positive control. All swabs from all positions showed reduction in bacterial load with time. By day 7 no bacteria were found in burn sites treated with both propolis concentrations. While burn sites treated with positive control continued to show bacterial growth till day 9, finally superficial bacterial growth was detected in the saline treated burn sites till the end of the treatment period (day 14) **Table 4**.

Percent reduction = $\frac{A-B}{A}\;x\;100$

Where:

A: Bacterial count before treatment

B: Bacterial count after treatment

**Table 4:** Bacterial load percent reduction of the applied treatments at day 7.

**Mean percent reduction ± SD**	**Treatment**			
	Propolis 100%	Propolis 50%	Cream (positive control)	Saline (negative control)
	99 ± 1.1	99 ± 1.1	85 ± 3.7	45 ± 4

### Skin irritation and sensitization tests

The possible effect of propolis to produce skin irritation and sensitization was tested and it was found that it did not yield any undesirable side effects when applied on the skin either once (irritation in rabbits) or repeatedly (sensitization in guinea pigs) for both concentrations.

### Histopathological analysis

**Fig 5:**
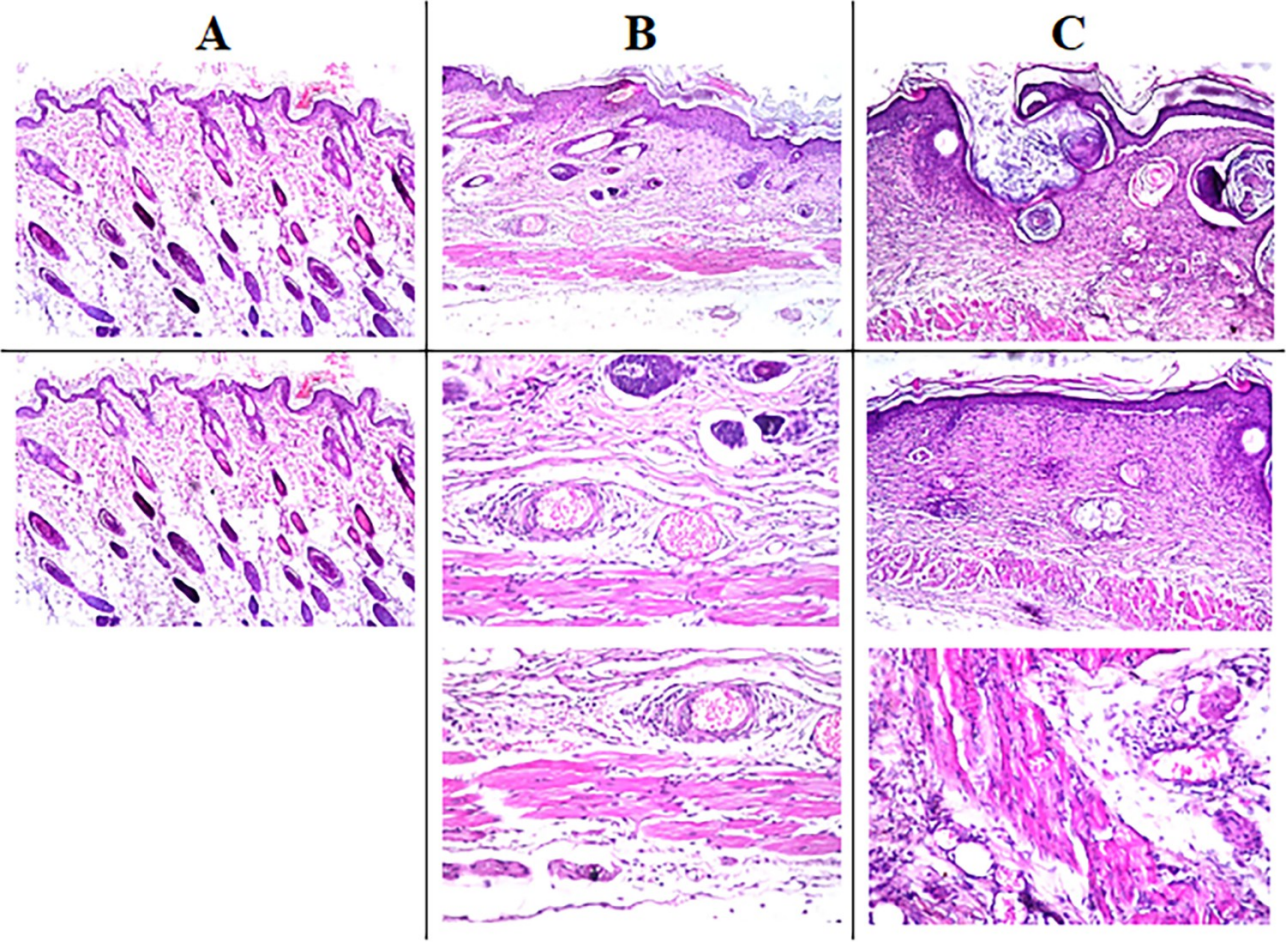
Representative photomicrographs of tissue sections collected at day 14 after burn stained with H&E. **A:** Skin of mice treated with 100% propolis showing few inflammatory cells and oedema in between the hair follicles at the dermal layer underneath the epidermis and between the dermis and muscular layer. (16x mag.) **B:** Skin of mice treated with 50% propolis showing acanthosis in the epidermis with underlying fibrosis and inflammatory cells in the dermis and musculature accompanied by oedema with dilation of blood vessels (16x mag.; 40x mag.; 40x mag.). **C:** Skin of mice treated with positive control showing invagination of the epidermal layer in the dermis with fibrosis and inflammatory cells infiltration accompanied by oedema and congested blood vessels. (16x mag.; 16x mag.; 40x mag.).

Skin autopsy samples treated with 100% propolis showed few inflammatory cells infiltration and oedema between the hair follicles at the dermis underneath the epidermal layer (**Fig 5A)**. The tissue between the deep dermis and musculature showed few focal inflammatory cells infiltration. The skin positions treated with 50% propolis showed focal thickening acanthosis in the epidermis associated with fibrosis and inflammatory cells infiltration in the underlying dermis (**Fig 5B)**. The deep layer of the dermis showed oedema with dilatation in the blood vessels, while the deep layer of the dermis and musculature had inflammatory cells infiltration. Perivascular inflammatory cells infiltration was detected surrounding the dilated blood vessels between the dermis and musculature. On the other hand, the positions treated with silver sulfadiazine showed focal invagination of the epidermis into the dermis, associated with fibrosis with inflammatory cells infiltration in the dermis underneath the epidermis. Oedema infiltrated with inflammatory cells was observed in between the dermis and musculature (**Fig 5C)**. The tissue between the dermis and the adjacent musculature also showed oedema, congested blood vessels and inflammatory cells infiltration. Sever suppuration in the subcutaneous tissue was found in the group treated with saline, while this was not seen with other groups. The severity of histopathological alteration in skin autopsy of different experimental groups has been summarized in **Table 5.**

**Table 5:** The severity of histopathological alteration in skin autopsy of different experimental groups.

Histopathological alterations	Groups*/Score				
	1	2	3	4	
Acanthosis in the epidermis	-	++	++	-	
Inflammatory reaction in subcutaneous tissue	+	++	++	+++	
Suppuration in subcutaneous tissue	-	-	-	+++	
Fibrosis and edema	-	++	+++	+++	

*Group 1: Treated with 100% propolis, Group 2: Treated with 50% propolis, Group3: Treated with silver sulfadiazine, Group 4: Treated with saline. Score: +++ Sever, ++ Moderate, + Mild,—Nil.

Gregory et al., 2002 compared healing activity of Brazilian propolis cream with silver sulfadiazine on patients who suffer minor second-degree burns, where propolis showed to be more effective than silver sulfadiazine in terms of inflammation reduction [57].

In another study, it was shown that 5% propolis ointment administered twice daily reduced the wound size of diabetic foot ulcer during a four-week long observation healing process [58]. In another randomized trial, foot ulcer areas in diabetic patients were also significantly reduced with the use of aqueous propolis in liquid form. This reduction, accompanied with improved healing rate, was detected regardless of whether the patient administered an antibiotic or not. Furthermore, wound fluids in the group treated with propolis showed decreased bacterial count [59].

### Antioxidant activity in vitro using different techniques (DPPH, ABTS and FRAP)

**Fig 6:**
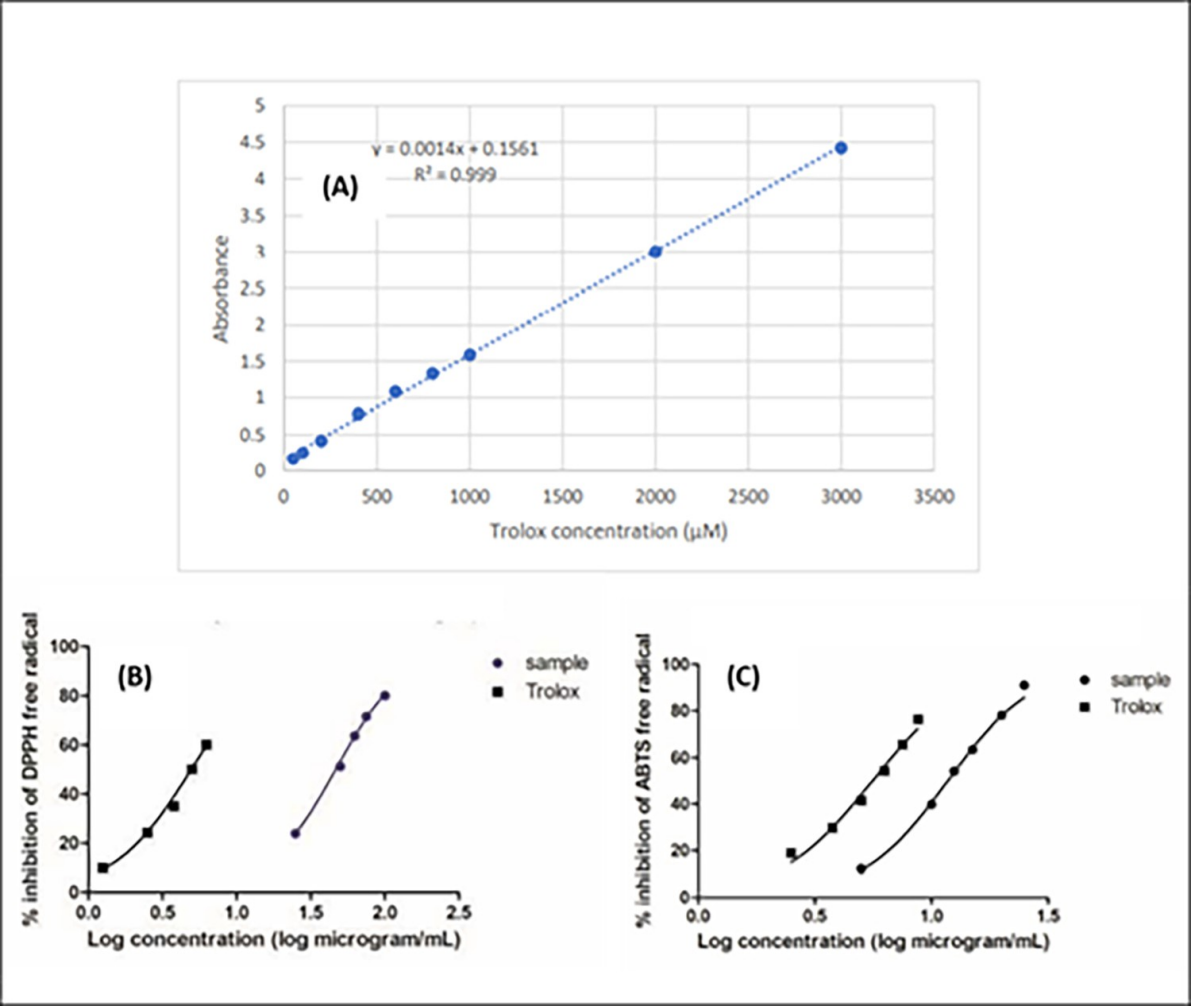
Antioxidant activity of propolis extract using different assays. (A): Trolox calibration curve used in FRAP assay (B): DPPH assay, (C): ABTS assay.

The antioxidant activity of the hydroalcoholic extract of propolis has been assessed *via* three different assays *viz*. DPPH, ABTS and FRAP. All the antioxidant assays results revealed the promising antioxidant effect of the propolis sample used in this study. The DPPH IC_50_ of propolis extract was 46.52±1.25 μg/mL (standard trolox 4.89±0.26 μg/mL) where the results matched those previously published in [60] of Taiwanese propolis from different districts. The ABTS assay revealed IC_50_ result 11.74±0.26 μg/mL (standard trolox 5.58±0.10 μg/mL) whereas the FARP assay recorded 445.29±29.9 μM TE/mg of propolis extract. The results are summarized in (**Fig 6).**

### Concentration‑dependent effect of propolis on the viability of RAW 264.7 macrophages

**Fig 7:**
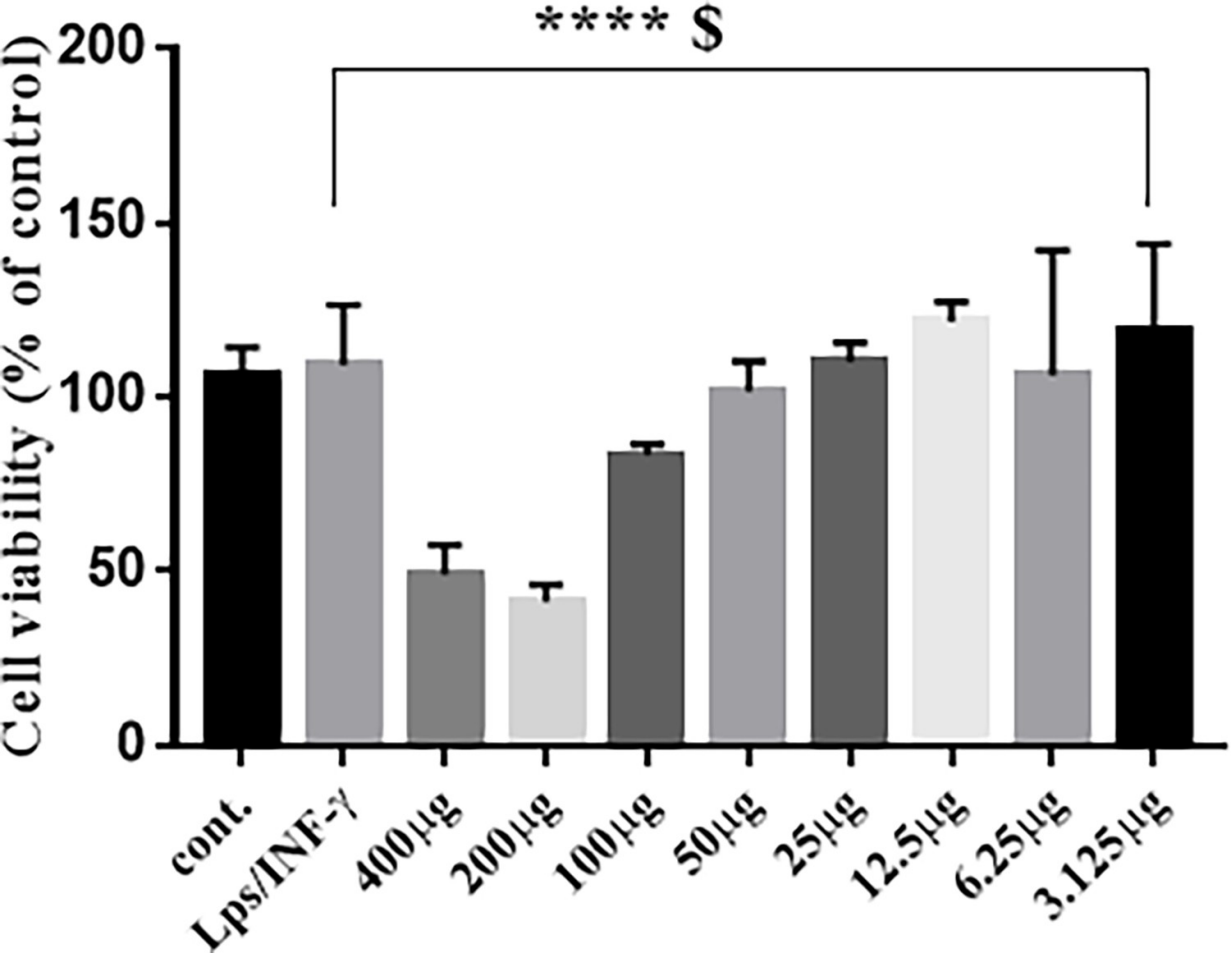
Effects of propolis on cell viability. RAW 264.7 macrophages were exposed for 24 h to propolis at increasing concentrations of (3.125 to 400 μM) alone or with LPS (10 ng/mL) plus IFN-γ (10 U/mL). Cell cytotoxicity was determined using MTT assay. Data are expressed as a percentage of untreated control (which is set at 100%) ± S.E. (n = 4).

MTT assay was used to determine the nontoxic concentration of propolis, RAW 264.7 cells were treated for 24 h with increasing concentrations of propolis (3.125 to 400 μg) alone or with LPS (10 ng/mL) plus IFN-γ (10 U/mL). (**Fig 7)** shows that exposure to propolis inhibited cell viability in a concentration dependent manner. When cells were stimulated with LPS (10 ng/mL) plus IFN-γ (10 U/mL) alone, no change in cell viability was observed. The 50 μg concentration did not significantly affect cell viability compared to LPS/ IFN-γ control and accordingly was used in all downstream experiments.

### Concentration‑dependent effects of propolis on nitrite formation in RAW 264.7 macrophages

**Fig 8:**
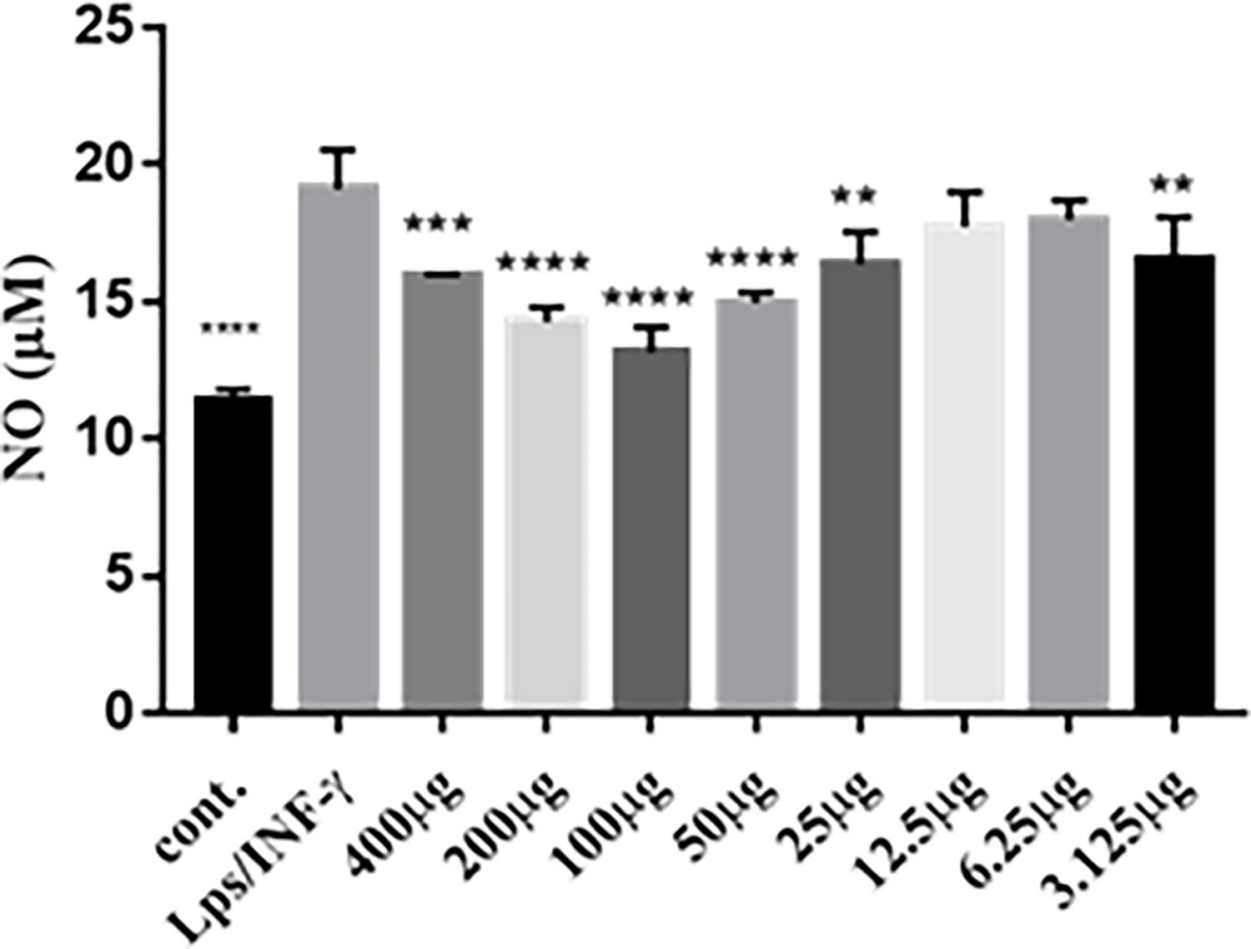
Concentration-dependent effects of LPS and IFN-γ on nitrite production in RAW 264.7 macrophages. RAW 264.7 macrophages were exposed to LPS/IFN-γ (10 ng/mL/10 U/mL) and different propolis concentrations for 24 h. Data are expressed as mean ± S.E. (n  =  8).

Nitrite concentrations were measured using the Griess assay. RAW 264.7 cells were subjected for 24 h to propolis (3.125 to 400 μg) alone or with LPS /IFN-γ (10 ng/mL/10 U/mL). (**Fig 8)** shows that LPS/IFN-γ have increased nitrite level by 160% compared to control. Cells exposed to propolis (concentration 100–6.25 μg) and LPS/IFN-γ, did not affect cell viability and also inhibited nitrite production in concentration dependent manner.

### Effect of propolis on TNF-α, TLR4 and IL-6 mRNA expression levels in LPS/IFN‑γ‑stimulated RAW 264.7 macrophages

**Fig 9:**
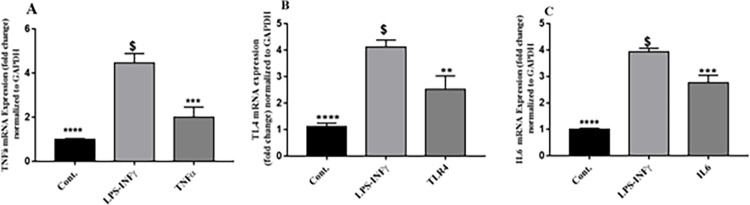
Effect of Total propolis extract on TNF-α, TLR4 and IL-6 mRNA in LPS/IFN-γ-mediated RAW 264.7 macrophages. RAW 264.7 cells were treated for 24 h with propolis (50 μg) in the presence of LPS (10 ng/mL) plus IFN-γ (10 U/mL). TNF-α (A), TLR4 (B) and IL-6 (C) mRNA levels were quantified using qRT-PCR and were normalized to GAPDH. Data are expressed as mean ± S.E. (n = 3).

TNF-α, TLR4 and IL-6 mRNA expression levels were estimated by real-time PCR. In (**Fig 9)**, cells treated with LPS/IFN-γ (10 ng/mL-10 U/mL) for 6 h significantly induced TNF-α, TLR4 and IL-6 expression by approximately 400, 450 and 400%, respectively, compared to untreated control. RAW cells activated with LPS/IFN-γ and co-treated with 50 μg propolis for 24 h showed reduction in mRNA expression of TNF-α, TLR4 and IL-6 by 55, 40, 39% respectively.

In the current study, the effect of propolis on TLR4 signaling pathway was explored through gene expression studies of key inflammatory intermediaries as TLR4, TNF-α, IL-6 and iNOS. Upon activation of RAW 264.7 macrophages with LPS/IFN-γ, a significant increase in these mediators was detected. Propolis effectively attenuated LPS/IFN-γ induction of RAW 264.7 macrophage through TLR4 pathway by downregulating the expression of TLR4, TNF-α, IL-6 and iNOS at the transcriptional level and post-transcriptional level for iNOS. Furthermore, upon LPS/IFN-γ activation of RAW 264.7 macrophage, a significant increase in iNOS protein level and nitrite was detected as previously proved by Fresta et al. [61]. The significant overexpression of TLR4, IL-6 and TNF-α mRNA in LPS/IFN-γ activated RAW 264.7 macrophage (**Fig 9)**, confirmed the relation between LPS/TLR4 signal transduction and proinflammatory cytokine stimulation from macrophages [62]. These results are in agreement with other studies which show that propolis suppresses prostaglandin and leukotriene generation by inhibiting the expression and activities of cyclooxygenases (COX-1 and COX-2) and lipoxygenases (LOX), retarding the gene expression of inducible nitric oxide synthase (iNOS), blocking tumor necrosis factor-α (TNF-α)-mediated NF-қB activation and reducing immune response in T cells. Chrysin, pinocembrin and galangin counted as selective inhibitors of NF-қB activation as previously mentioned in previous literatures, which may provide the molecular basis for their anti-inflammatory activity [63–65].

### Effects of propolis on iNOS protein levels inLPS/IFN‑γ‑stimulated RAW 264.7 macrophages

**Fig 10:**
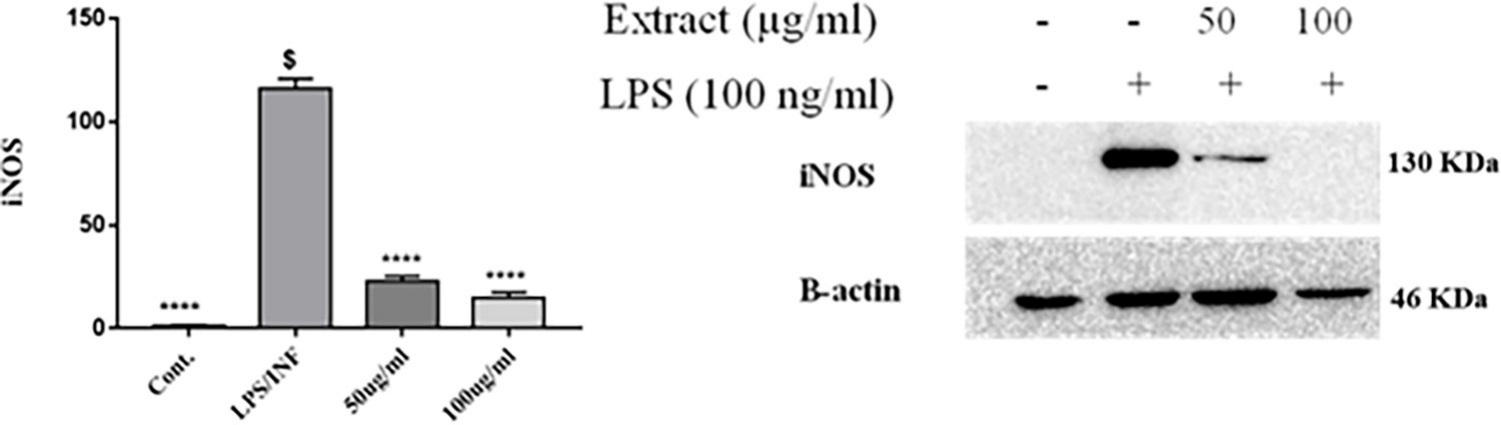
Concentration-dependent effect of propolis on iNOS protein expression on LPS and IFN-γ induced RAW 264.7 macrophages. RAW 264.7 macrophages were exposed to LPS (10 ng/mL) in the presence of IFN-γ (10 U/mL) for 24 h. RAW264.7 cells pretreated with 50 and 100 μg propolis. Anti-iNOS monoclonal antibodies were used for iNOS protein detection and actin was used as a loading control.

iNOS protein levels in LPS/IFN‑γ‑stimulated RAW 264.7 macrophages were evaluated by western blot. As illustrated in (**Figs 10 and S41)**, RAW 264.7 macrophages treated with LPS/IFN-γ (10 ng/mL–10 U/mL) shows an increase in the expression level of iNOS protein approximately by 1500% compared to the untreated cells. Upon treatment with propolis 50 and 100 μg, significant decrease in iNOS protein expression by 80 and 87%, respectively, compared to LPS/IFN-γ treated control.

## Conclusion

Burns are a global public health problem, accounting for an estimated 180 000 deaths annually. Despite a variety of burn treatment products there is still no consensus regarding better performance. Propolis; a natural product; enriched with a myriad of natural phytochemicals through which possess therapeutic potential in the treatment of infected burns including inflammatory ones.

In this current study, propolis proved to be a potent natural antibacterial agent that aid in burn healing. The antibacterial action against *Staphylococcus aureus* was tested in vitro; using Disc Diffusion susceptibility test and in vivo; using burn-induced infected mouse model. Propolis showed in vitro concentration dependent antibacterial activity and in vivo highest burn healing rate compared to a commercial product as shown by burn diameter reduction and histopathological analysis with no signs of skin irritation in rabbits, nor sensitization in guinea pigs.

In addition to the antibacterial and burn healing properties of propolis, this study unveiled its antioxidant and anti-inflammatory actions in vitro. Our results showed that propolis successfully modulates TLR4 signaling activation in RAW 264.7 macrophages by inhibiting LPS/IFN-γ-induced NO, iNOS, TNF-α, and IL-6 gene expression. Our data proves the anti-inflammatory potential of propolis at the transcriptional and posttranscriptional levels, and thus imply that propolis has a potential immunomodulatory agent against LPS/IFN-γ-mediated inflammation. The chemical profiling of propolis was conducted through full mapping of the phytochemical constituents using UHPLC/MS-PDA as well as standardization of the major flavonoids identified in this propolis sample using HPLC-PDA to relate the propolis biological activity to its phytochemical constituents through full identification as well as quantification of the major flavonoids.

## Supporting information

S1 Fig ESI-MS/MS spectrum of P-3 in the negative ion mode. (PDF)

S2 Fig ESI-MS/MS spectrum of P-7 in the negative ion mode. (PDF)

S3 Fig ESI-MS/MS spectrum of P-9 in the negative ion mode. (PDF)

S4 Fig ESI-MS/MS spectrum of P-10 in the negative ion mode. (PDF)

S5 Fig ESI-MS/MS spectrum of P-11 in the negative ion mode. (PDF)

S6 Fig ESI-MS/MS spectrum of P-12 in the negative ion mode. (PDF)

S7 Fig ESI-MS/MS spectrum of P-13 in the negative ion mode. (PDF)

S8 Fig ESI-MS/MS spectrum of P-14 in the negative ion mode. (PDF)

S9 Fig ESI-MS/MS spectrum of P-16 in the negative ion mode. (PDF)

S10 Fig ESI-MS/MS spectrum of P-18 in the negative ion mode. (PDF)

S11 Fig ESI-MS/MS spectrum of P-19 in the negative ion mode. (PDF)

S12 Fig ESI-MS/MS spectrum of P-20 in the negative ion mode. (PDF)

S13 Fig ESI-MS/MS spectrum of P-21 in the negative ion mode. (PDF)

S14 Fig ESI-MS/MS spectrum of P-22 in the negative ion mode. (PDF)

S15 Fig ESI-MS/MS spectrum of P-24 in the negative ion mode. (PDF)

S16 Fig ESI-MS/MS spectrum of P-25 in the negative ion mode. (PDF)

S17 Fig ESI-MS/MS spectrum of P-27 in the negative ion mode. (PDF)

S18 Fig ESI-MS/MS spectrum of P-28 in the negative ion mode. (PDF)

S19 Fig ESI-MS/MS spectrum of P-29 in the negative ion mode. (PDF)

S20 Fig ESI-MS/MS spectrum of P-30 in the negative ion mode. (PDF)

S21 Fig ESI-MS/MS spectrum of P-31 in the negative ion mode. (PDF)

S22 Fig ESI-MS/MS spectrum of P-32 in the negative ion mode. (PDF)

S23 Fig ESI-MS/MS spectrum of P-33 in the negative ion mode. (PDF)

S24 Fig ESI-MS/MS spectrum of P-39 in the negative ion mode. (PDF)

S25 Fig ESI-MS/MS spectrum of P-40 in the negative ion mode. (PDF)

S26 Fig ESI-MS/MS spectrum of P-41 in the negative ion mode. (PDF)

S27 Fig ESI-MS/MS spectrum of P-42 in the negative ion mode. (PDF)

S28 Fig ESI-MS/MS spectrum of P-43 in the negative ion mode. (PDF)

S29 Fig ESI-MS/MS spectrum of P-45 in the negative ion mode. (PDF)

S30 Fig ESI-MS/MS spectrum of P-46 in the negative ion mode. (PDF)

S31 Fig ESI-MS/MS spectrum of P-47 in the negative ion mode. (PDF)

S32 Fig ESI-MS/MS spectrum of P-48 in the negative ion mode. (PDF)

S33 Fig ESI-MS/MS spectrum of P-50 in the negative ion mode. (PDF)

S34 Fig ESI-MS/MS spectrum of P-51 in the negative ion mode. (PDF)

S35 Fig ESI-MS/MS spectrum of P-52 in the negative ion mode. (PDF)

S36 Fig ESI-MS/MS spectrum of P-53 in the negative ion mode. (PDF)

S37 Fig ESI-MS/MS spectrum of P-54 in the negative ion mode. (PDF)

S38 Fig ESI-MS/MS spectrum of P-56 in the negative ion mode. (PDF)

S39 Fig ESI-MS/MS spectrum of P-57 in the negative ion mode. (PDF)

S40 Fig ESI-MS/MS spectrum of P-58 in the negative ion mode. (PDF)

S41 Fig Original uncropped western blot image. (PDF)

S1 File (DOCX)
