# Correction: In vitro and in vivo burn healing study of standardized propolis: Unveiling its antibacterial, antioxidant and anti-inflammatory actions in relation to its phytochemical profiling

**DOI:** 10.1371/journal.pone.0319204

**Published:** 2025-02-06

**Authors:** Dina M. El-Kersh, Rania F. Abou El-Ezz, Eman Ramadan, Reham F. El-kased

In the Author Contributions, Rania F. Abou El-Ezz should be listed as one of the persons who developed the methodology.

Moreover, the other affiliation of the third author is missing. Eman Ramadan is also affiliated with #4: Center for Drug Research and Development (CDRD), The British University in Egypt, Cairo, Egypt.
