# Supplementary figures and images for: In vitro and in vivo burn healing study of standardized propolis: Unveiling its antibacterial, antioxidant and anti-inflammatory actions in relation to its phytochemical profiling
Source: PLoS One. 2024 May 14;19(5):e0302795. doi: 10.1371/journal.pone.0302795 (PMC11093344; doi:10.1371/journal.pone.0302795)

PPO-n #386 RT: 1.51 AV: 1 NL: 7.30E3  
T: FTMS - c ESI d Full ms2 169.01 @cid35.00 [50.00-:

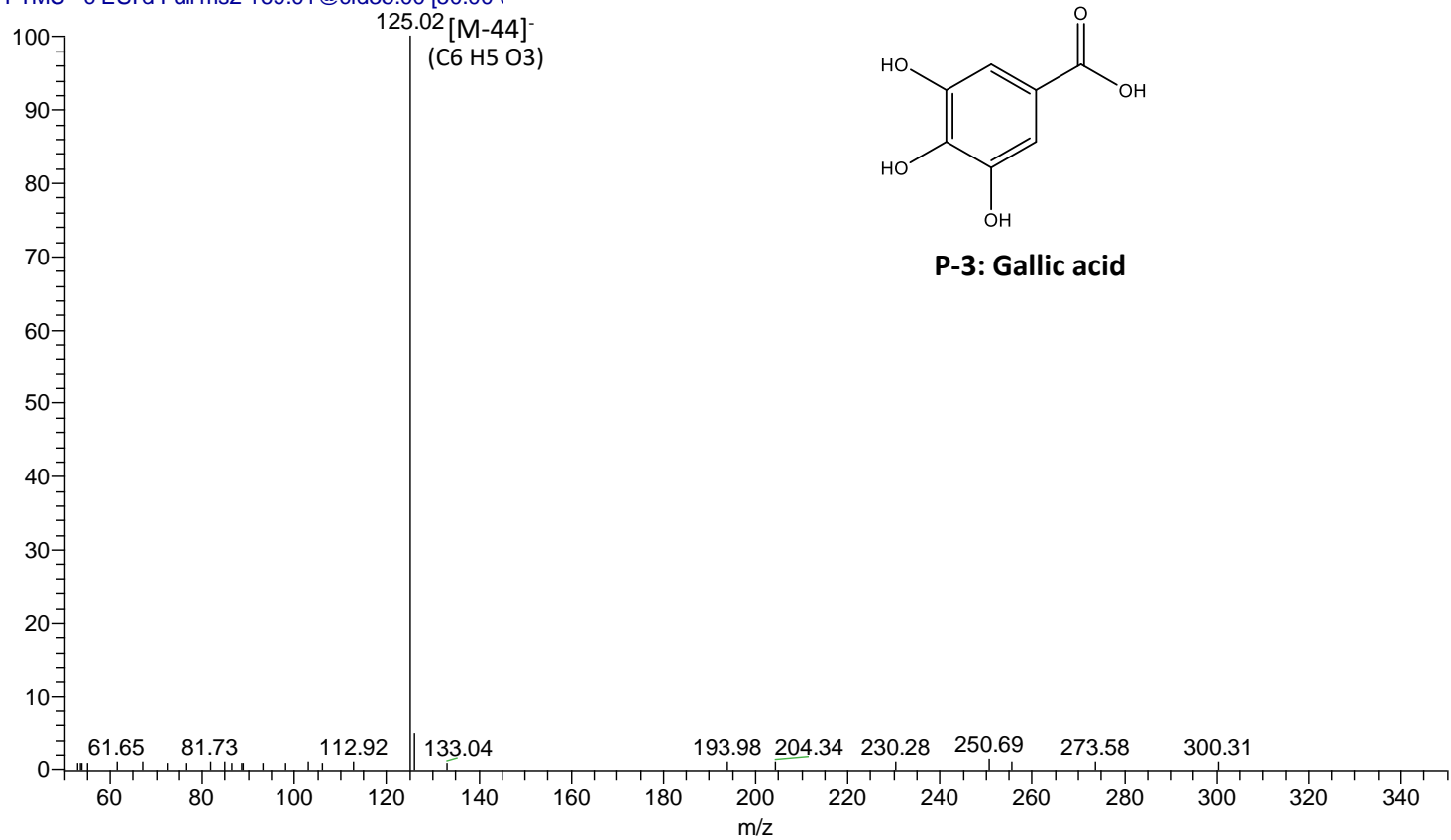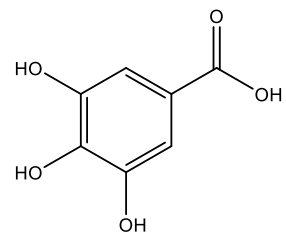

**P-3: Gallic acid**

**S1 Fig**

Supplement: S1 Fig — (PDF) [file pone.0302795.s001.pdf]

PPO-n #2652 RT: 10.24 AV: 1 NL: 1.28E2  
T: FTMS - c ESI d Full ms2 193.05 @cid35.00 [50.00-

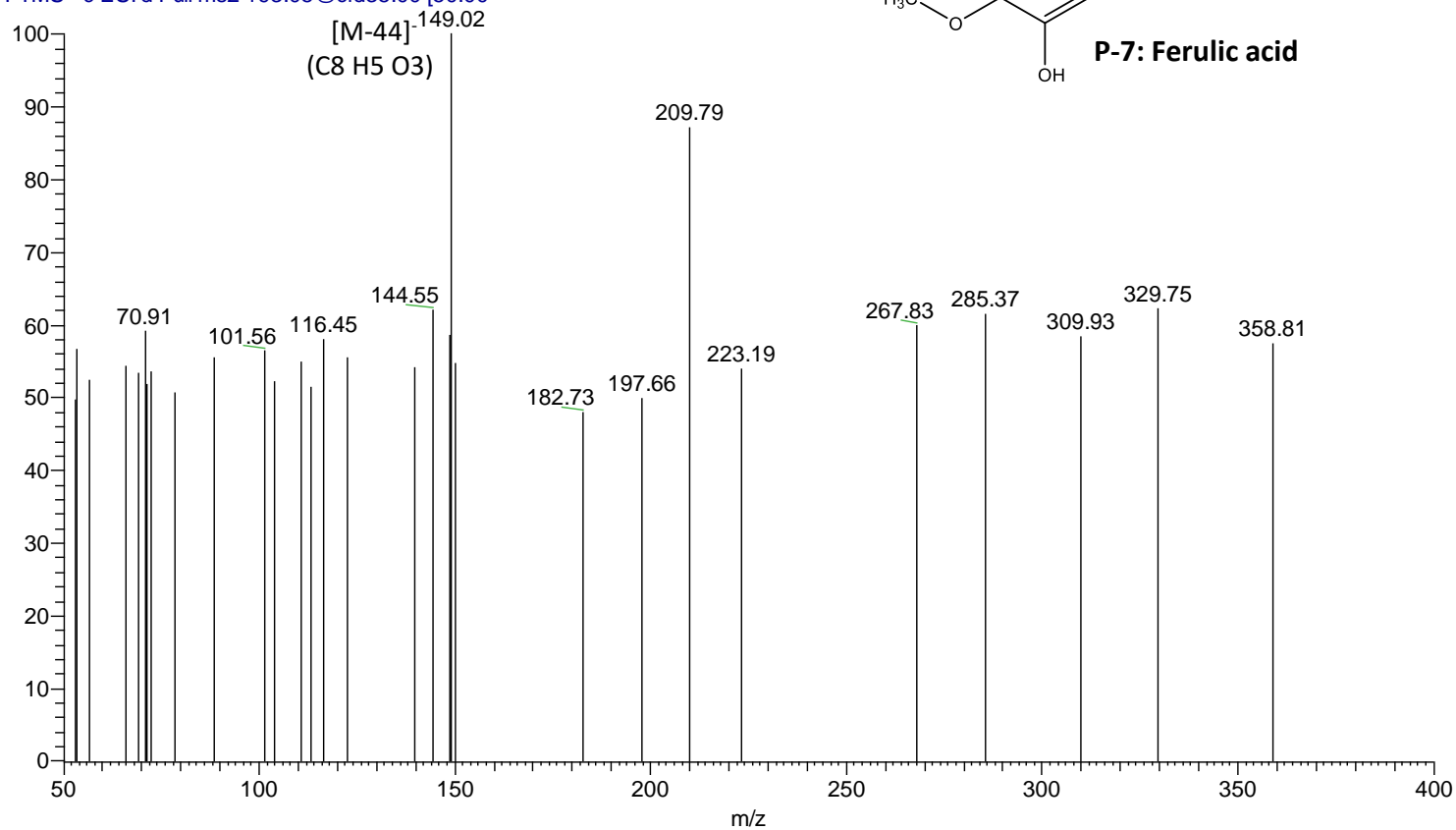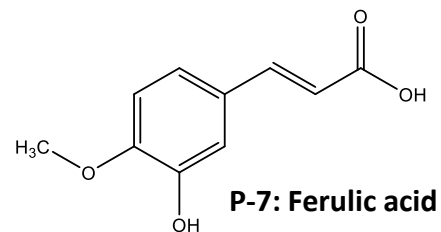

**S2 Fig**

Supplement: S2 Fig — (PDF) [file pone.0302795.s002.pdf]

PPO-n #2708 RT: 10.45 AV: 1 NL: 1.32E4  
T: FTMS - c ESI d Full ms2 315.09@cid35.00 [75.00-:

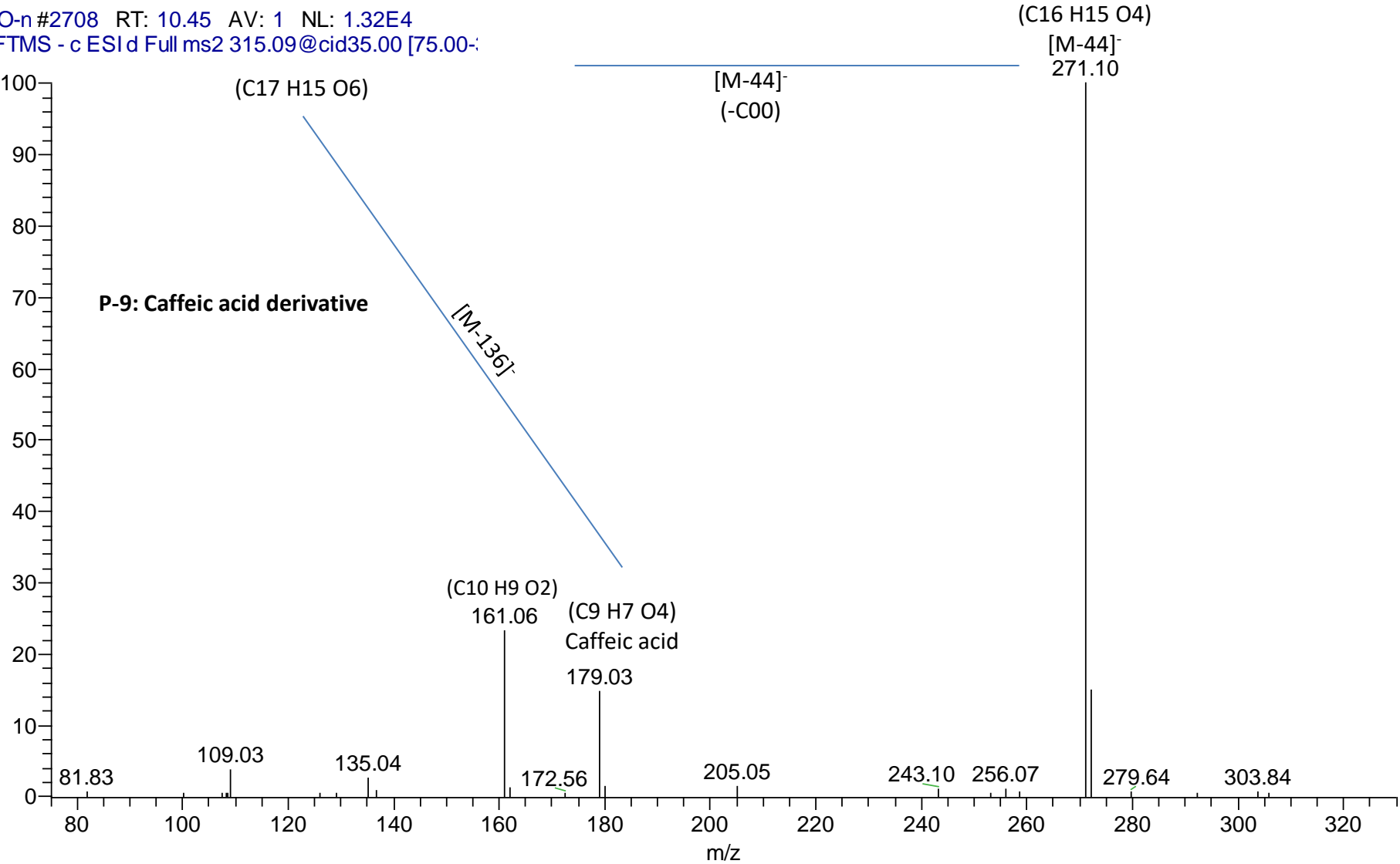

S3 Fig

Supplement: S3 Fig — (PDF) [file pone.0302795.s003.pdf]

PPO-n #2812 RT: 10.87 AV: 1 NL: 1.63E3  
T: FTMS - c ESI d Full ms2 609.14@cid35.00 [155.00]

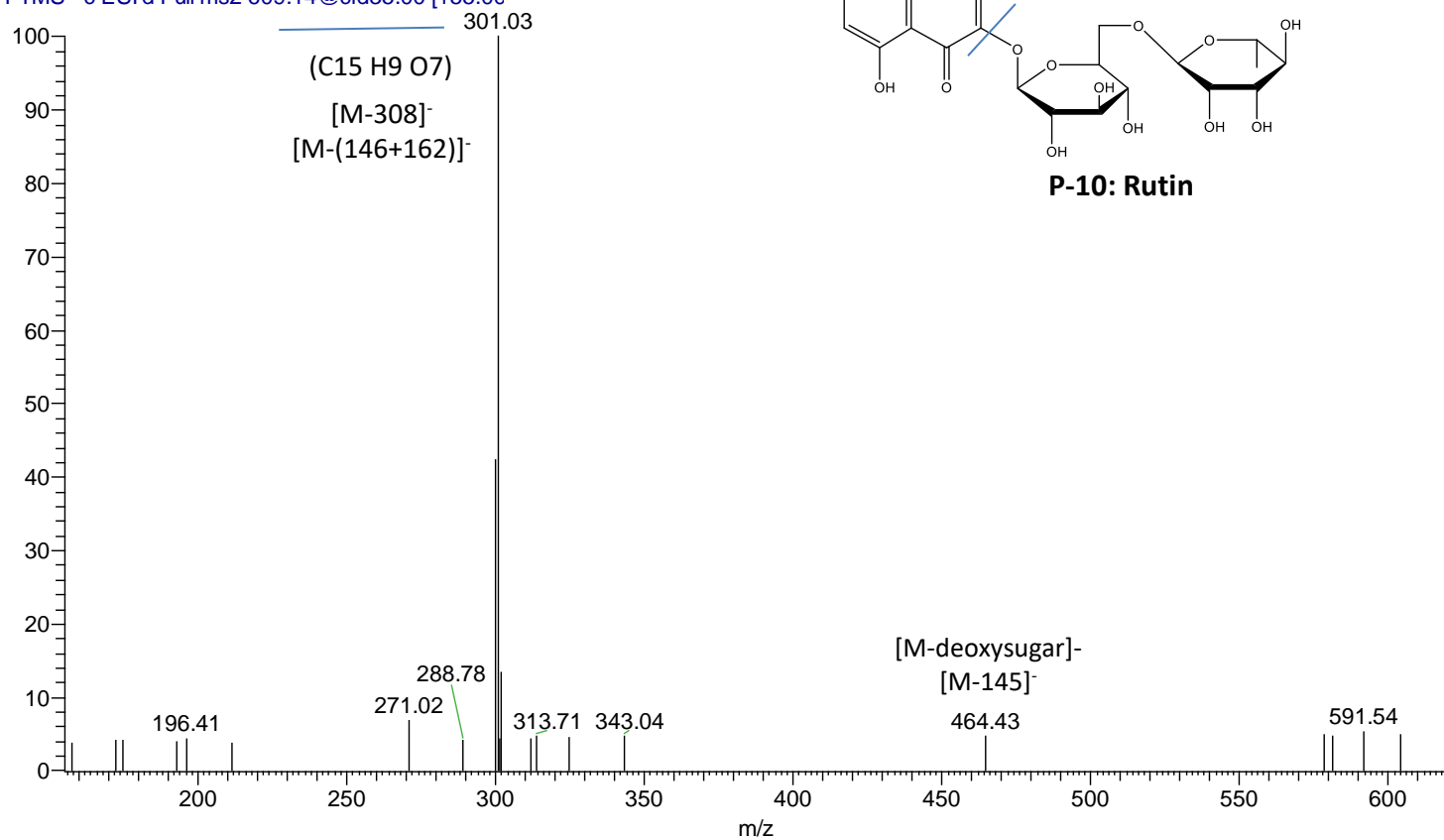

S4 Fig

Supplement: S4 Fig — (PDF) [file pone.0302795.s004.pdf]

PPO-n #2824 RT: 10.91 AV: 1 NL: 2.09E3  
T: FTMS - c ESI d Full ms2 267.08 @cid35.00 [60.00-]

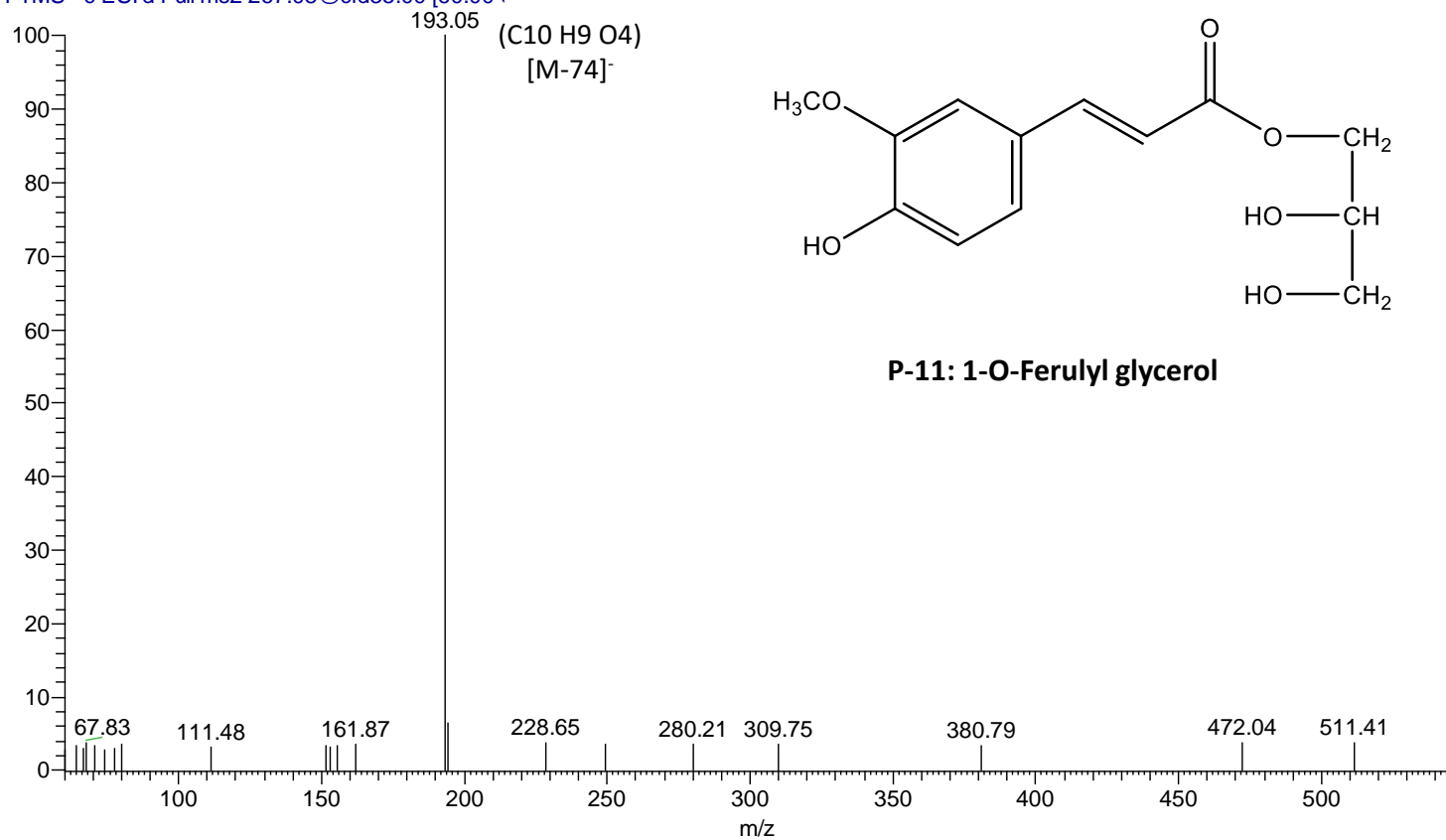

S5 Fig

Supplement: S5 Fig — (PDF) [file pone.0302795.s005.pdf]

PPO-n #2822 RT: 10.91 AV: 1 NL: 1.61E2  
T: FTMS - c ESI d Full ms2 165.05 @cid35.00 [50.00-;

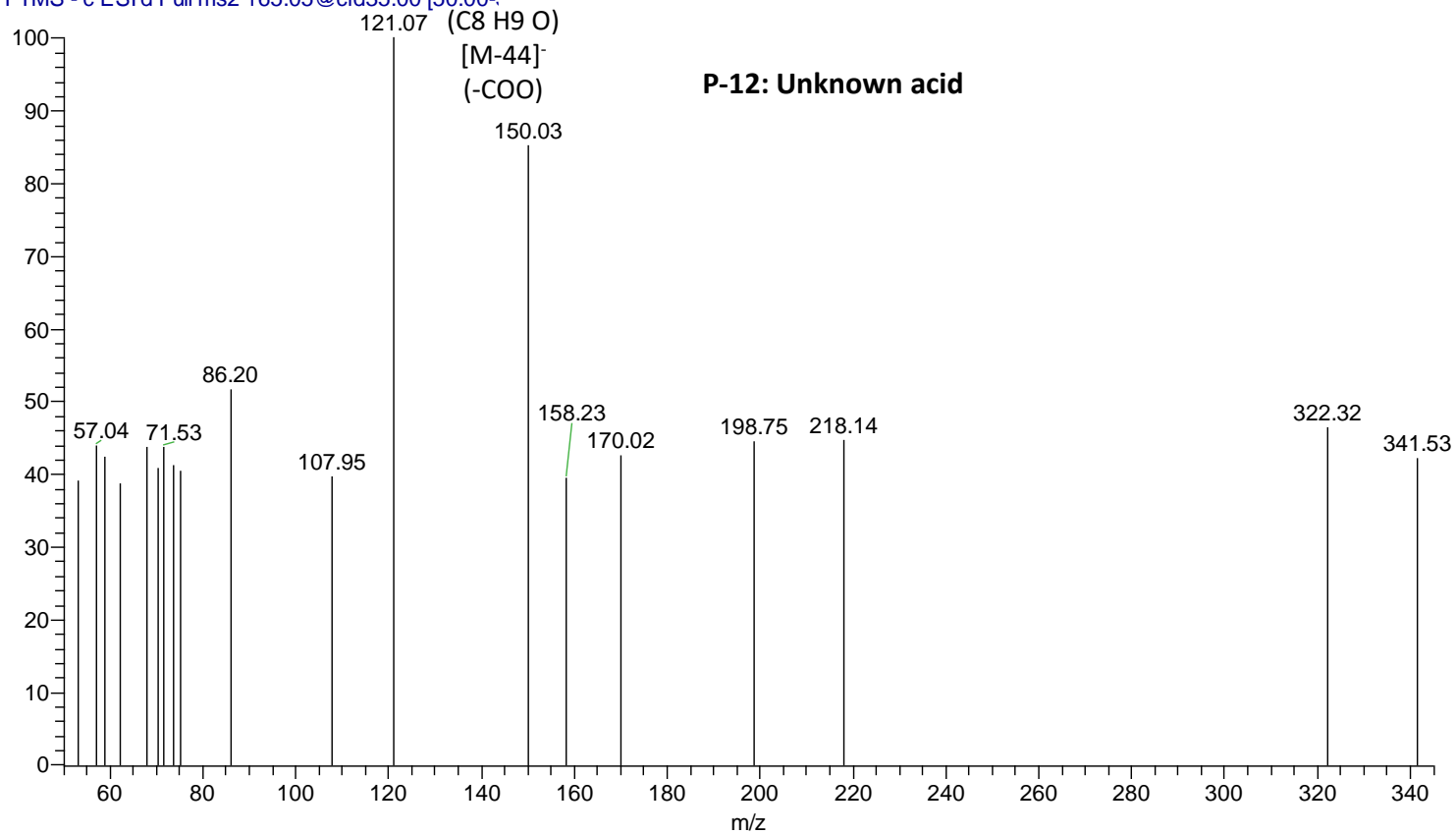

**S6 Fig**

Supplement: S6 Fig — (PDF) [file pone.0302795.s006.pdf]

PPO-n #2918 RT: 11.28 AV: 1 NL: 1.20E3  
T: FTMS - c ESI d Full ms2 445.15@cid35.00 [110.00]

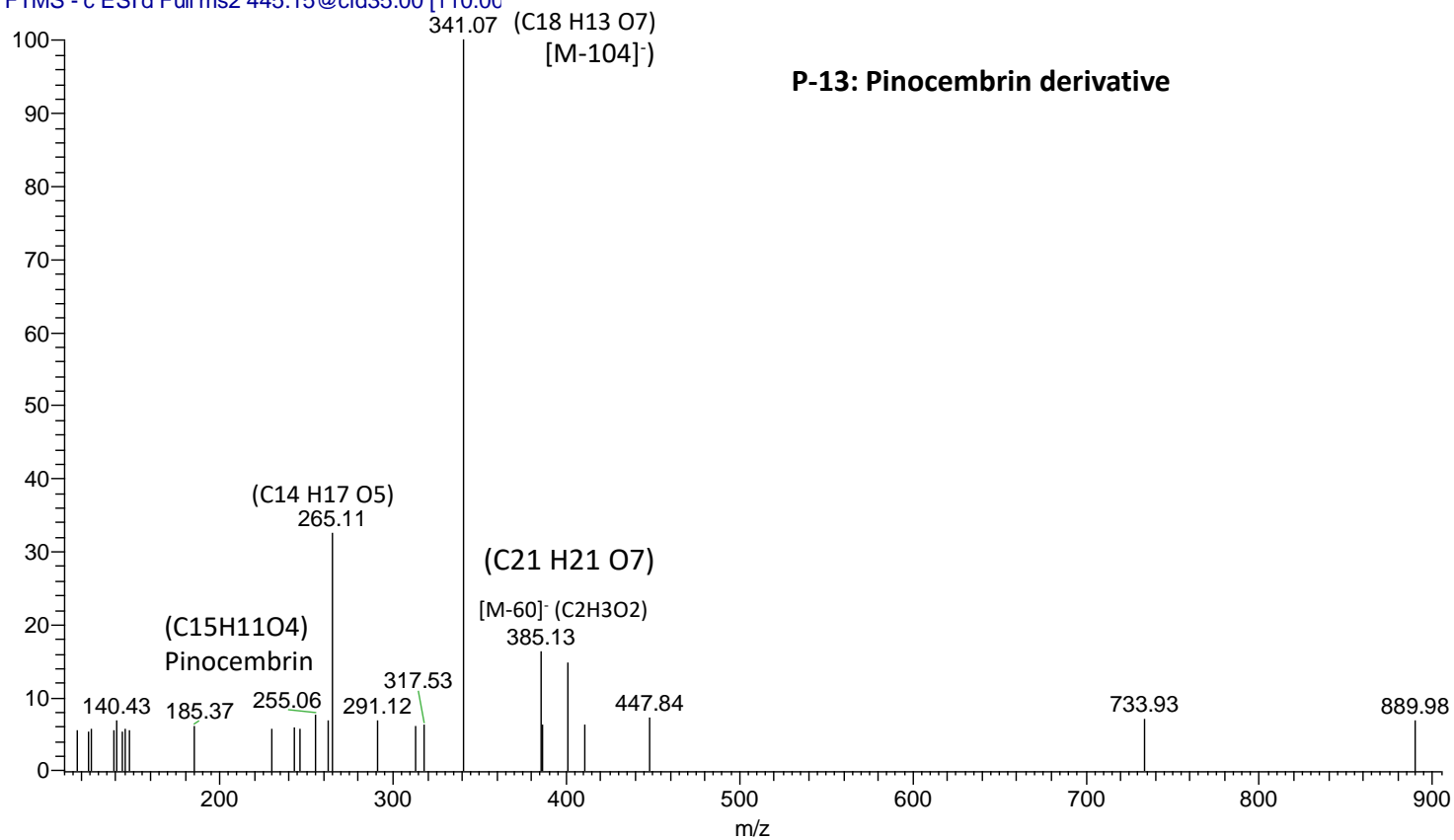

**S7 Fig**

Supplement: S7 Fig — (PDF) [file pone.0302795.s007.pdf]

PPO-n #2940 RT: 11.36 AV: 1 NL: 3.71E3

F: FTMS - c ESI d Full ms2 247.10@cid35.00 [!

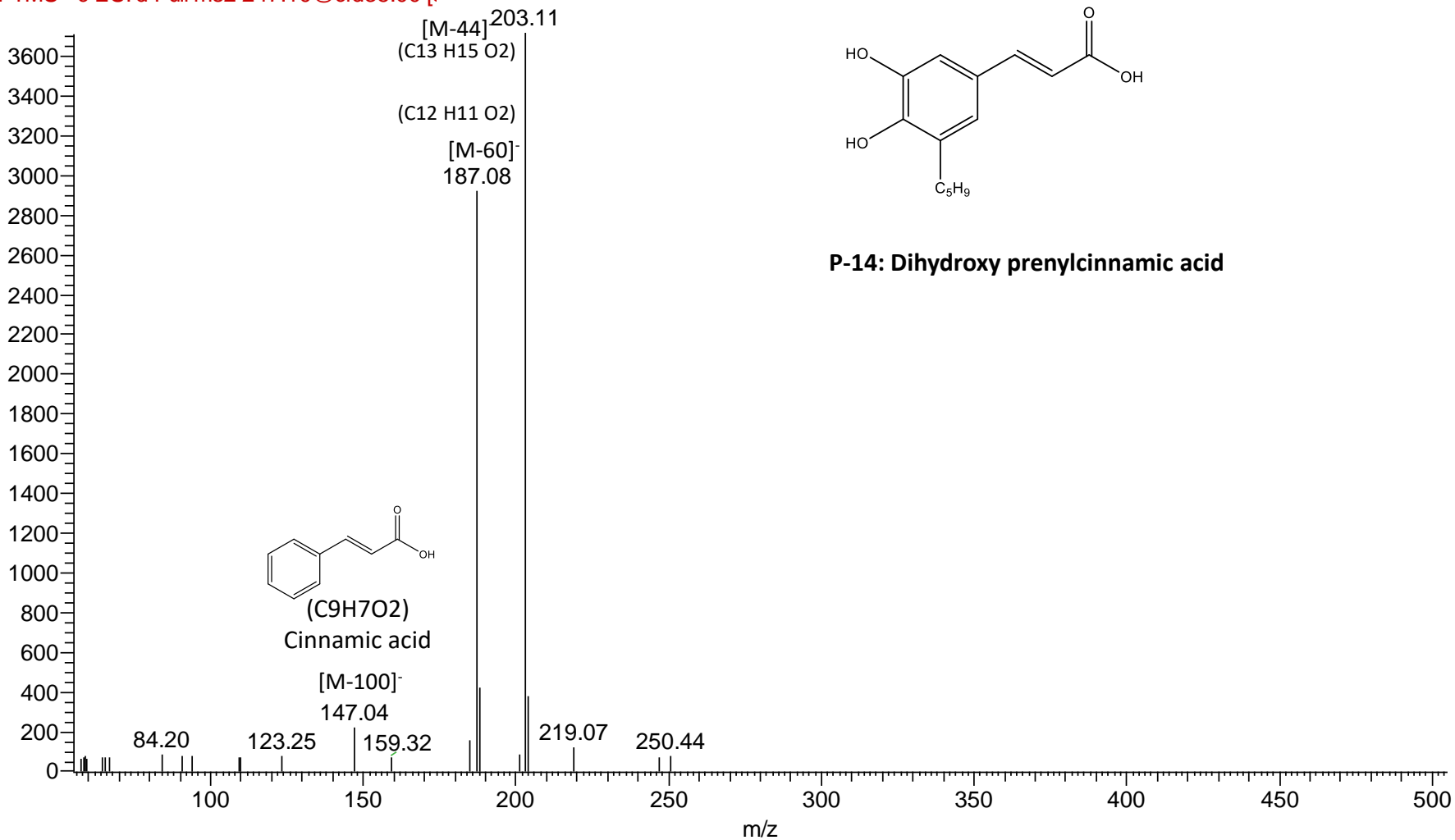

S8 Fig

Supplement: S8 Fig — (PDF) [file pone.0302795.s008.pdf]

PPO-n #3082 RT: 11.90 AV: 1 NL: 3.83E3  
T: FTMS - c ESI d Full ms2 283.06@cid35.00 [65.00-]

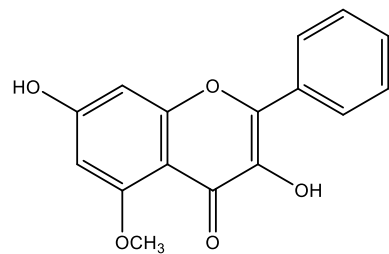

**P-16: Galangin-5-methyl ether**

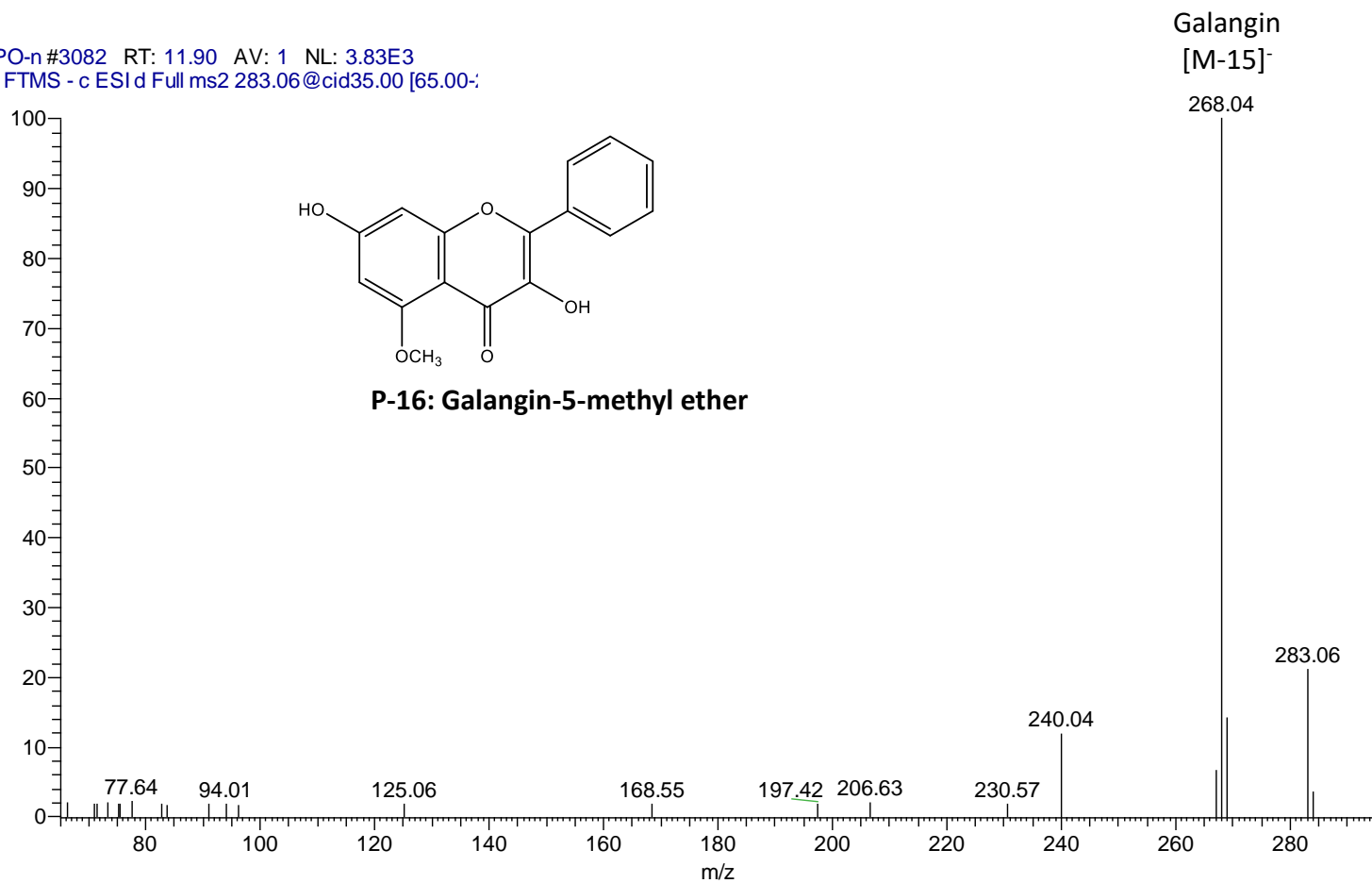

**S9 Fig**

Supplement: S9 Fig — (PDF) [file pone.0302795.s009.pdf]

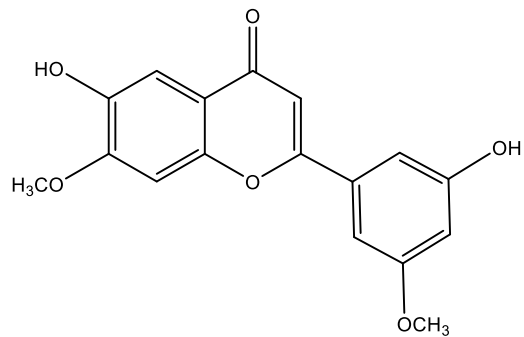

PPO-n #3139 RT: 12.14 AV: 1 NL: 3.43E4  
T: FTMS - p ESI Full ms [100.00-1500.00]

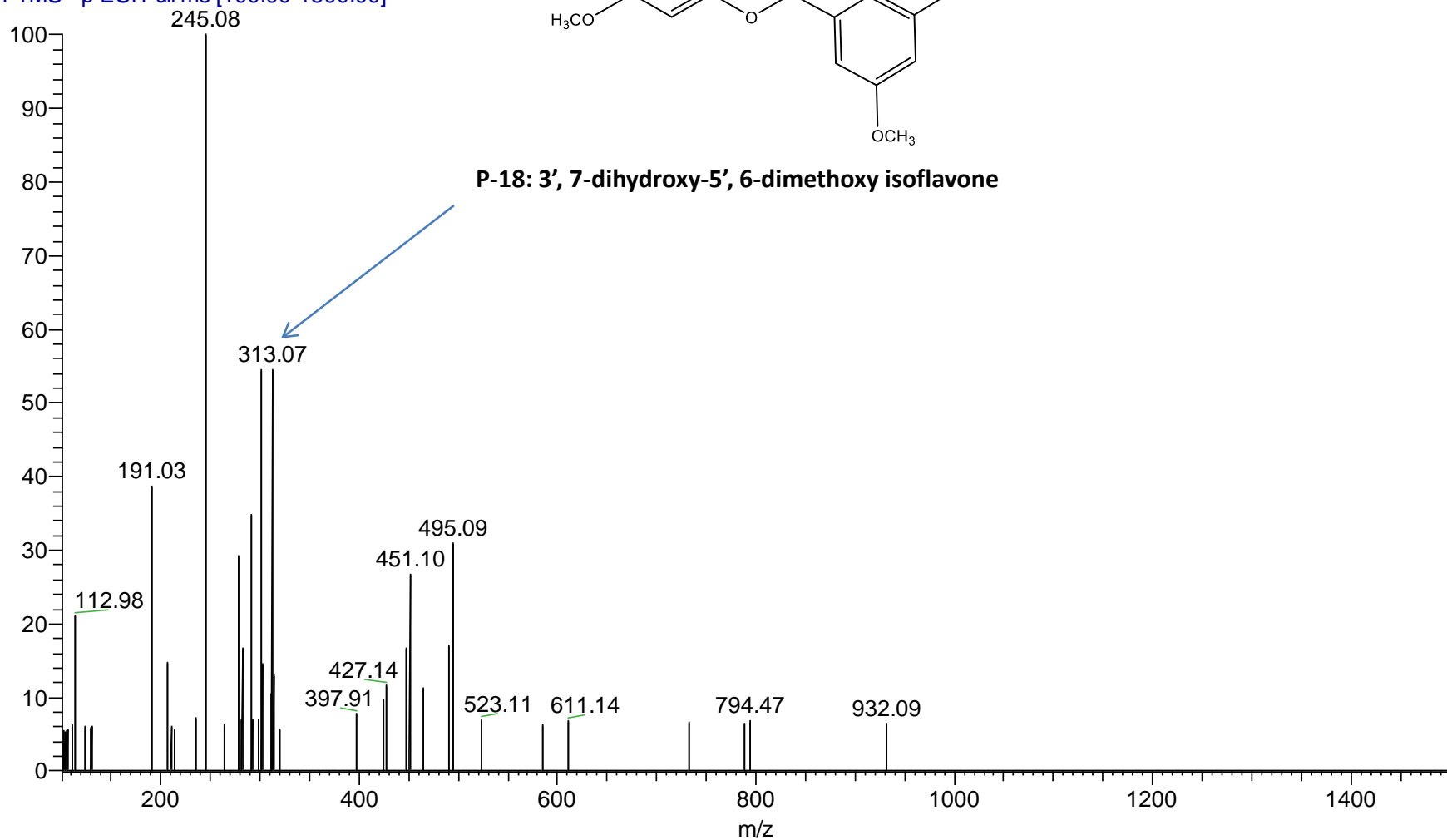

**S10 Fig**

Supplement: S10 Fig — (PDF) [file pone.0302795.s010.pdf]

PPO-n #3162 RT: 12.22 AV: 1 NL: 7.81E3

F: FTMS - c ESI d Full ms2 301.03@cid35.00 [

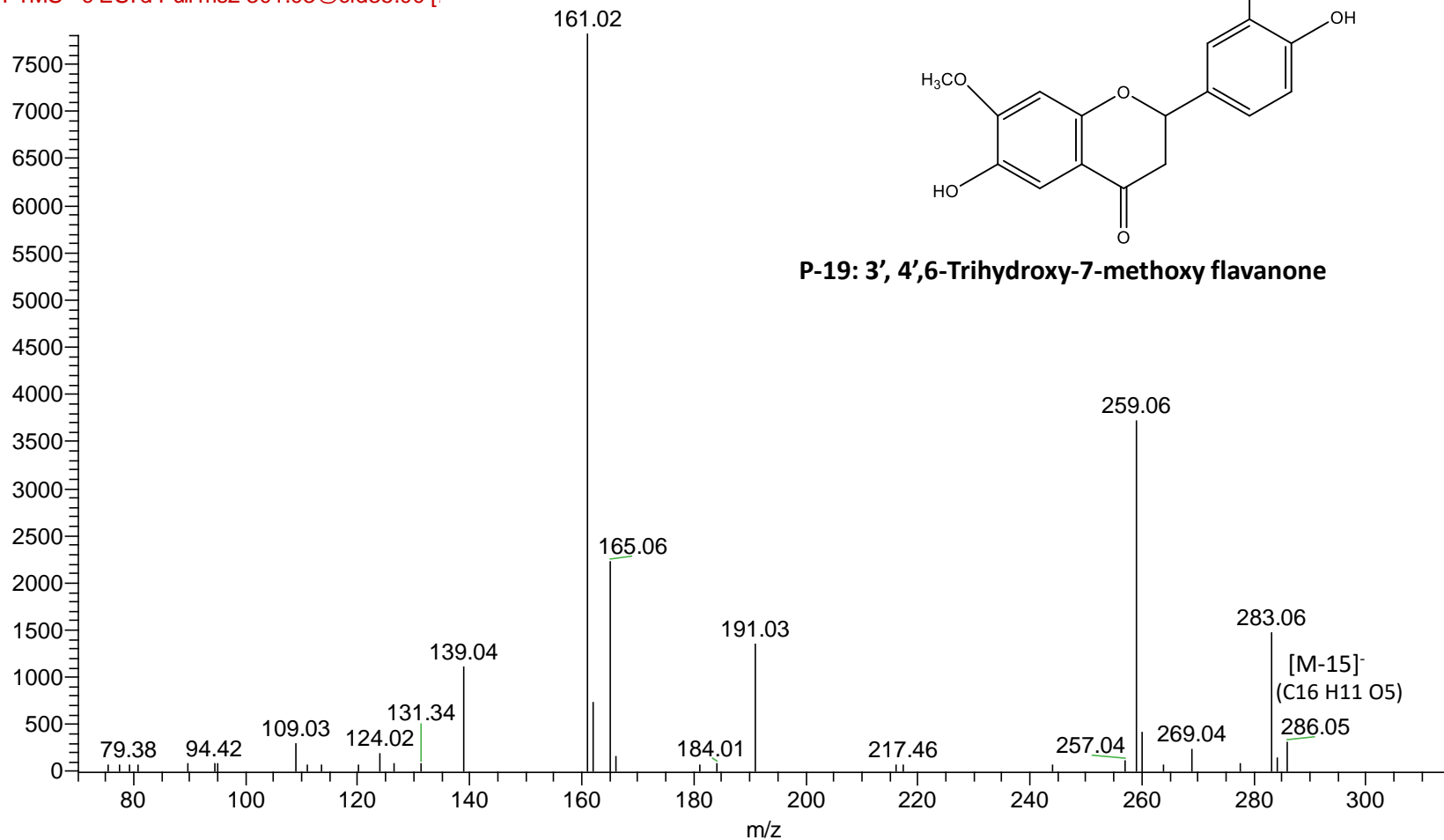

S11 Fig

Supplement: S11 Fig — (PDF) [file pone.0302795.s011.pdf]

PPO-n #3306 RT: 12.74 AV: 1 NL: 7.04E3  
F: FTMS - c ESI d Full ms2 301.03@cid35.00 [

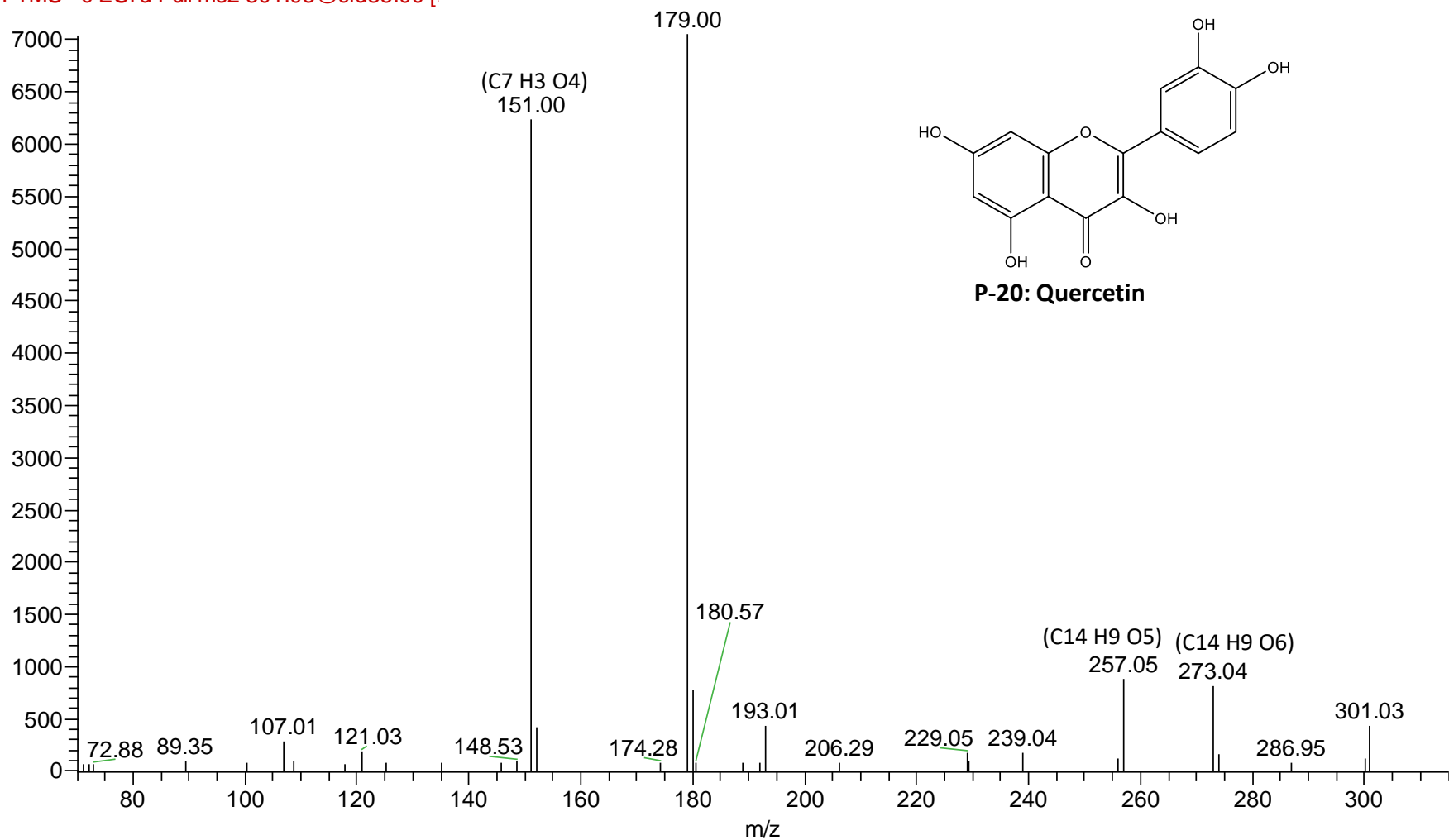

S12 Fig

Supplement: S12 Fig — (PDF) [file pone.0302795.s012.pdf]

PPO-n #3312 RT: 12.77 AV: 1 NL: 1.64E4

F: FTMS - c ESI d Full ms2 463.10@cid35.00 [115.00]

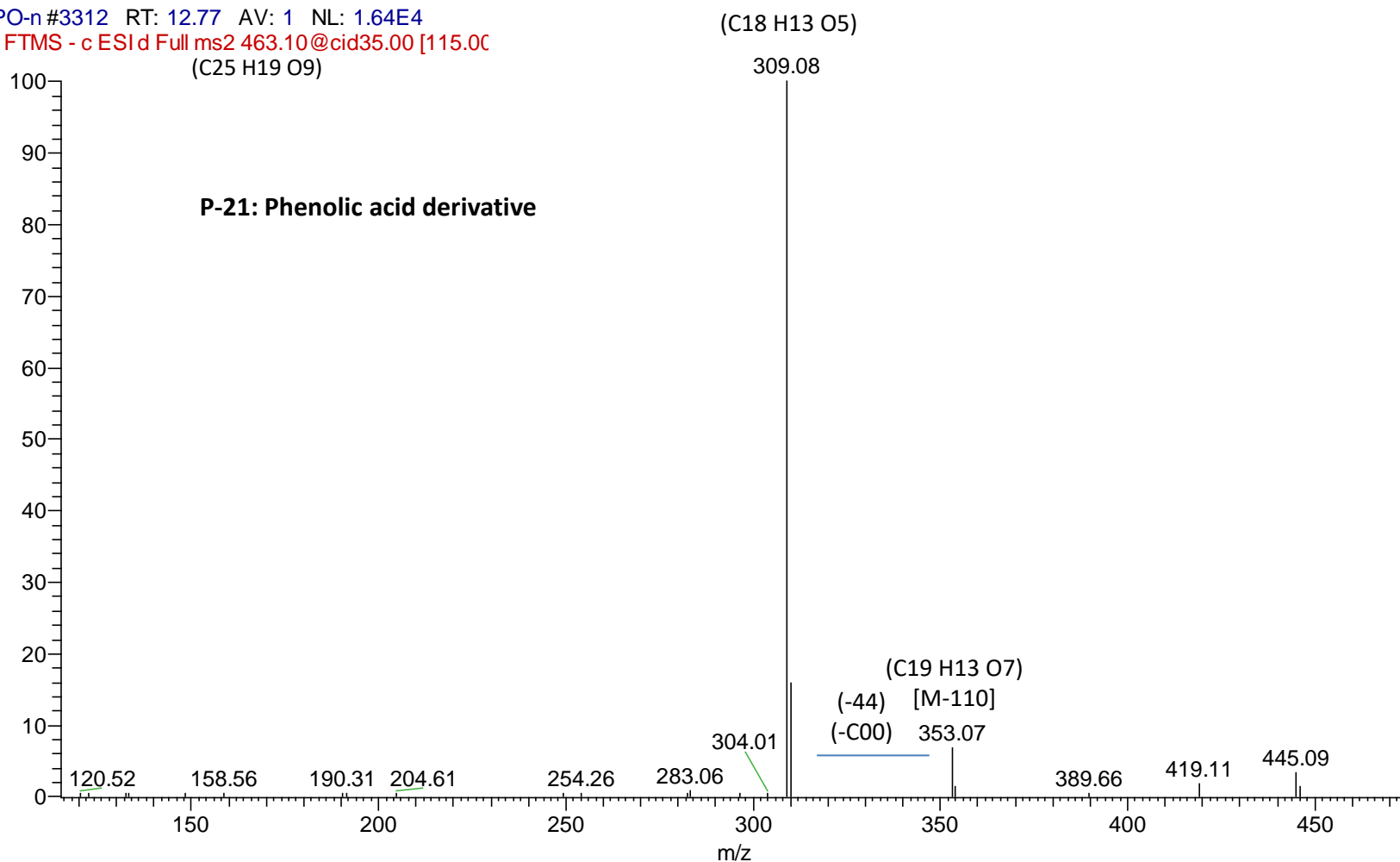

S13 Fig

Supplement: S13 Fig — (PDF) [file pone.0302795.s013.pdf]

PPO-n #3334 RT: 12.85 AV: 1 NL: 4.68E4

F: FTMS - c ESI d Full ms2 285.07@cid35.00 [65.00-:

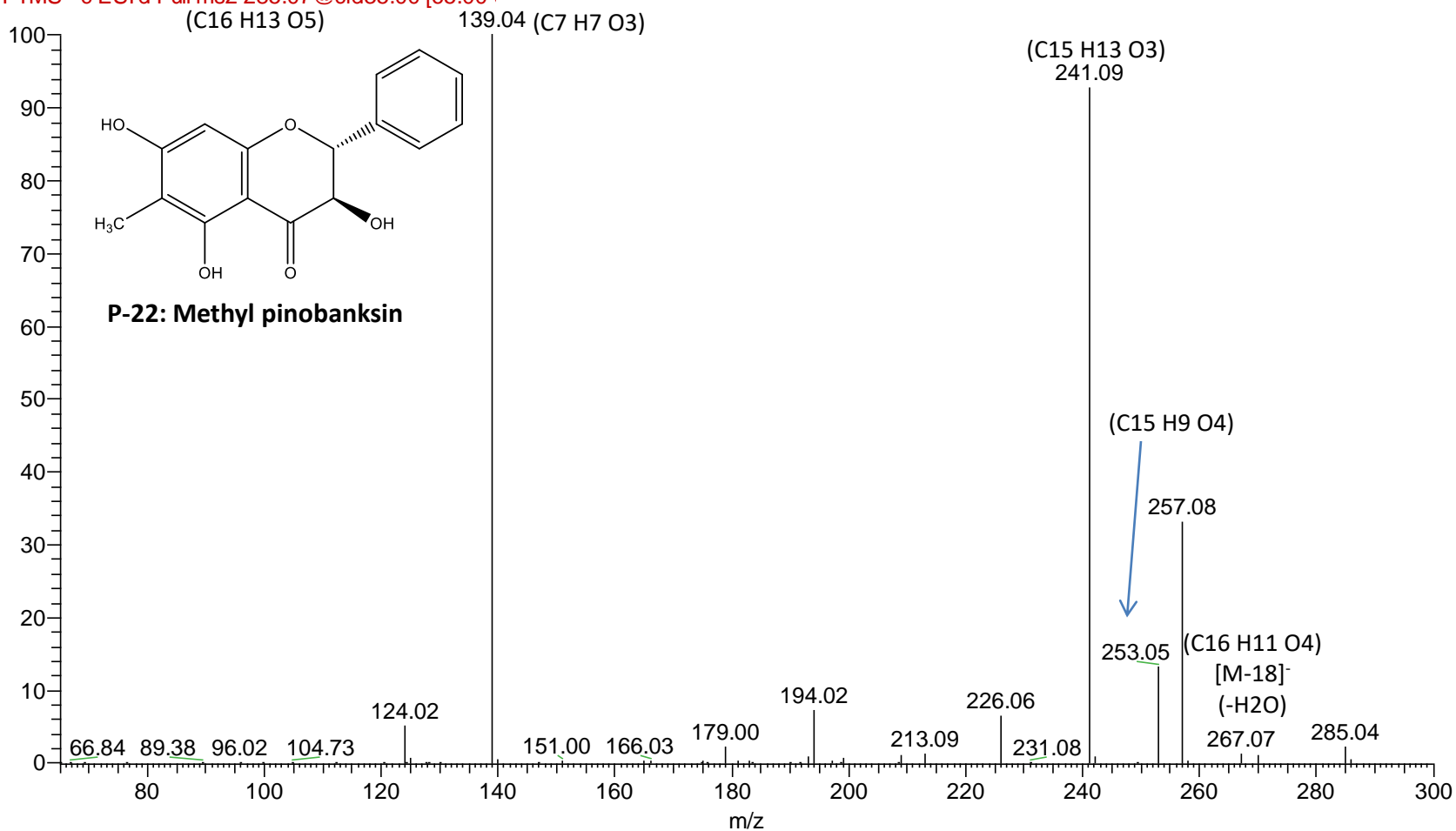

S14 Fig

Supplement: S14 Fig — (PDF) [file pone.0302795.s014.pdf]

PPO-n #3386 RT: 13.04 AV: 1 NL: 1.56E5  
T: FTMS - c ESI d Full ms2 315.05@cid35.00 [75.00-;

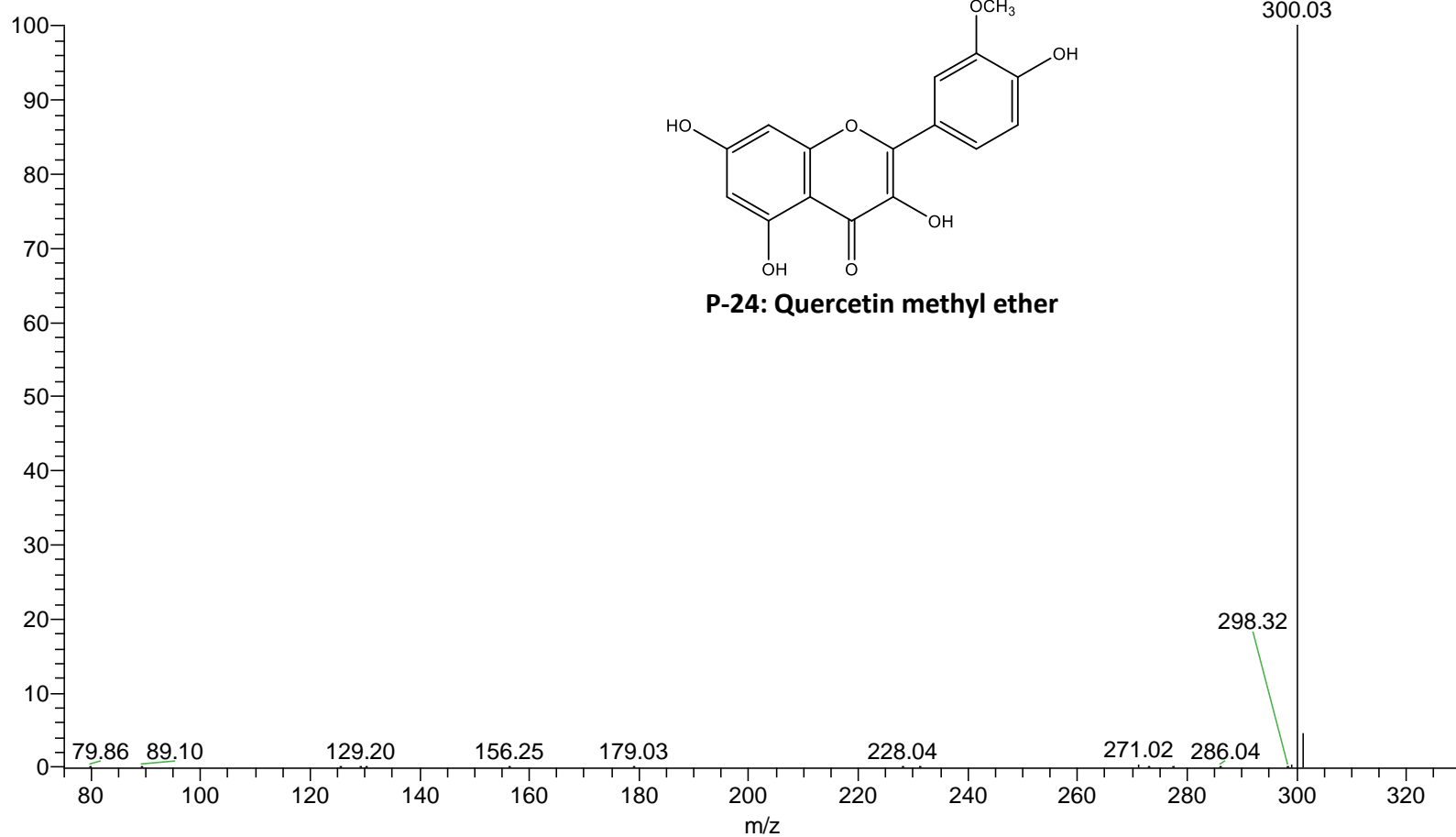

S15 Fig

Supplement: S15 Fig — (PDF) [file pone.0302795.s015.pdf]

PPO-n #3460 RT: 13.31 AV: 1 NL: 4.92E3  
F: FTMS - c ESI d Full ms2 427.14@cid35.00 [105.00  
(C23H23O8)

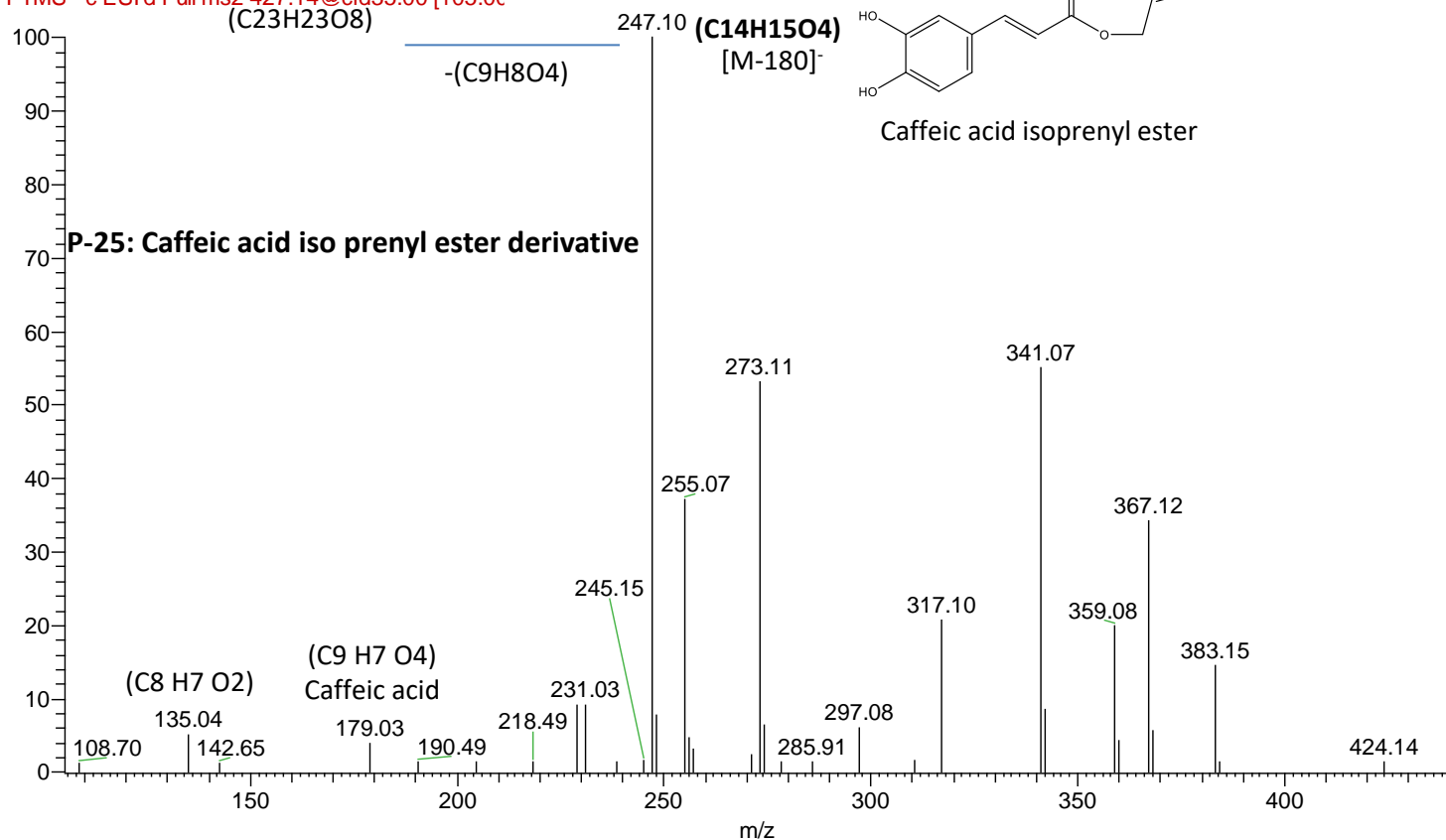

**S16 Fig**

Supplement: S16 Fig — (PDF) [file pone.0302795.s016.pdf]

PPO-n #3620 RT: 13.89 AV: 1 NL: 2.45E5  
T: FTMS - c ESI d Full ms2 299.06@cid35.00 [70.00-:

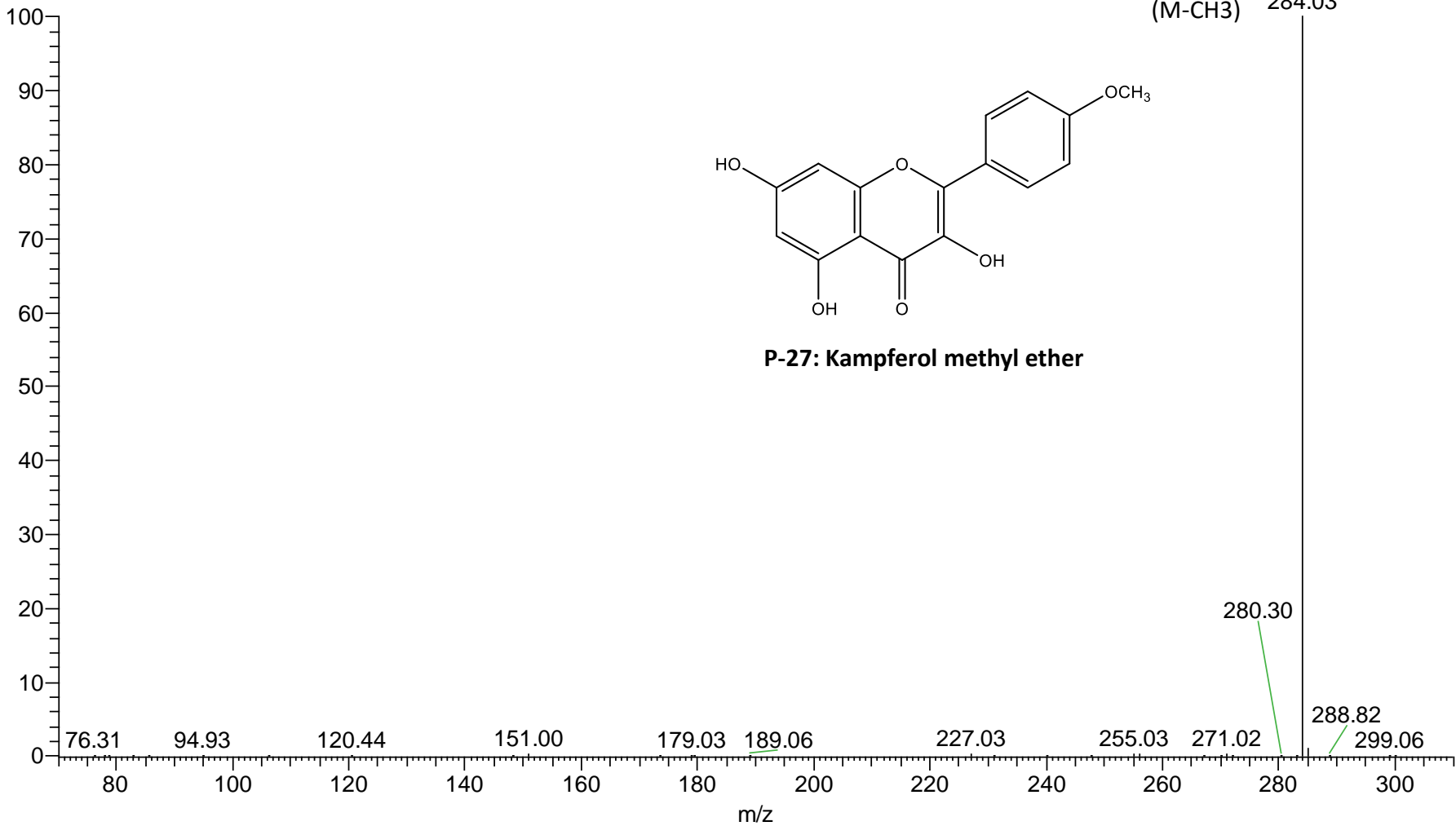

S17 Fig

Supplement: S17 Fig — (PDF) [file pone.0302795.s017.pdf]

PPO-n #3664 RT: 14.05 AV: 1 NL: 3.22E5  
T: FTMS - c ESI d Full ms2 329.07@cid35.00 [80.00-:

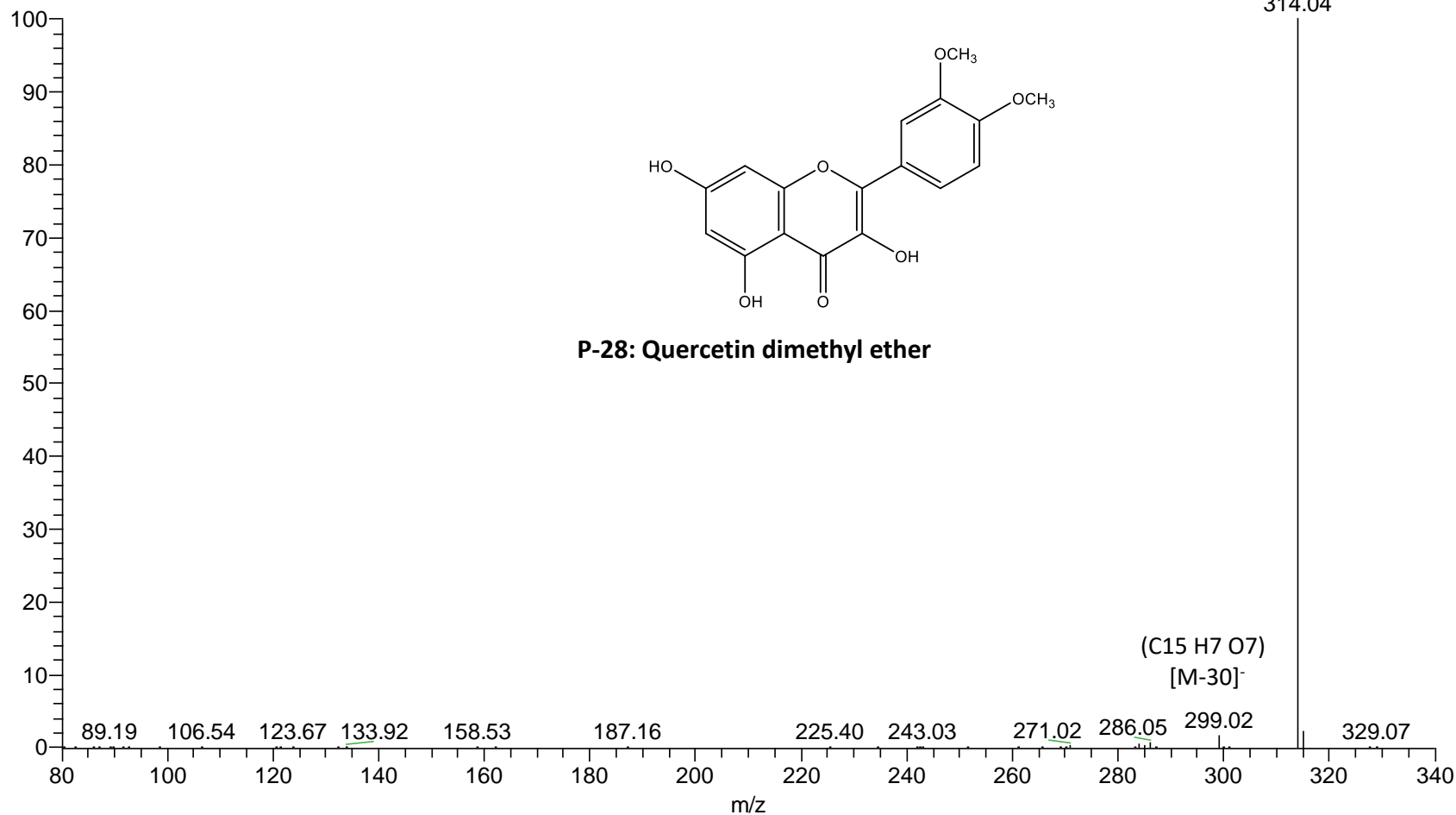

**S18 Fig**

Supplement: S18 Fig — (PDF) [file pone.0302795.s018.pdf]

PPO-n #3714 RT: 14.23 AV: 1 NL: 3.72E5  
T: FTMS - c ESI d Full ms2 435.11 @cid35.00 [105.00]

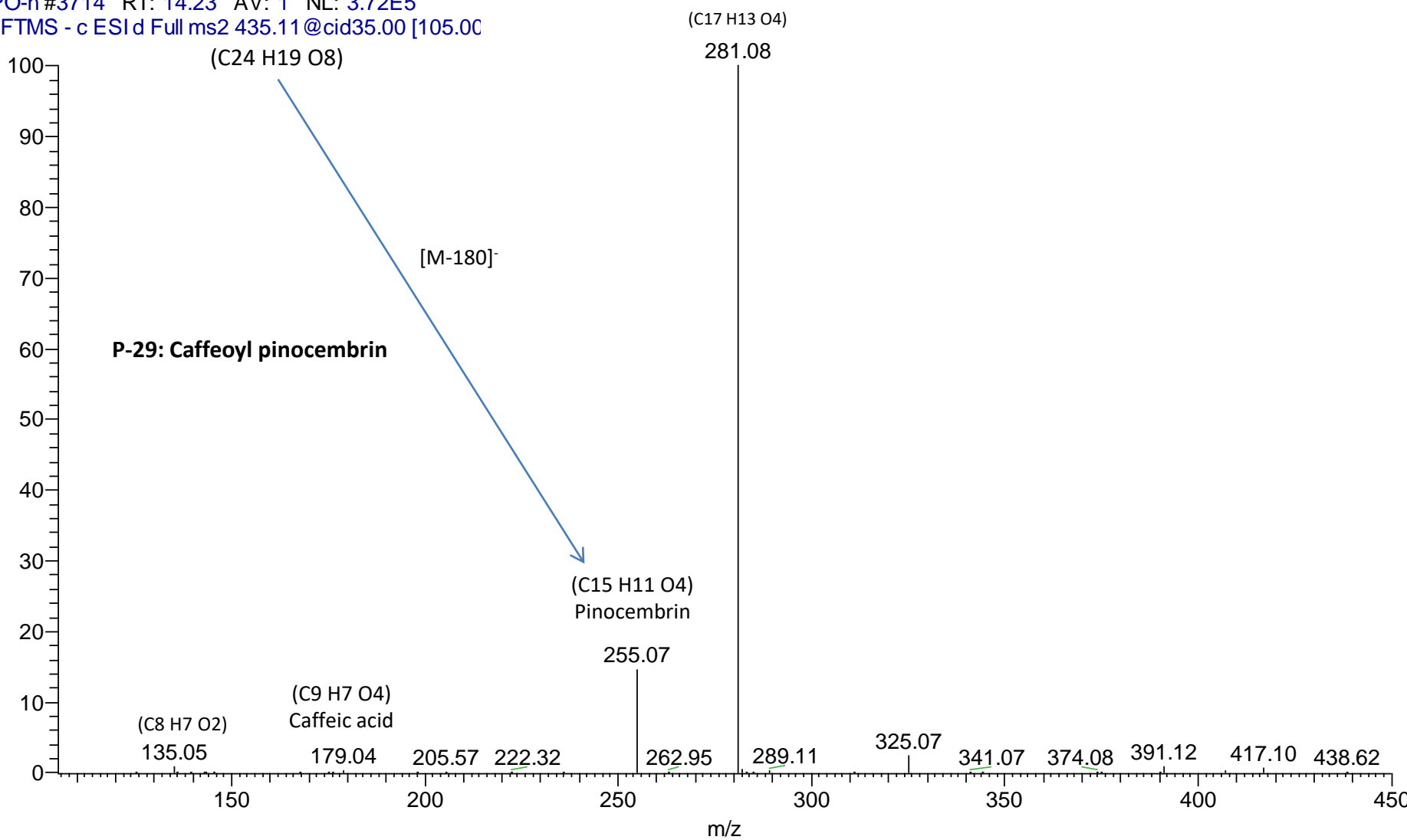

S19 Fig

Supplement: S19 Fig — (PDF) [file pone.0302795.s019.pdf]

PPO-n #3764 RT: 14.41 AV: 1 NL: 1.93E5  
T: FTMS - c ESI d Full ms2 283.06@cid35.00 [65.00-:

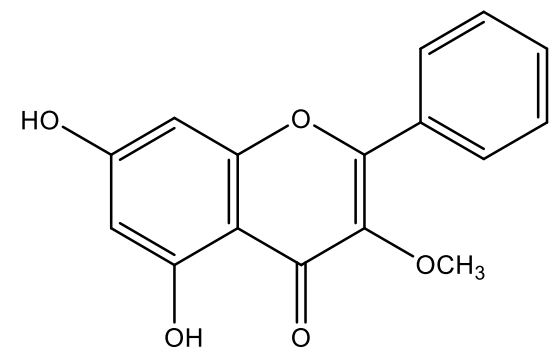

P-30: Galangin -3-methyl ether

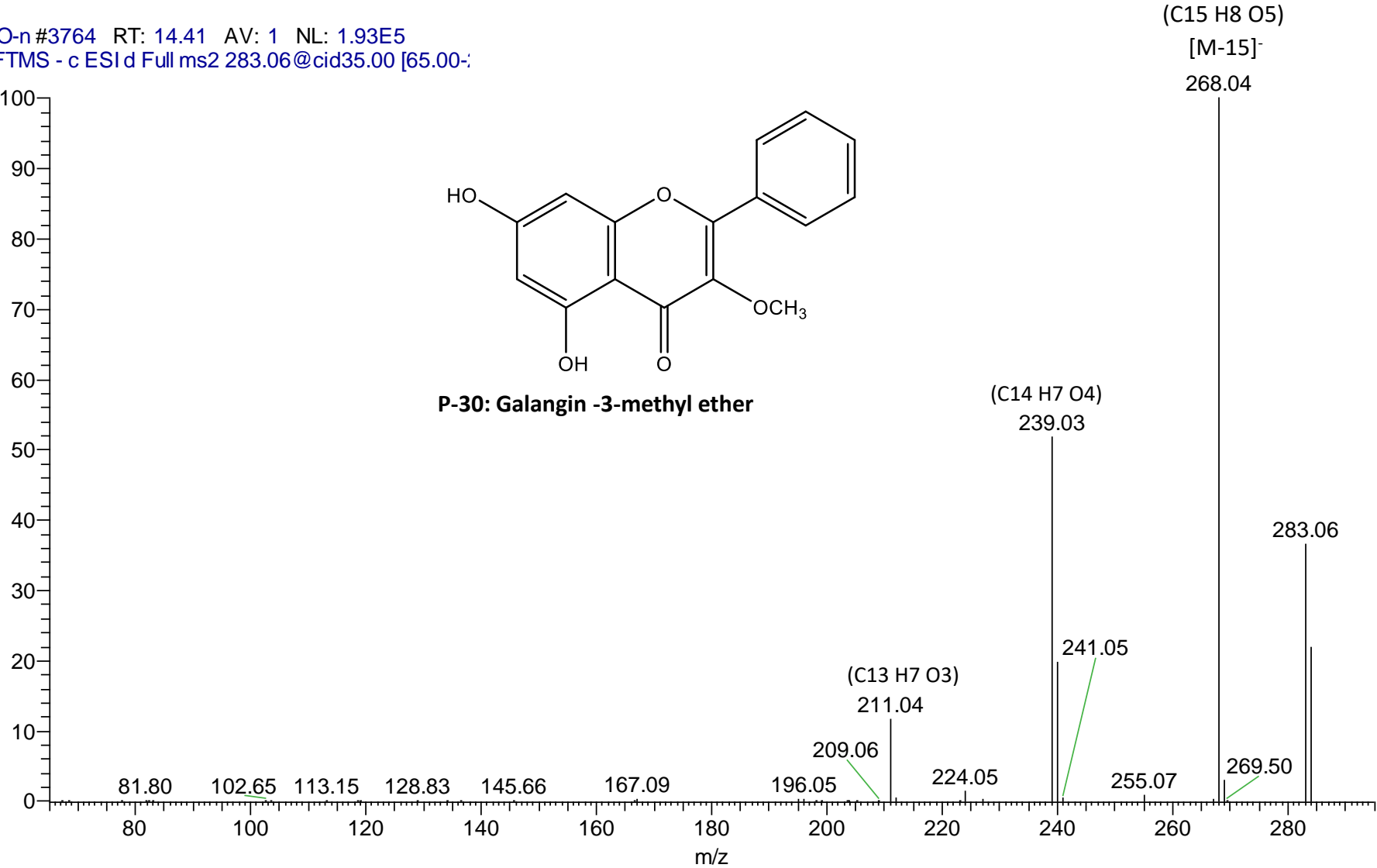

S20 Fig

Supplement: S20 Fig — (PDF) [file pone.0302795.s020.pdf]

PPO-n #3832 RT: 14.66 AV: 1 NL: 6.85E4  
T: FTMS - c ESI d Full ms2 447.11 @cid35.00 [-

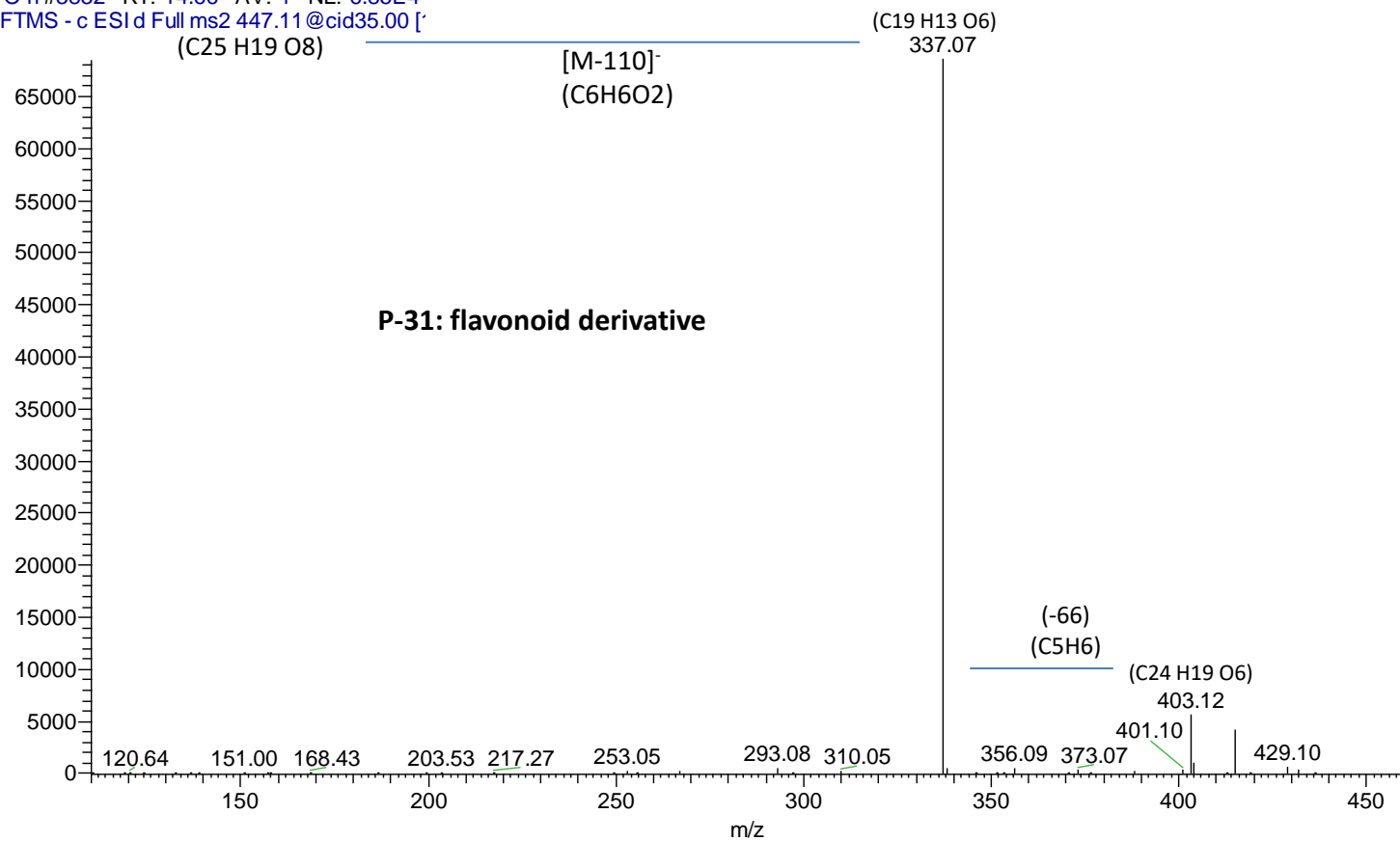

**S21 Fig**

Supplement: S21 Fig — (PDF) [file pone.0302795.s021.pdf]

PPO-n #3828 RT: 14.64 AV: 1 NL: 5.22E4  
T: FTMS - c ESI d Full ms2 407.11 @cid35.00 [100.00

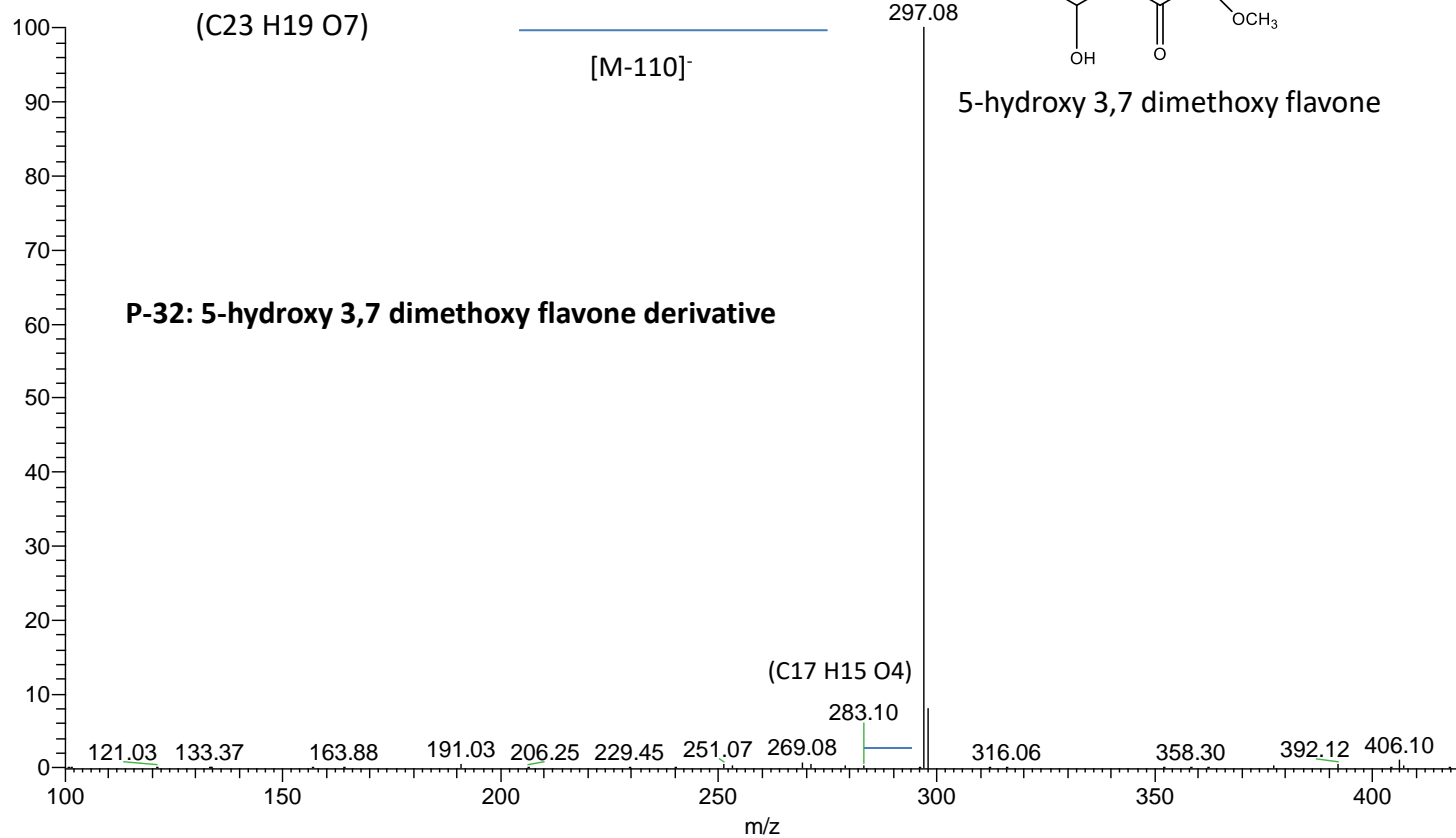

S22 Fig

Supplement: S22 Fig — (PDF) [file pone.0302795.s022.pdf]

PPO-n #3854 RT: 14.74 AV: 1 NL: 6.66E4

F: FTMS - c ESI d Full ms2 419.11 @cid35.00 [

(C23 H19 O5)

[M-44]<sup>-</sup>

(-COO)

375.12

(C24 H19 O7)

**P-33: P-coumaroyl trihydroxyflavone**

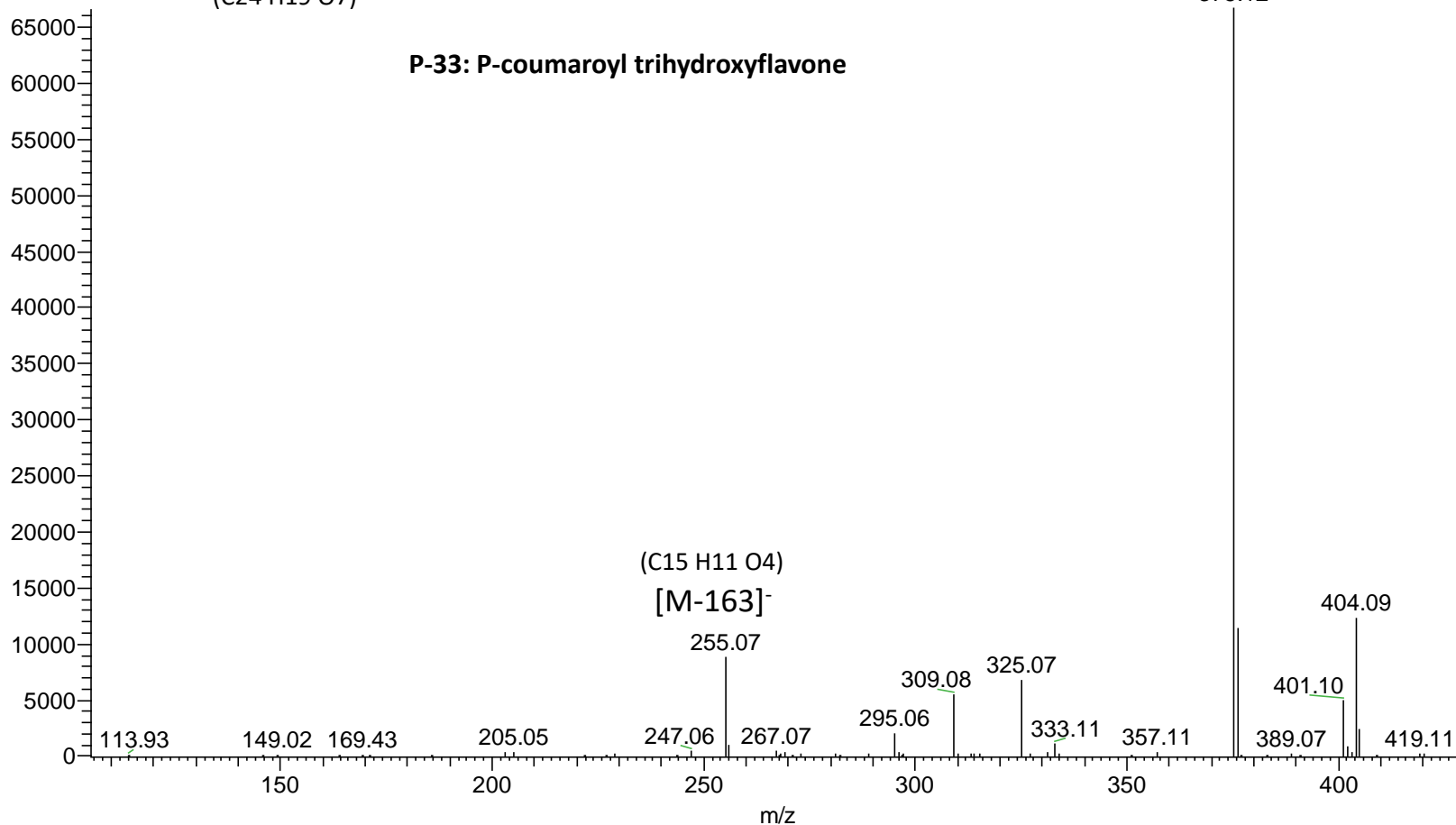

**S23 Fig**

Supplement: S23 Fig — (PDF) [file pone.0302795.s023.pdf]

PPO-n #4142 RT: 15.68 AV: 1 NL: 1.92E5  
T: FTMS - c ESI d Full ms2 477.12@cid35.00 [120.00]

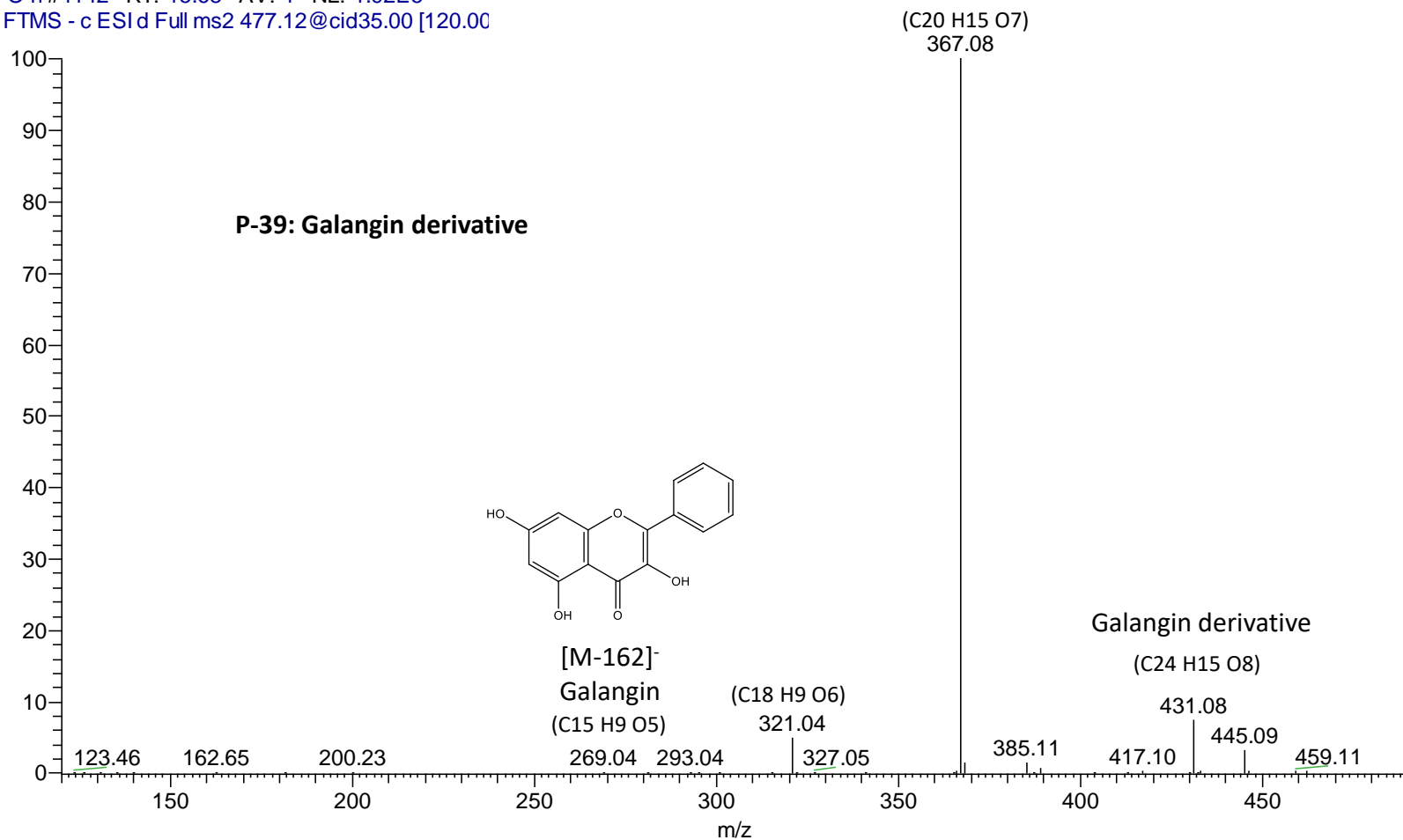

S24 Fig

Supplement: S24 Fig — (PDF) [file pone.0302795.s024.pdf]

PPO-n #4168 RT: 15.77 AV: 1 NL: 6.09E5  
T: FTMS - c ESI d Full ms2 463.14@cid35.00 [115.00]

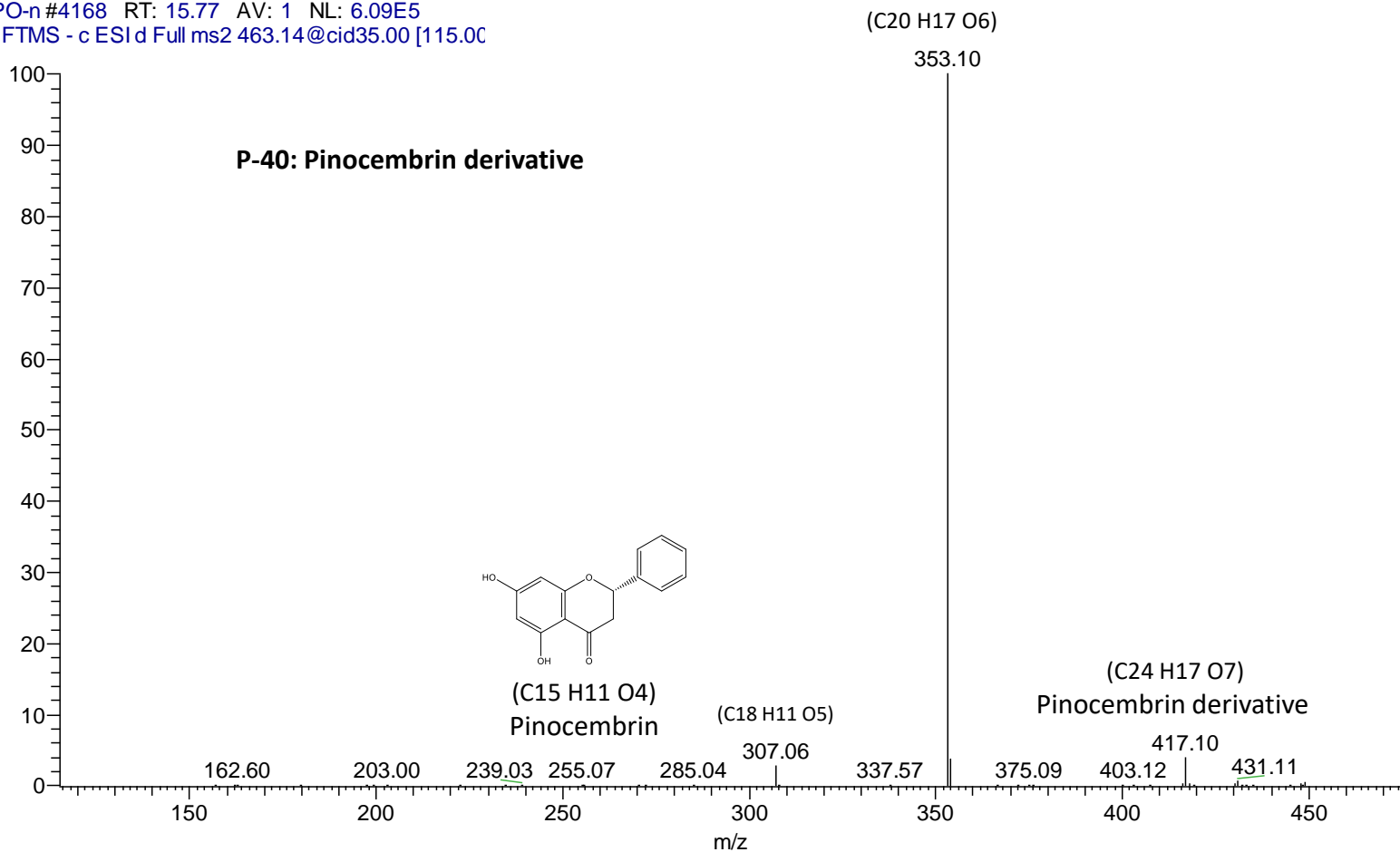

**S25 Fig**

Supplement: S25 Fig — (PDF) [file pone.0302795.s025.pdf]

PPO-n #4200 RT: 15.88 AV: 1 NL: 2.10E5  
T: FTMS - c ESI d Full ms2 461.12@cid35.00 [115.00]

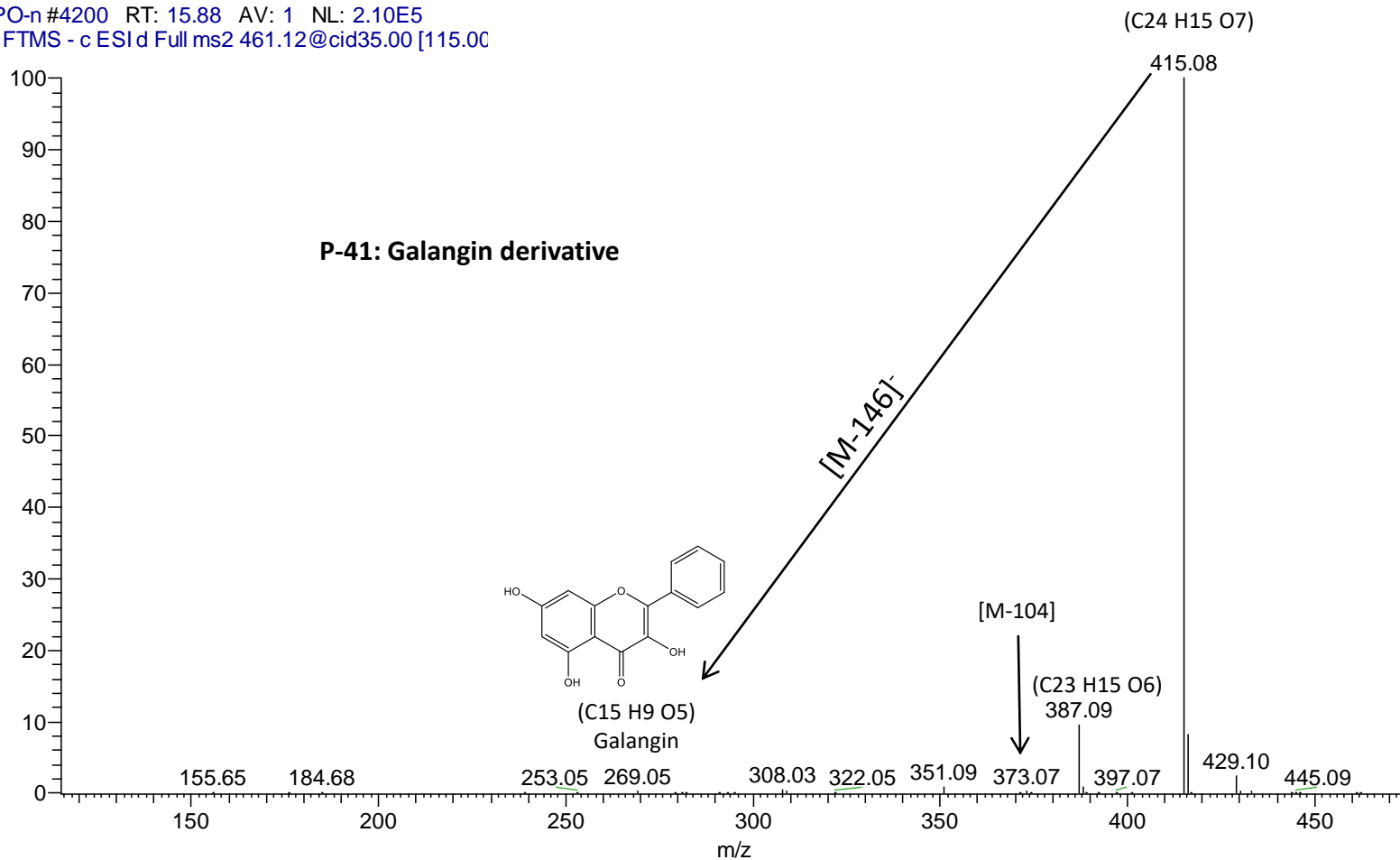

S26 Fig

Supplement: S26 Fig — (PDF) [file pone.0302795.s026.pdf]

PPO-n #4278 RT: 16.16 AV: 1 NL: 2.55E5  
T: FTMS - c ESI d Full ms2 389.10@cid35.00 [95.00--

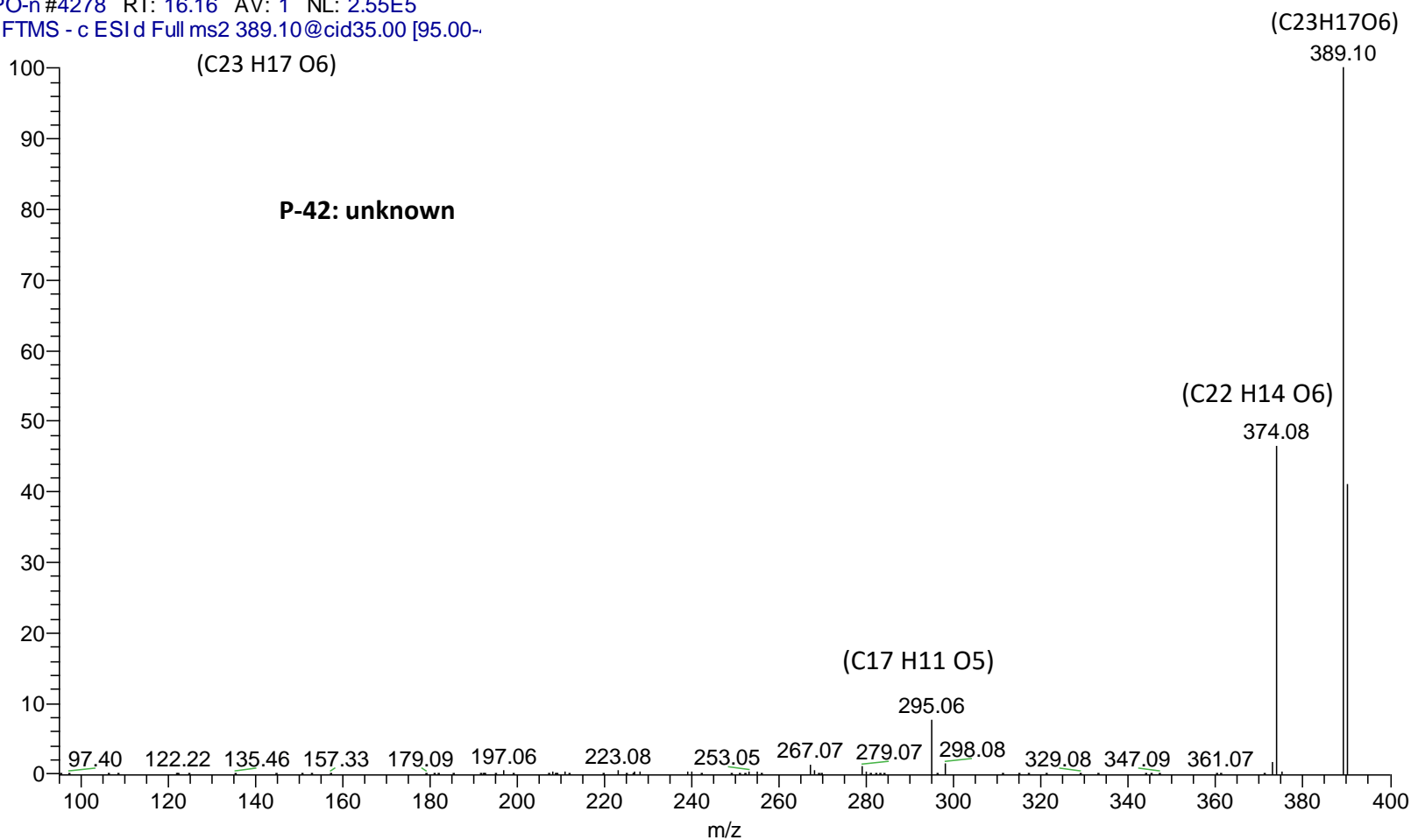

S27 Fig

Supplement: S27 Fig — (PDF) [file pone.0302795.s027.pdf]

PPO-n #4446 RT: 16.79 AV: 1 NL: 8.18E4  
F: FTMS - c ESI d Full ms2 405.10@cid35.00 [

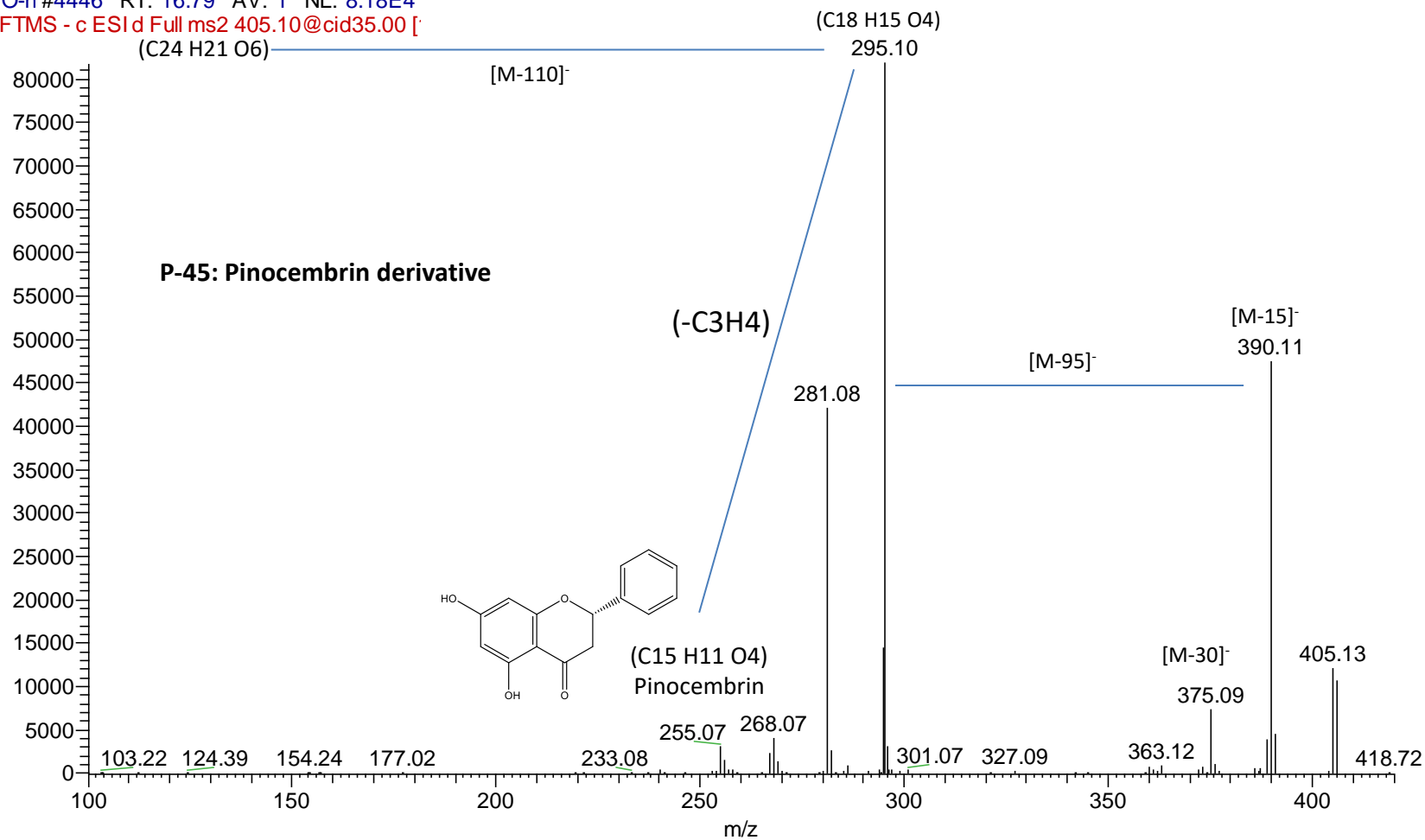

S29 Fig

Supplement: S29 Fig — (PDF) [file pone.0302795.s029.pdf]

PPO-n #4492 RT: 16.94 AV: 1 NL: 8.34E4  
T: FTMS - c ESI d Full ms2 403.12@cid35.00 [100.00]

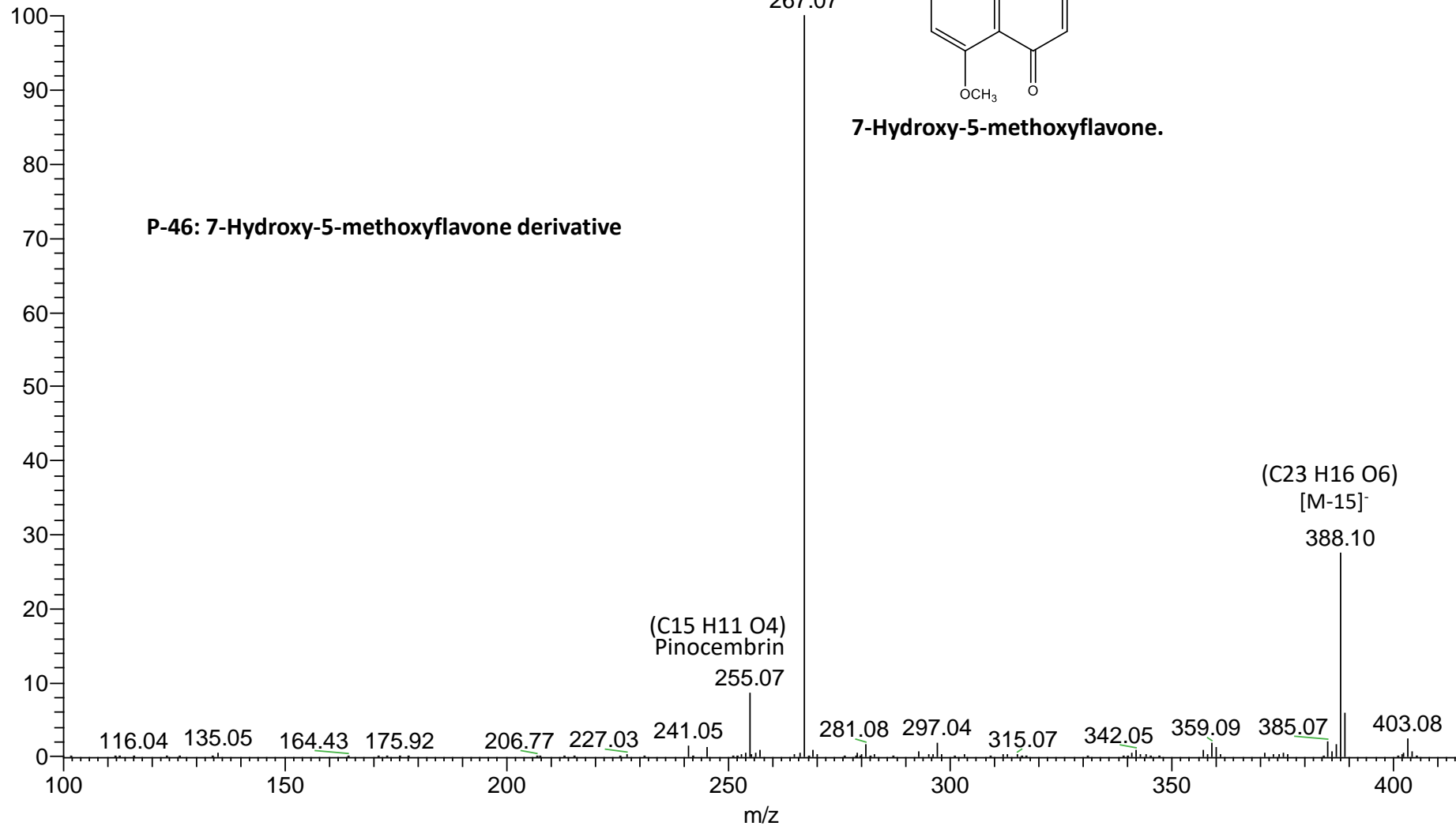

S30 Fig

Supplement: S30 Fig — (PDF) [file pone.0302795.s030.pdf]

PPO-n #4518 RT: 17.04 AV: 1 NL: 6.23E4  
T: FTMS - c ESI d Full ms2 433.13@cid35.00 [105.00]

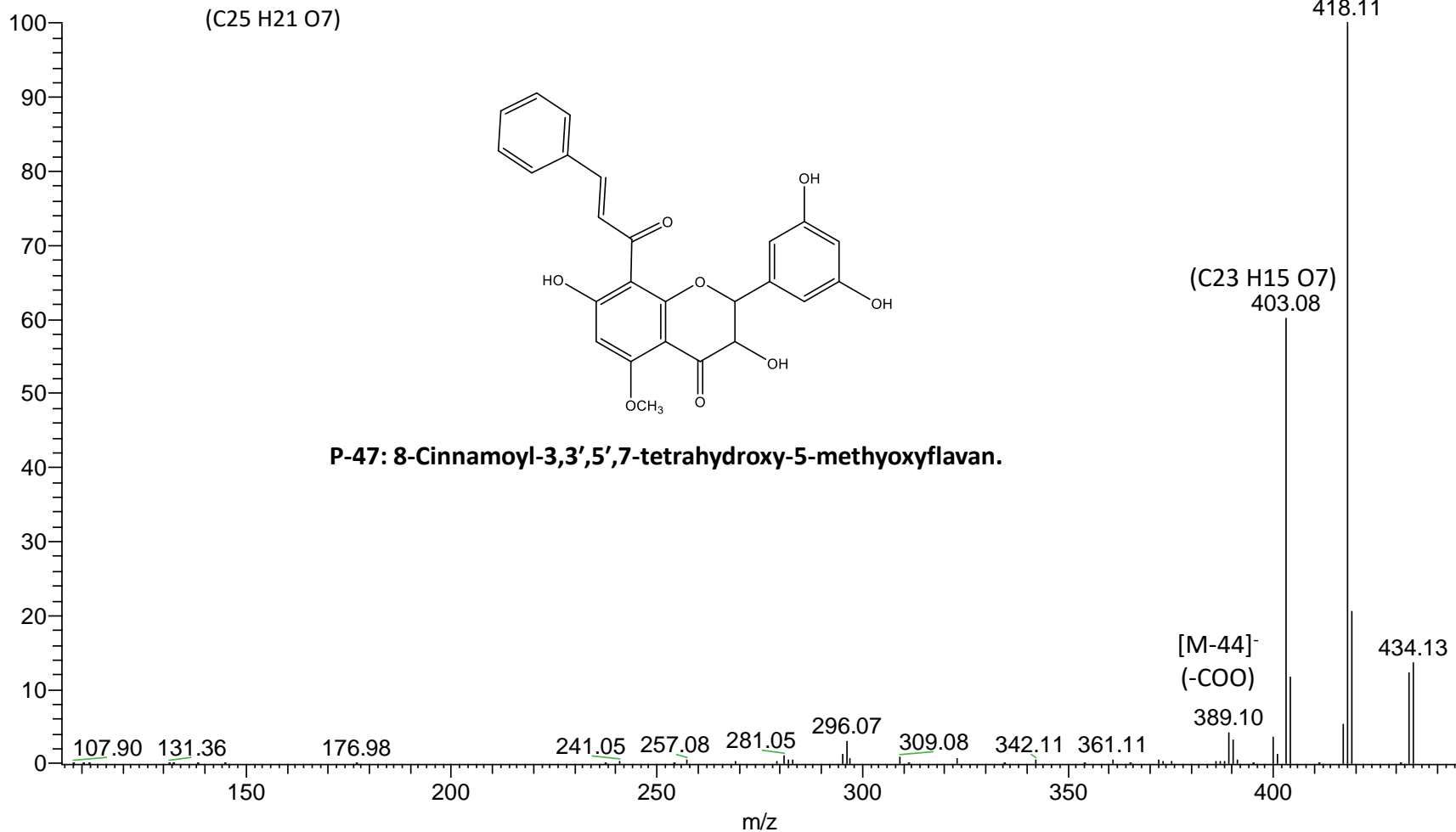

S31 Fig

Supplement: S31 Fig — (PDF) [file pone.0302795.s031.pdf]

PPO-n #4526 RT: 17.07 AV: 1 NL: 1.14E5  
T: FTMS - c ESI d Full ms2 341.10@cid35.00 [80.00-;

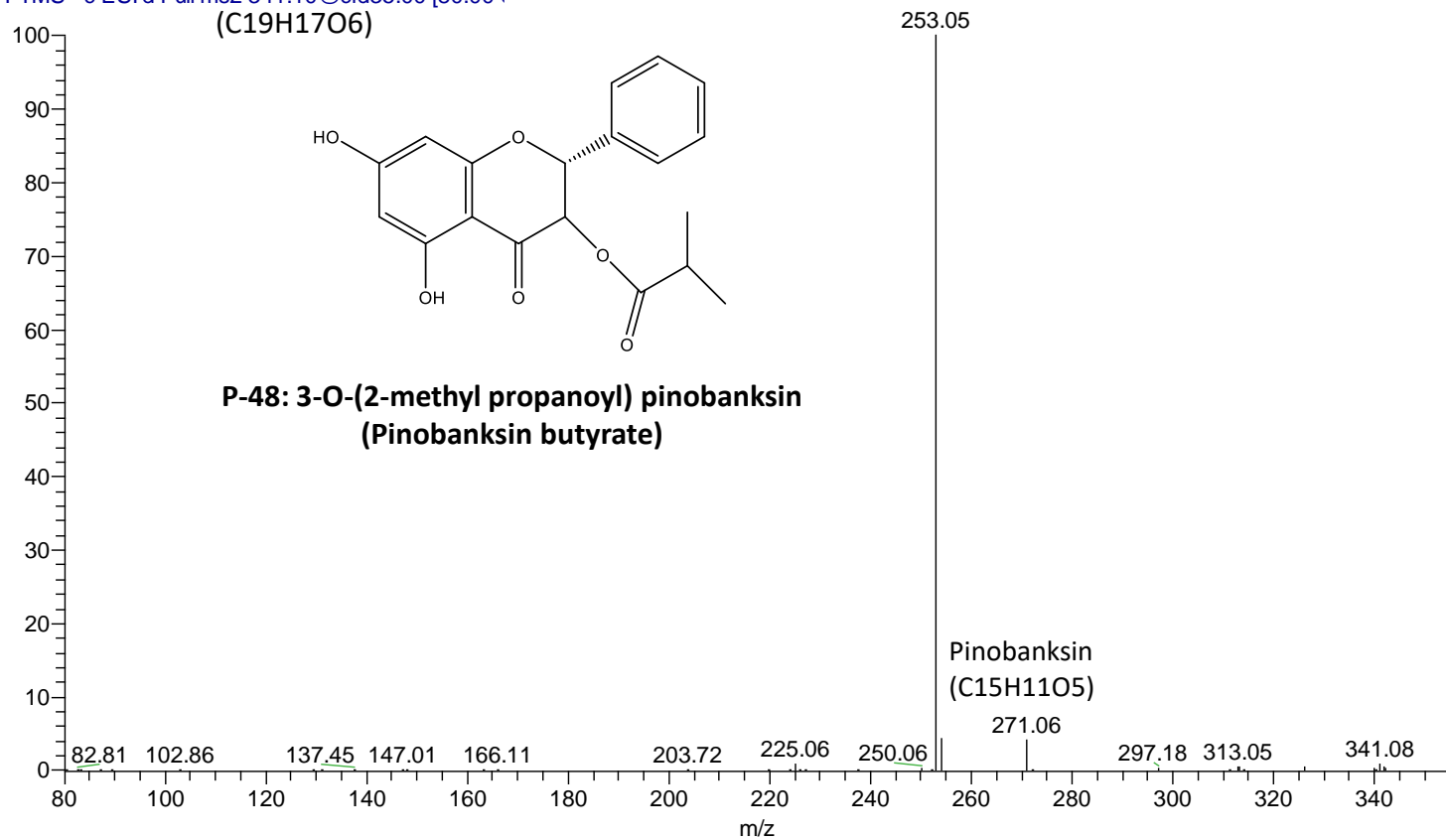

S32 Fig

Supplement: S32 Fig — (PDF) [file pone.0302795.s032.pdf]

PPO-n #4600 RT: 17.33 AV: 1 NL: 2.56E4  
T: FTMS - c ESI d Full ms2 387.09@cid35.00 [95.0]

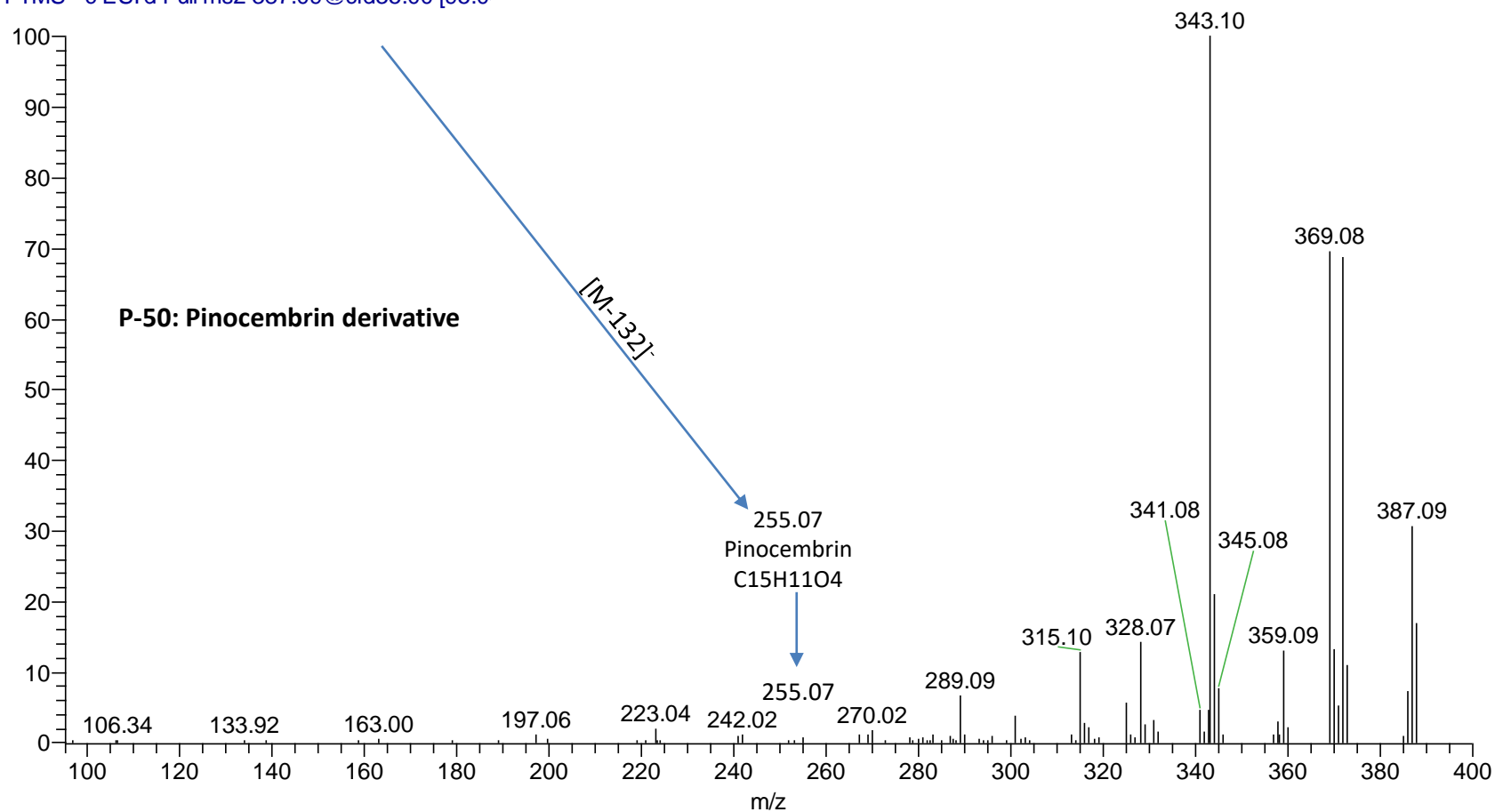

**S33 Fig**

Supplement: S33 Fig — (PDF) [file pone.0302795.s033.pdf]

PPO-n #4664 RT: 17.57 AV: 1 NL: 2.14E4  
T: FTMS - c ESI d Full ms2 455.24@cid35.00 [115.00]

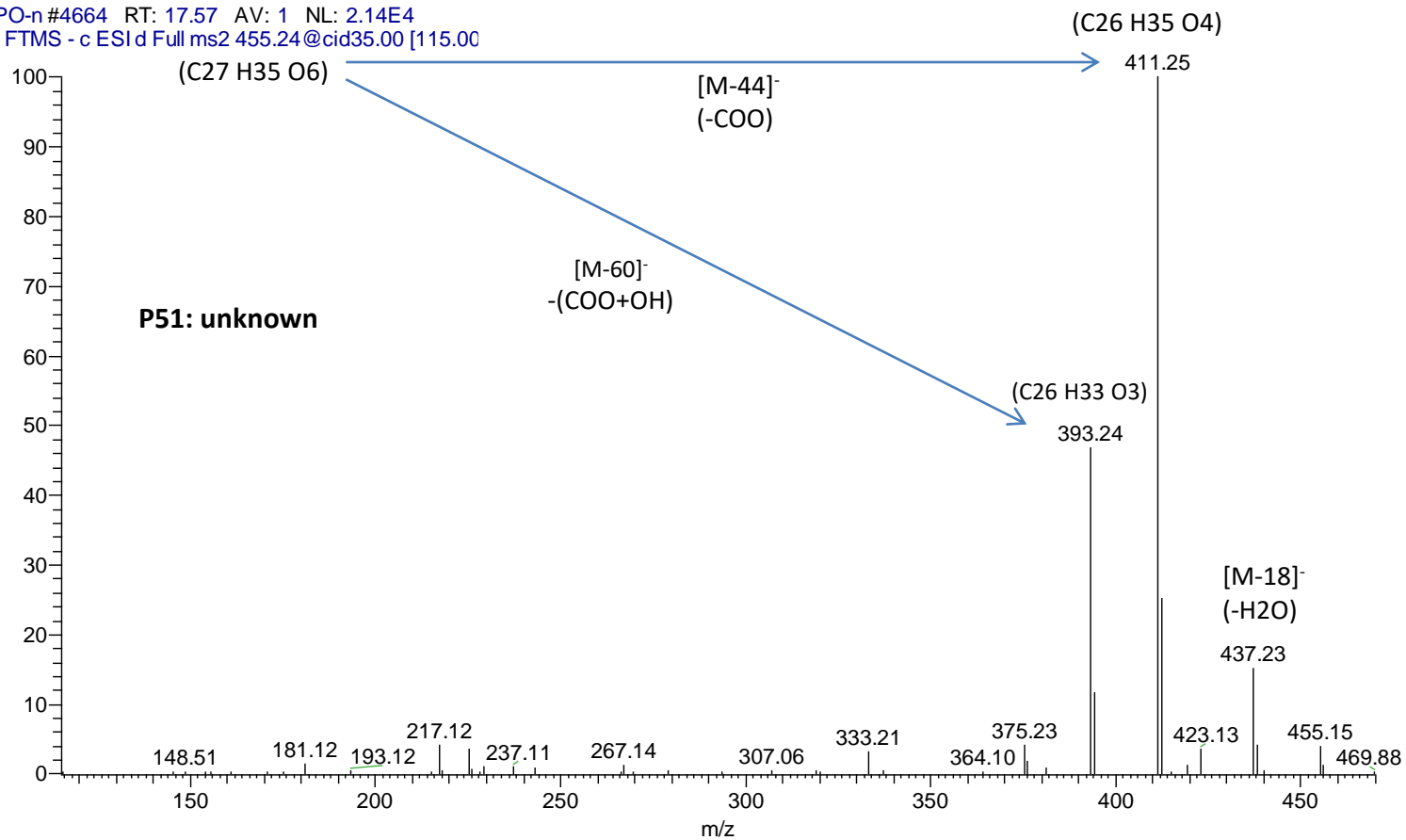

S34 Fig

Supplement: S34 Fig — (PDF) [file pone.0302795.s034.pdf]

PPO-n #4676 RT: 17.61 AV: 1 NL: 3.10E4  
T: FTMS - c ESI d Full ms2 445.13@cid35.00 [110.00]

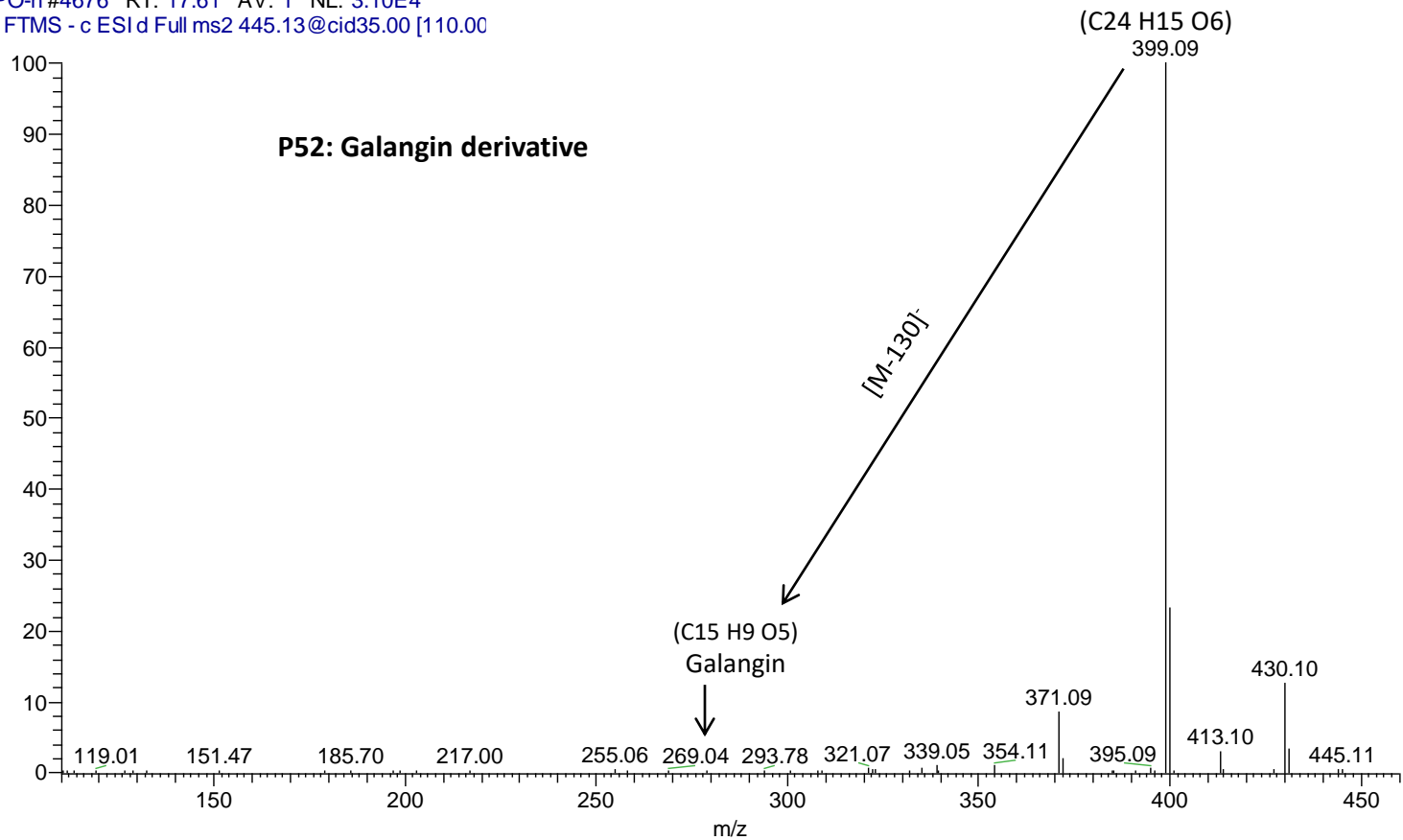

S35 Fig

Supplement: S35 Fig — (PDF) [file pone.0302795.s035.pdf]

PPO-n #4698 RT: 17.70 AV: 1 NL: 2.78E5  
T: FTMS - c ESI d Full ms2 355.12@cid35.00 [85.00-;

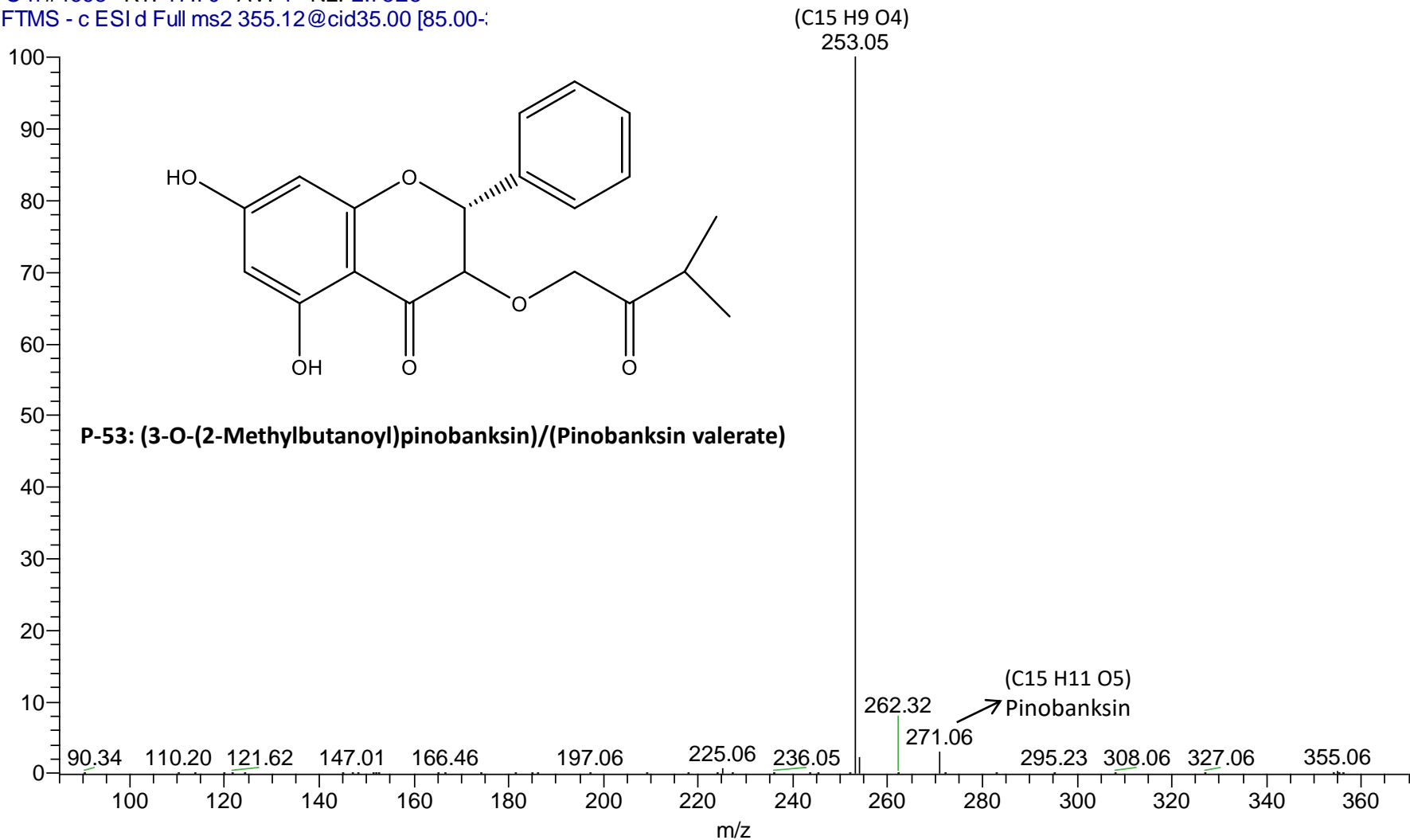

S36 Fig

Supplement: S36 Fig — (PDF) [file pone.0302795.s036.pdf]

PPO-n #4746 RT: 17.88 AV: 1 NL: 8.86E4  
F: FTMS - c ESI d Full ms2 431.15@cid35.00 [105.00]

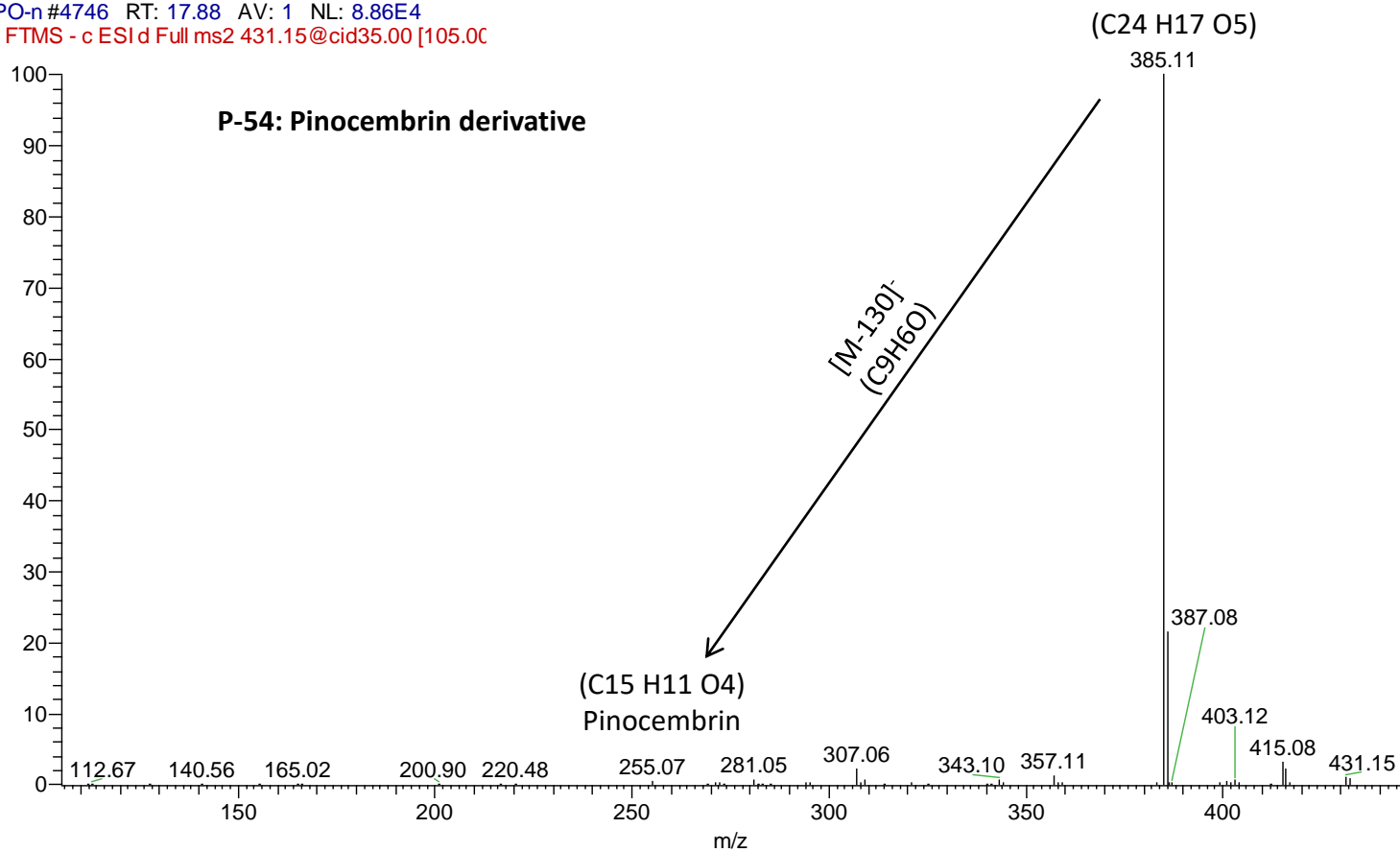

**S37 Fig**

Supplement: S37 Fig — (PDF) [file pone.0302795.s037.pdf]

PPO-n #4779 RT: 18.01 AV: 1 NL: 6.80E5  
T: FTMS - p ESI Full ms [100.00-1500.00]

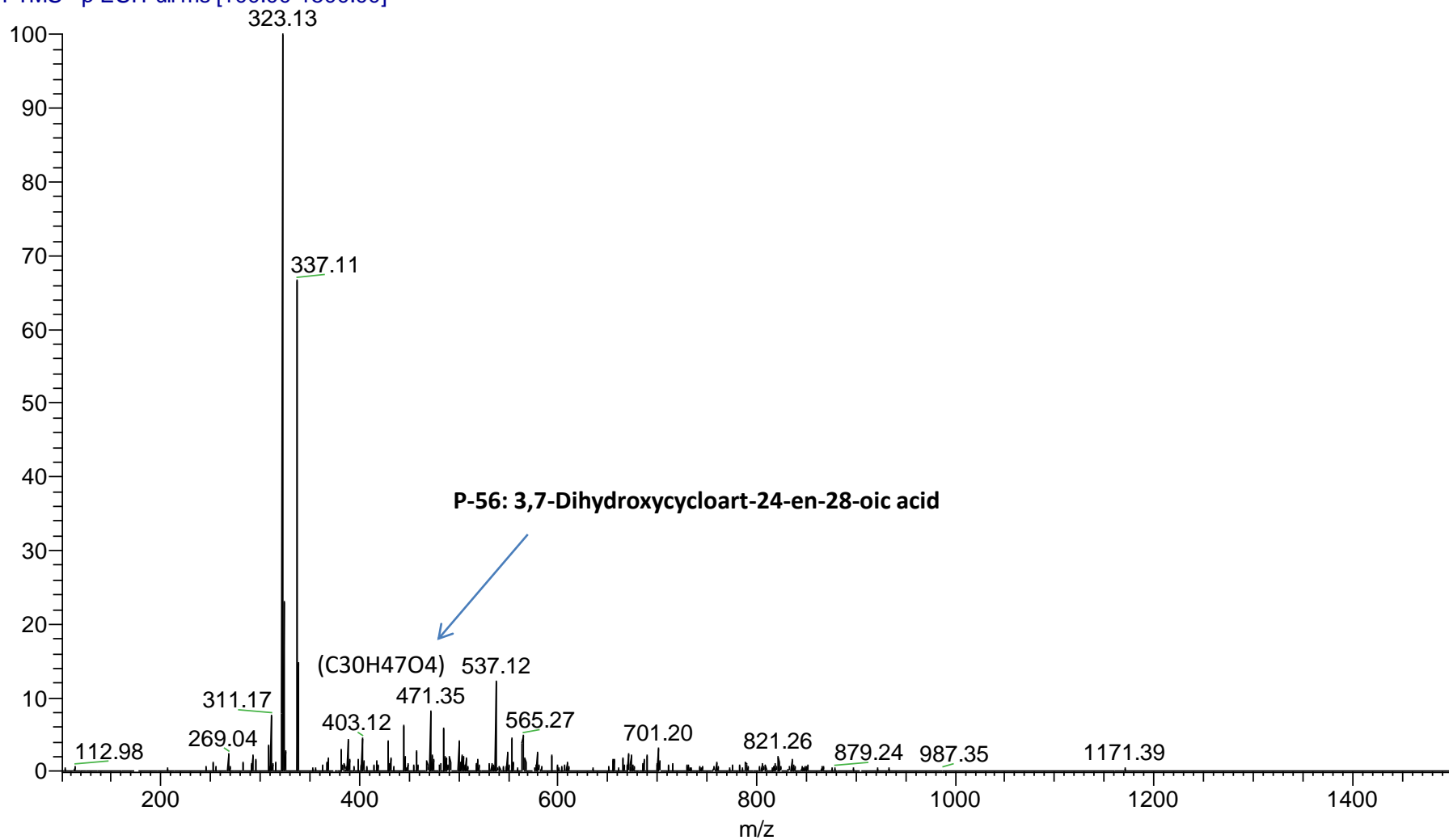

S38 Fig

Supplement: S38 Fig — (PDF) [file pone.0302795.s038.pdf]

PPO-n #4790 RT: 18.06 AV: 1 NL: 6.09E4  
T: FTMS - c ESI d Full ms2 337.11 @cid35.00 [80.00-:

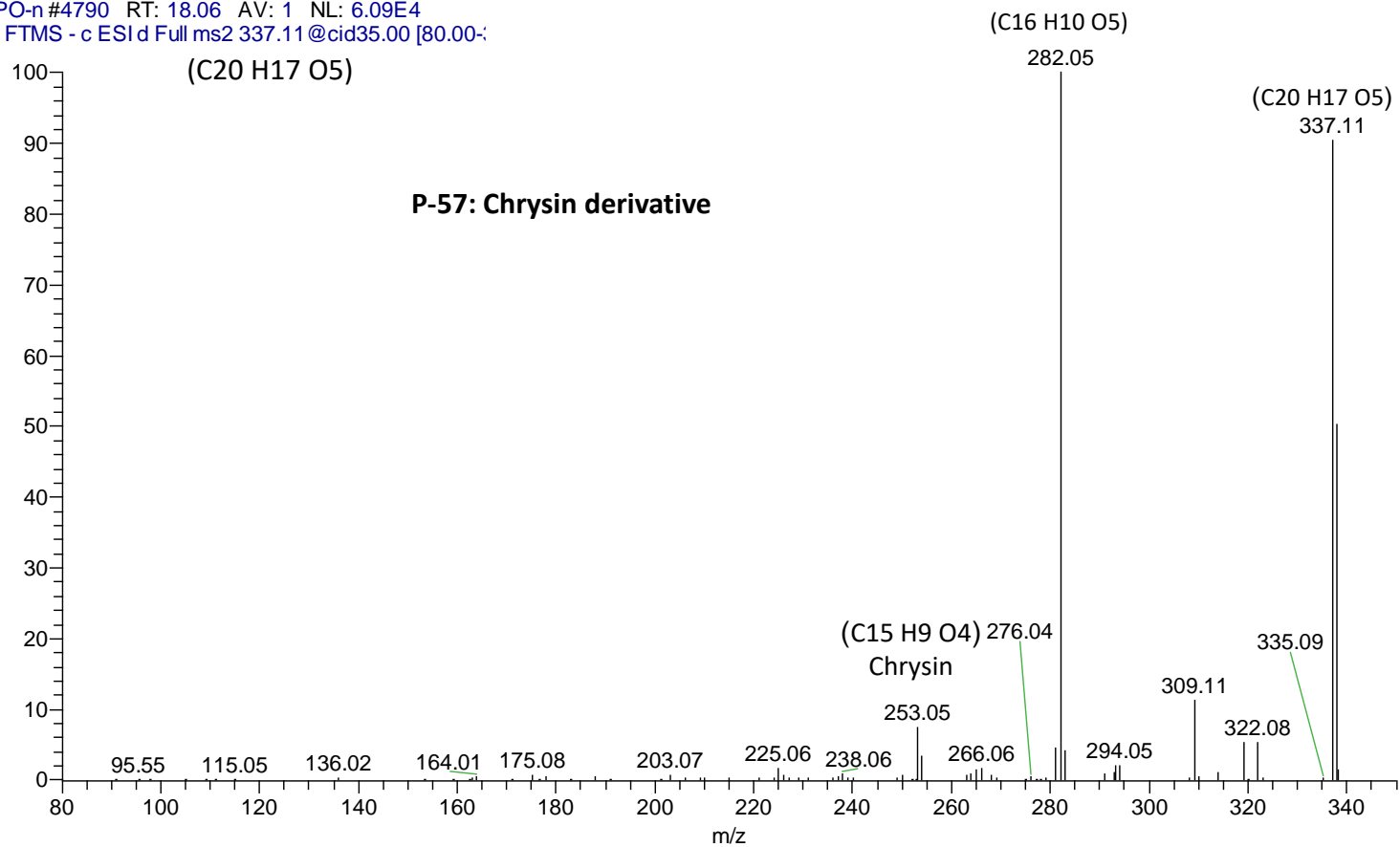

**S39 Fig**

Supplement: S39 Fig — (PDF) [file pone.0302795.s039.pdf]

PPO-n #4806 RT: 18.12 AV: 1 NL: 1.50E5  
F: FTMS - c ESI d Full ms2 369.11 @cid35.00 [90.00-

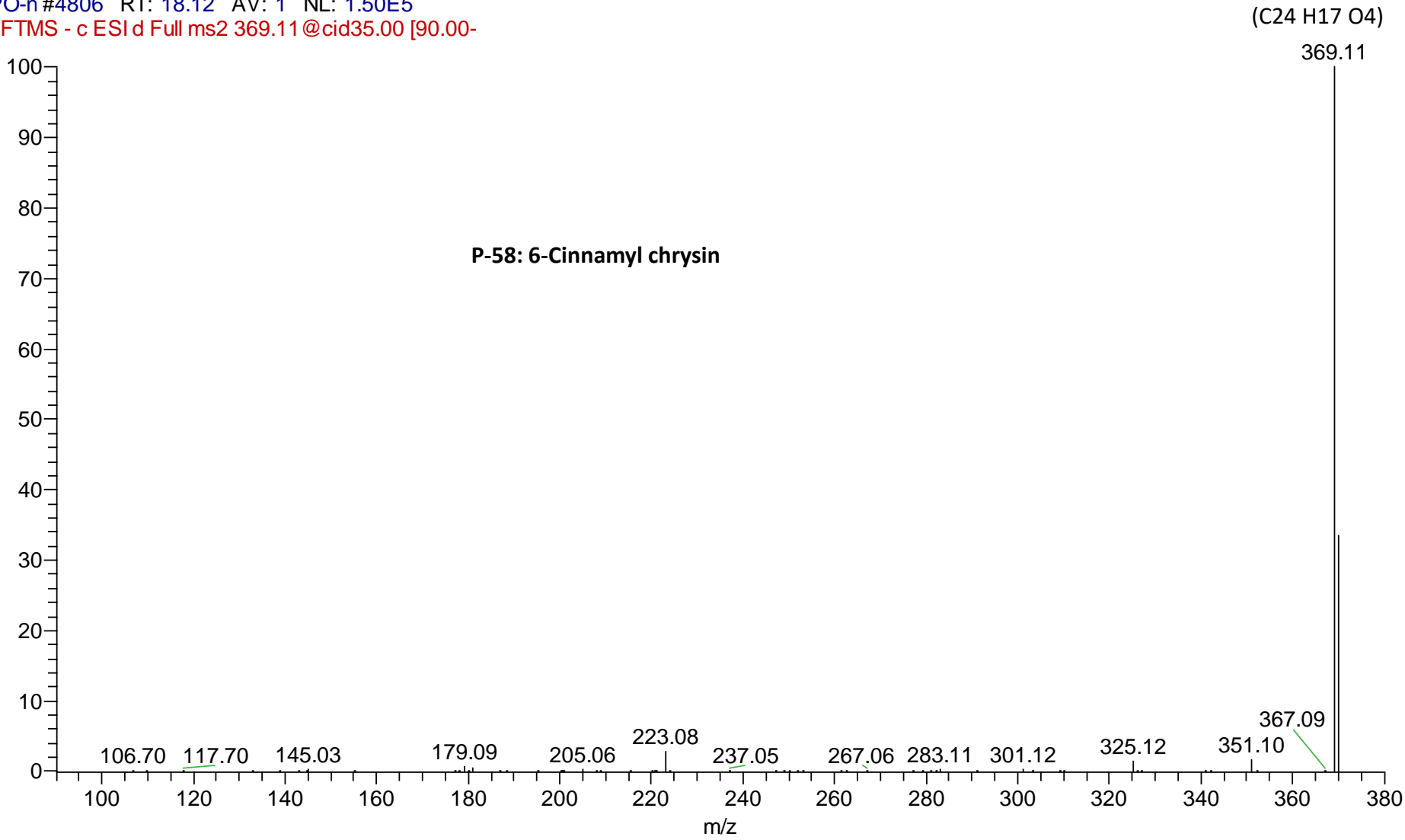

S40 Fig

Supplement: S40 Fig — (PDF) [file pone.0302795.s040.pdf]

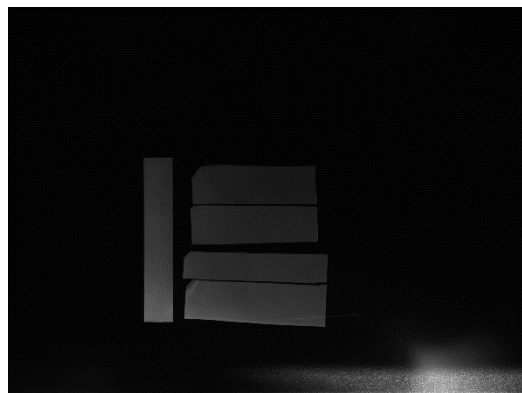

**a**

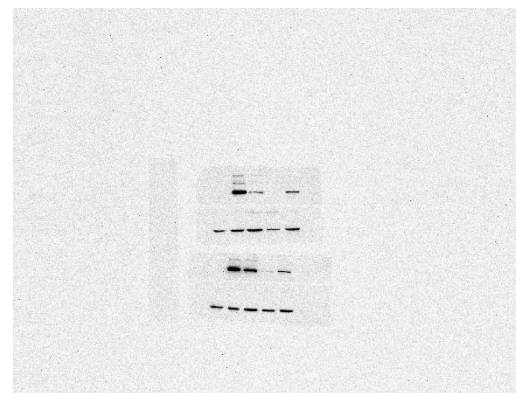

**b**

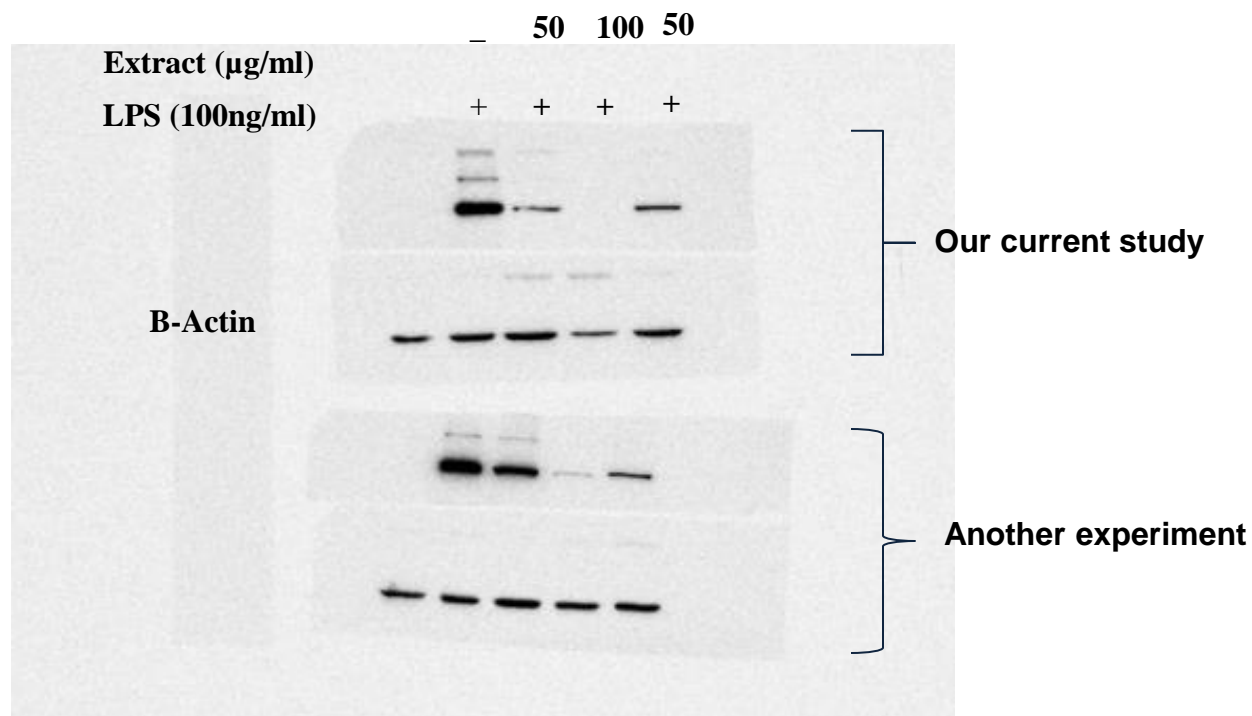

**c**

**S41 Fig**

Supplement: S41 Fig — (PDF) [file pone.0302795.s041.pdf]
