# Supplementary material for: In vitro and in vivo burn healing study of standardized propolis: Unveiling its antibacterial, antioxidant and anti-inflammatory actions in relation to its phytochemical profiling
Source: PLoS One. 2024 May 14;19(5):e0302795. doi: 10.1371/journal.pone.0302795 (PMC11093344; doi:10.1371/journal.pone.0302795)
Supplement: S28 Fig — (PDF) [file pone.0302795.s028.pdf]

P-43: 5,7-Dihydroxy-3-propanoyloxyflavanone.
(3-*O*-Propanoylpinobanksin)

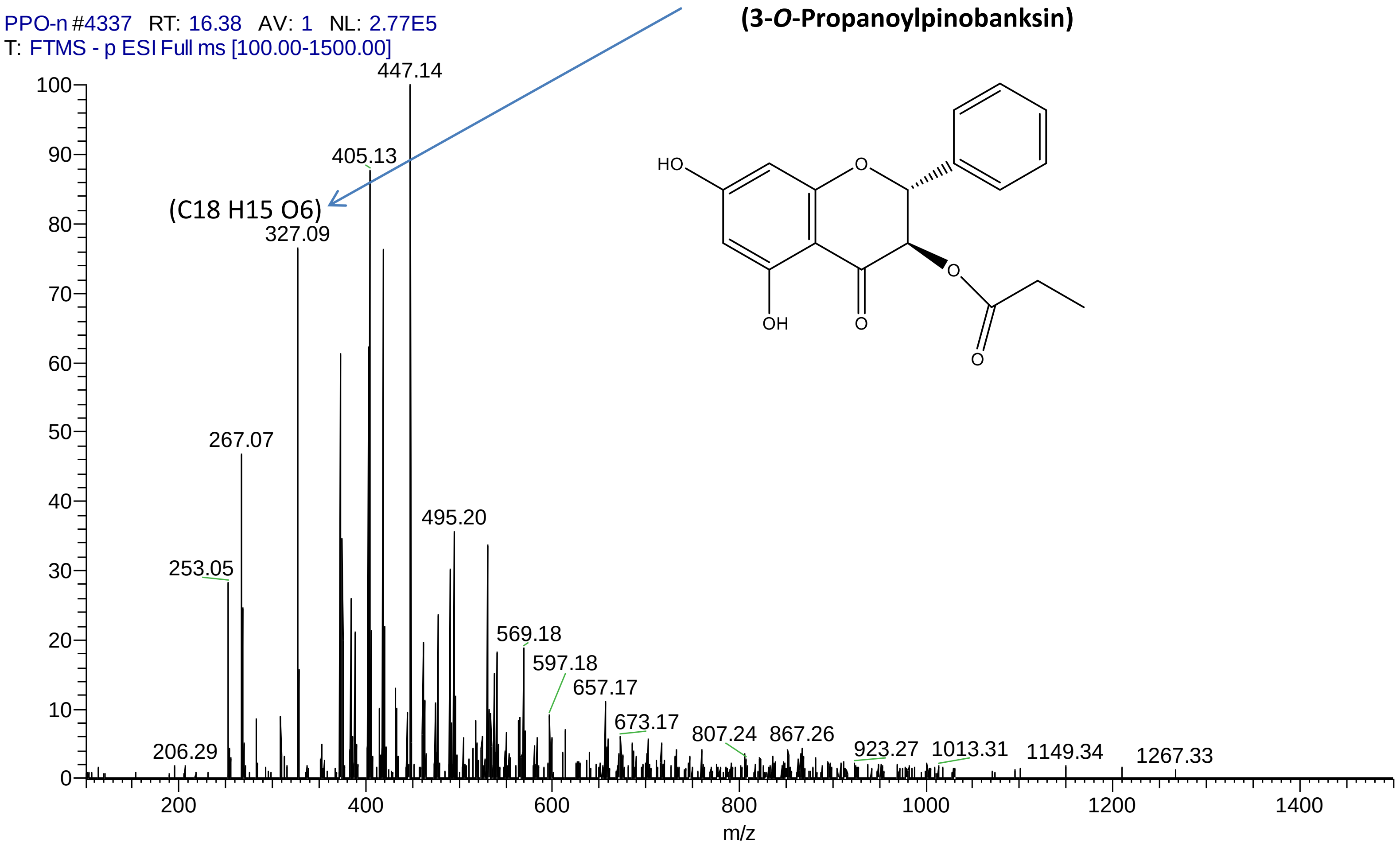

S28 Fig
