# Supplementary material for: In vitro and in vivo burn healing study of standardized propolis: Unveiling its antibacterial, antioxidant and anti-inflammatory actions in relation to its phytochemical profiling
Source: PLoS One. 2024 May 14;19(5):e0302795. doi: 10.1371/journal.pone.0302795 (PMC11093344; doi:10.1371/journal.pone.0302795)
Supplement: S1 File — (DOCX) [file pone.0302795.s042.docx]

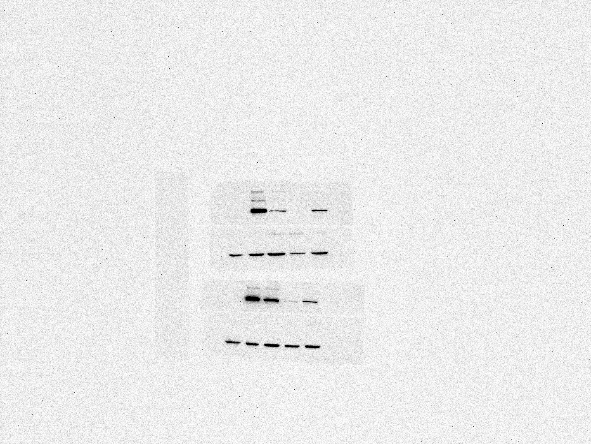


**b**

**a**

**S41 Fig represents the blot (a) and chemi raw images (b and c labelled) of our current study “the upper 2 strips” and the lower 2 strips belongs to another work since the images are uncropped, unprocessed and unsliced.**

**In next sections, we attach the metadata file of both images for your kind information.**

**Metadata file of Figure (1): blot image upper 2 strips.TIF**


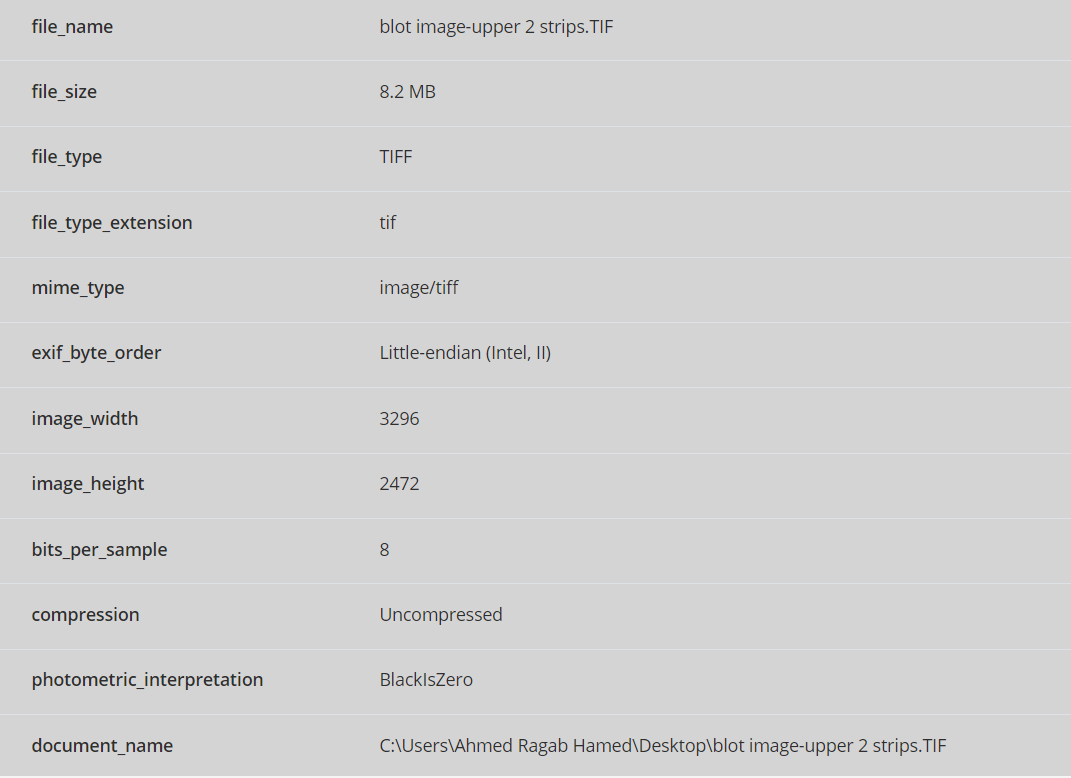


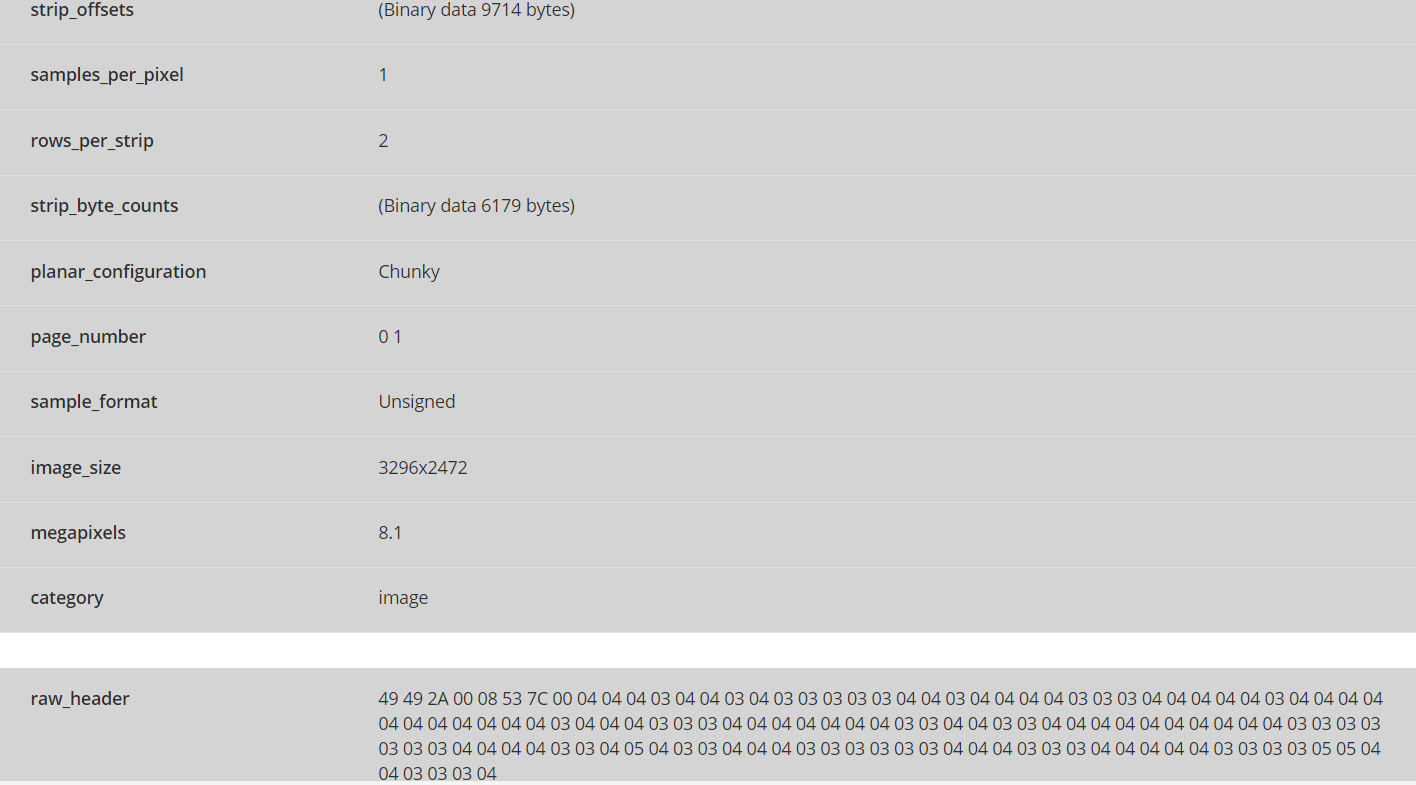


<https://www.metadata2go.com/result#j=20666218-db9c-42d0-a51b-4a8e0f5358e8&qr=true>

**Metadata file of Figure (2): Chemi_raw_image_upper upper 2 strips.TIF**


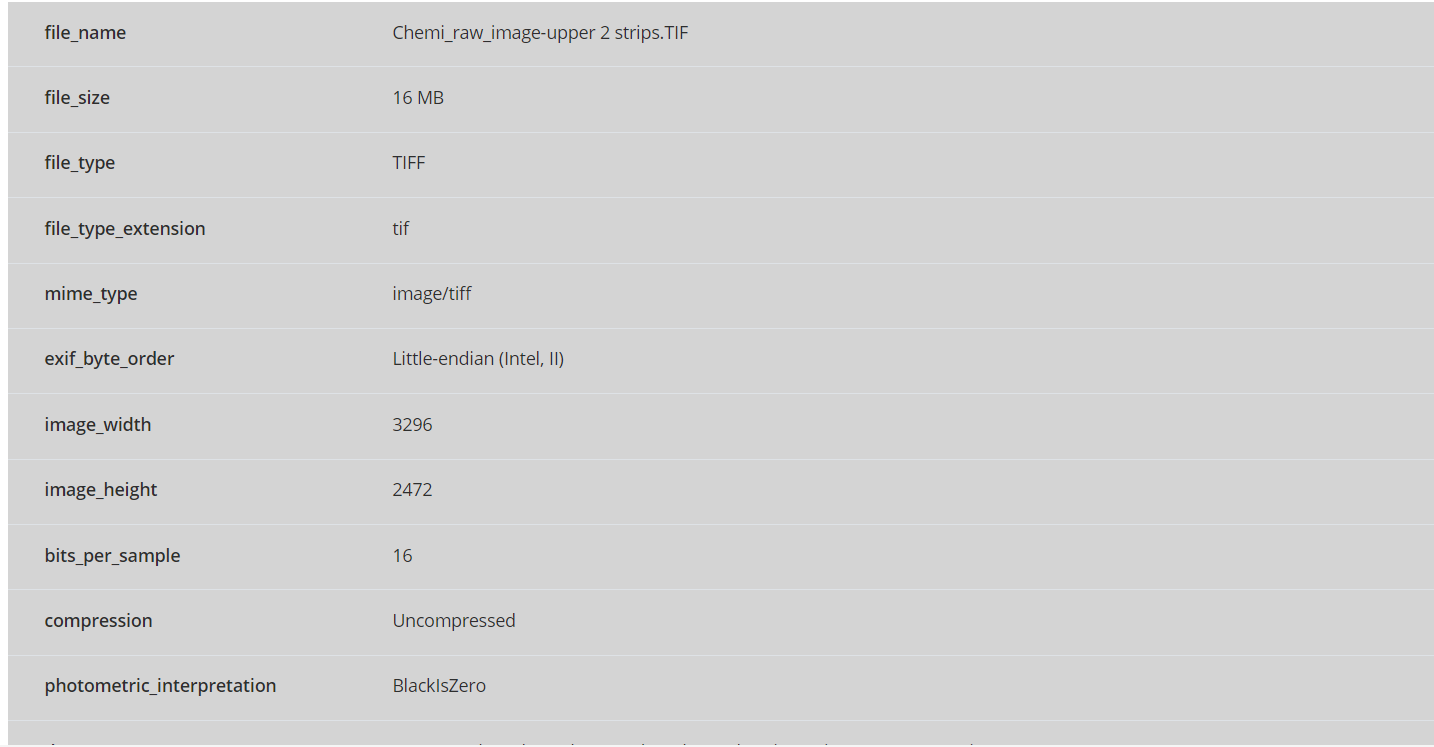


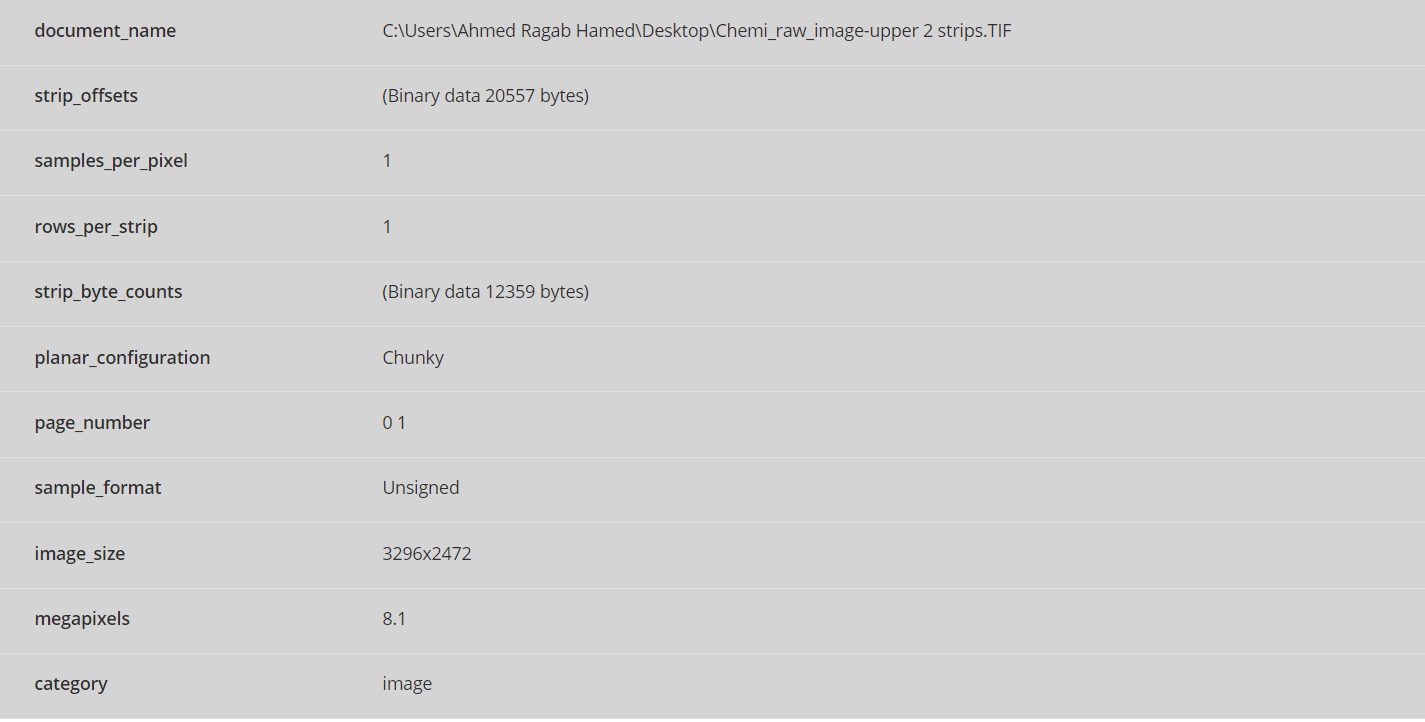


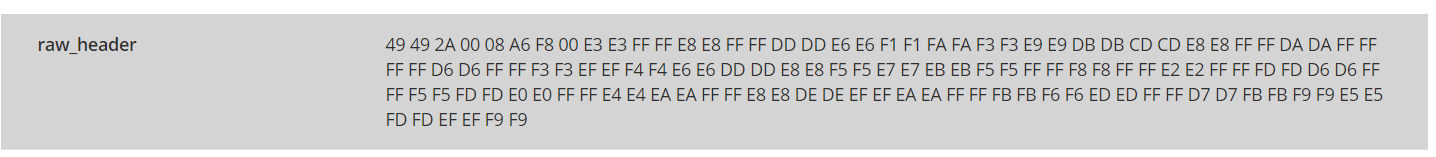


<https://www.metadata2go.com/result#j=22c09ee0-8c67-4ba9-8a71-3ad06f3ec0c4&qr=true>
